# Composition and Evolution of the Vertebrate and Mammalian Selenoproteomes

**DOI:** 10.1371/journal.pone.0033066

**Published:** 2012-03-30

**Authors:** Marco Mariotti, Perry G. Ridge, Yan Zhang, Alexei V. Lobanov, Thomas H. Pringle, Roderic Guigo, Dolph L. Hatfield, Vadim N. Gladyshev

**Affiliations:** 1 Brigham and Women's Hospital and Harvard Medical School, Boston, Massachusetts, United States of America; 2 Center for Genomic Regulation and Universitat Pompeu Fabra, Barcelona, Spain; 3 Department of Biochemistry and Redox Biology Center, University of Nebraska, Lincoln, Nebraska, United States of America; 4 Key Laboratory of Systems Biology, Shanghai Institutes for Biological Sciences, Chinese Academy of Sciences, Shanghai, China; 5 Sperling Foundation, Eugene, Oregon, United States of America; 6 Laboratory of Cancer Prevention, National Cancer Institute, National Institutes of Health, Bethesda, Maryland, United States of America; Ecole Normale Supérieure de Lyon, France

## Abstract

**Background:**

Selenium is an essential trace element in mammals due to its presence in proteins in the form of selenocysteine (Sec). Human genome codes for 25 Sec-containing protein genes, and mouse and rat genomes for 24.

**Methodology/Principal Findings:**

We characterized the selenoproteomes of 44 sequenced vertebrates by applying gene prediction and phylogenetic reconstruction methods, supplemented with the analyses of gene structures, alternative splicing isoforms, untranslated regions, SECIS elements, and pseudogenes. In total, we detected 45 selenoprotein subfamilies. 28 of them were found in mammals, and 41 in bony fishes. We define the ancestral vertebrate (28 proteins) and mammalian (25 proteins) selenoproteomes, and describe how they evolved along lineages through gene duplication (20 events), gene loss (10 events) and replacement of Sec with cysteine (12 events). We show that an intronless selenophosphate synthetase 2 gene evolved in early mammals and replaced functionally the original multiexon gene in placental mammals, whereas both genes remain in marsupials. Mammalian thioredoxin reductase 1 and thioredoxin-glutathione reductase evolved from an ancestral glutaredoxin-domain containing enzyme, still present in fish. Selenoprotein V and GPx6 evolved specifically in placental mammals from duplications of SelW and GPx3, respectively, and GPx6 lost Sec several times independently. Bony fishes were characterized by duplications of several selenoprotein families (GPx1, GPx3, GPx4, Dio3, MsrB1, SelJ, SelO, SelT, SelU1, and SelW2). Finally, we report identification of new isoforms for several selenoproteins and describe unusually conserved selenoprotein pseudogenes.

**Conclusions/Significance:**

This analysis represents the first comprehensive survey of the vertebrate and mammal selenoproteomes, and depicts their evolution along lineages. It also provides a wealth of information on these selenoproteins and their forms.

## Introduction

Selenocysteine (Sec)-containing proteins (selenoproteins) have been identified in all domains of life [1]–[3]. In these proteins Sec is encoded by UGA, a codon typically used for termination of protein synthesis. Sec insertion is possible when a stem-loop structure, the Sec insertion sequence (SECIS) element, is located in the 3′-untranslated regions (UTRs) of selenoprotein genes in eukaryotes and archaea, and immediately downstream of Sec-encoding UGA codon in the coding regions of bacterial selenoprotein genes [4]–[8]. A set of selenoproteins in an organism is known as the selenoproteome. The human selenoproteome is encoded in 25 selenoprotein genes, whereas 24 selenoprotein genes were found in mouse [9].

The largest and the best studied selenoprotein families are glutathione peroxidase (GPx), thioredoxin reductase (TR) and iodothyronine deiodinase (Dio) families, with 5, 3, and 3 Sec-containing genes in the human genome, respectively. The function of approximately half of mammalian selenoproteins is not known. Among the functionally characterized selenoproteins, many have a role in redox regulation. In mice, at least three selenoproteins, cytosolic/nuclear TR (TR1, Txnrd1), mitochondrial TR (TR3, Txnrd2) and glutathione peroxidase 4 (GPx4, Phgpx), are essential [10]–[12] and several others, when knocked out, resulted in reduced fitness or disease [13]–[16]. Additionally, selenoproteins have been implicated in cancer prevention, modulation of the aging process, male reproduction, and immune response [17]–[21]. The mammalian selenoproteins can be broadly classified into two classes: housekeeping and stress-related [22]. Housekeeping selenoproteins are less affected by dietary selenium (Se) status and often serve functions critical to cell survival, whereas stress-related selenoproteins are not essential for survival and often show decreased expression in Se-deficient conditions.

Previous analyses of the selenoproteome in various model organisms have revealed widely different selenoprotein sets. For example, some green algae and vertebrates have more than 20 selenoproteins, whereas red algae, insects and nematodes less than 5, and higher plants and yeast do not have any [23]. Recent studies also showed that aquatic organisms generally have larger selenoproteomes than terrestrial organisms, and that mammalian selenoproteomes show a trend toward reduced use of selenoproteins [24], [25]. However, whereas a variety of organisms have been analyzed for selenoprotein occurrence [24]–[32], a comprehensive survey of the vertebrate or the mammalian selenoproteomes has not been done.

The aim of this work was to address questions regarding Se utilization and evolution of selenoproteins in vertebrates, focusing on mammals. We used both genomic sequences and other diverse datasets to analyze the composition, evolution, and properties of mammalian and other vertebrate selenoproteomes. We characterized the origin and loss of each selenoprotein from fish to mammals and report a comprehensive analysis of each of these proteins that revealed novel insights into the use of Sec in these organisms.

## Results

### Identification and comparative analysis of vertebrate selenoproteomes

**Figure 1:**
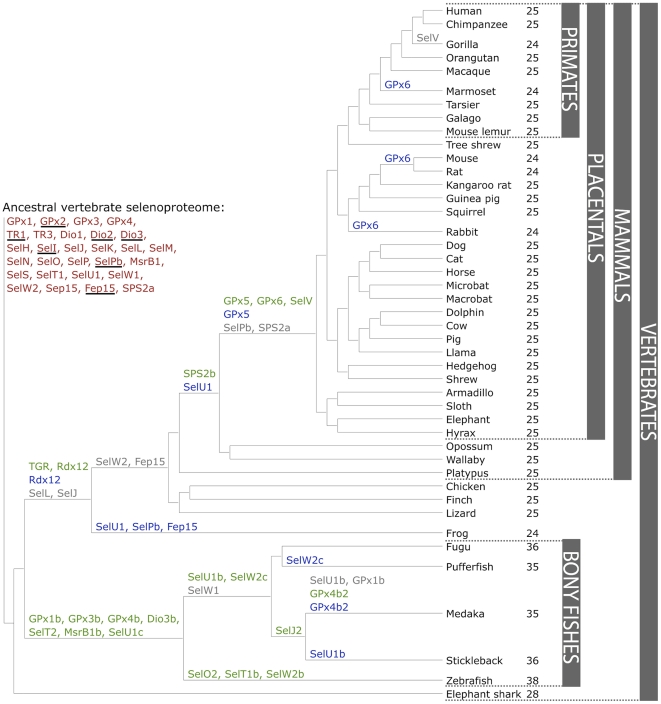
Evolution of the vertebrate selenoproteome. The ancestral vertebrate selenoproteome is indicated in red, and its changes across the investigated vertebrates are depicted along their phylogenetic tree. The ancestral selenoproteins found uniquely in vertebrates are underlined. The creation of a new selenoprotein (here always by duplication of an existing one) is indicated by its name in green. Loss is indicated in grey. Replacement of Sec with Cys is indicated in blue (apart from SelW2c in pufferfish, which is with arginine). Events of conversion of Cys to Sec were not found. On the right, the number of selenoproteins predicted in each species is shown.

We characterized vertebrate selenoproteomes by searching for all known selenoproteins in Trace Archive, non-redundant, expressed sequence tag (EST), and genomic databases of 44 vertebrates (including 34 mammals) (Figure 1 and Supplementary Table S1). The search was supplemented with the analysis of SECIS elements via SECISearch [9], and with the subsequent phylogenetic analysis of proteins belonging to the same superfamily. Overall, the searches yielded 45 selenoproteins (selenoprotein subfamilies) in sequenced vertebrates, 28 of which were found in mammals (Table 1). However, none of the mammals analyzed contained all these proteins: at most, 25 selenoproteins were detected. The largest selenoproteomes were found in bony fishes, with a maximum of 38 selenoproteins in zebrafish. The smallest selenoproteome (24 selenoprotein genes) was predicted in frog and in some mammals (Figure 1). 21 selenoproteins were found in all vertebrates: GPx1-4, TR1, TR3, Dio1, Dio2, Dio3, SelH, SelI, SelK, SelM, SelN, SelO, SelP, MsrB1 (methionine-R-sulfoxide reductase 1), SelS, SelT1, SelW1, Sep15. The other selenoproteins were found only in certain lineages, highlighting a dynamic process by which new selenoprotein genes were generated by duplication, while others were lost or replaced their Sec with cysteine (Cys). The predicted ancestral vertebrate selenoproteome is indicated in Figure 1, along with the details of its transformations across vertebrates. We found 28 proteins in the ancestral vertebrate selenoproteome and 25 in the ancestral mammalian selenoproteome.

**Table 1:** Vertebrate Selenoproteins.

Selenoproteins	Commonly used abbreviations	Fish	Frog	Birds	Mammals		
					Platypus	Marsupials	Placentals
15 kDa selenoprotein	Sep15, Sel15	+	+	+	+	+	+
Fish 15 kDa selenoprotein-like	Fep15	+					
Glutathione peroxidase 1a	GPx1, GSHPx1, GPx, cGPx	+	+	+	+	+	+
Glutathione peroxidase 1b	GPx1b	+					
Glutathione peroxidase 2	GPx2, GSHPx-GI, GPRP, GI-GPx, GSGPx-2	+	+	+	+	+	+
Glutathione peroxidase 3	GPx3, pGPx, GPx-P, GSHPx-3, GSHPx-P, EGPx	+	+	+	+	+	+
Glutathione peroxidase 3b	GPx3b	+					
Glutathione peroxidase 4a	GPx4, PHGPx, MCSP, snGPx, snPHGPx, mtPHGPx	+	+	+	+	+	+
Glutathione peroxidase 4b	GPx4b	+					
Glutathione peroxidase 6	GPx6, OMP						+
Iodothyronine Deiodinase 1	Dio1, DI1, 5DI, TXDI1, ITDI1	+	+	+	+	+	+
Iodothyronine Deiodinase 2	Dio2, DI2, D2, 5DII, TXDI2, SelY	+	+	+	+	+	+
Iodothyronine Deiodinase 3a	Dio3, DI3, 5DIII, TXDI3	+	+	+	+	+	+
Iodothyronine Deiodinase 3b	Dio3b, DI3b	+					
Methionine-R-Sulfoxide Reductase 1a	MsrB1, SelR, SelX, SepR,	+	+	+	+	+	+
Methionine-R-Sulfoxide Reductase 1b	MsrB1b	+					
Selenophosphate Synthetase 2a	SPS2a, SEPHS2, Ysg3	+	+	+	+	+	
Selenophosphate Synthetase 2b	SPS2b					+	+
Selenoprotein H	SelH, SepH	+	+	+	+	+	+
Selenoprotein I	SelI, SepI	+	+	+	+	+	+
Selenoprotein J	SelJ	+					
Selenoprotein J2	SelJ2	+					
Selenoprotein K	SelK, SelG, SepK	+	+	+	+	+	+
Selenoprotein L	SelL	+					
Selenoprotein M	SelM, SepM	+	+	+	+	+	+
Selenoprotein N	SelN, SepN1, RSS, MDRS1, RSMD1	+	+	+	+	+	+
Selenoprotein O	SelO, SepO	+	+	+	+	+	+
Selenoprotein O2	SelO2	+					
Selenoprotein P	SelP, SeP, SepP1, Se-P, SelPa	+	+	+	+	+	+
Selenoprotein Pb	SelPb	+		+	+	+	
Selenoprotein S	SelS, VIMP, ADO15, SBBI8, SepS1, AD-015	+	+	+	+	+	+
Selenoprotein T1a	SelT1a, SepT	+	+	+	+	+	+
Selenoprotein T1b	SelT1b	+					
Selenoprotein T2	SelT2	+					
Selenoprotein U1a	SelU1, SepU1	+		+	+		
Selenoprotein U1b	SelU1b, SepU1b	+					
Selenoprotein U1c	SelU1c, SepU1c	+					
Selenoprotein V	SelV, SepV						+
Selenoprotein W1	SelW1, SeW, SepW1	+	+	+	+	+	+
Selenoprotein W2a	SelW2a	+	+				
Selenoprotein W2b	SelW2b	+					
Selenoprotein W2c	SelW2c	+					
Thioredoxin reductase 1	TR1, TxnRd1, TxnR, TrxR1, GRIM-12	+	+	+	+	+	+
Thioredoxin reductase 3	TR3, TR2, TxnRd2, SelZ, TrxR2, TR-Beta	+	+	+	+	+	+
Thioredoxin/glutathione reductase	TGR, TR2, TR3, TxnRd3, TrxR3		+	+	+	+	+

Selenoproteins detected by genomic searches in vertebrate genomes are shown. The groups for which a given selenoprotein was found in at least one organism are marked.

Several selenoproteins genes were found duplicated in all bony fishes investigated, probably owing to the whole genome duplication in the early evolution of ray-finned fishes [33]. This event generated selenoproteins GPx1b, GPx3b, GPx4b, Dio3b, SelT2, MsrB1b and SelU1c. Additionally, some gene duplications were observed only in specific lineages of bony fishes. In zebrafish only, we found additional copies of SelO, SelT1 and SelW2, named respectively SelO2, SelT1b, and SelW2b. In medaka and stickleback (Smegmamorpha), we identified a selenoprotein generated by a duplication of SelJ, which we named SelJ2. In Percomorpha (which include all bony fishes in this study apart from zebrafish), we observed a duplication of selenoprotein gene SelU1 generating SelU1b. In medaka, this gene was missing, while in stickleback Sec was replaced by Cys. Also in Percomorpha, we traced another duplication of SelW2, generating a selenoprotein gene that we named SelW2c. This protein lost Sec in pufferfish.

After the split with fishes, several selenoproteins were generated also in the lineage to mammals. These events are mentioned here, and their analysis will be detailed in the next section. Thioredoxin/glutathione reductase (TGR) evolved prior to the split of tetrapods through a duplication of an ancestral TR1 protein containing a glutaredoxin domain. SPS2b arose initially by a retrotransposition before the split of marsupials, while SelV and GPx6 appeared at the root of placental mammals by duplications of SelW and GPx3, respectively.

Several selenoproteins were lost across vertebrates after the terrestrial environment was colonized. This is consistent with the idea that mammals reduced their utilization of Sec compared with fishes [25]. Selenoproteins SelL and SelJ are today found only in fishes, among vertebrates. Fep15 (fish 15 kDa selenoprotein) was previously identified only in bony fishes [34]. We now identified this selenoprotein in the cartilaginous fish elephant shark and also found it as a Cys homolog in frog. These facts imply that Fep15 was a part of the ancestral vertebrate selenoproteome and was lost prior to the split of reptiles. Selenoprotein SelW2 was also lost approximately at the same point, as we find it today only in fish and frog. Finally, before the split of placental mammals selenoproteins SPS2a and SelPb were lost. We observed a few selenoprotein losses also in bony fishes: SelW1 was lost in Percomorpha, and selenoproteins SelU1b and GPx1b were lost in medaka.

One process contributing to the reduction of selenoproteome is the conversion of Sec to Cys. This process is specific to selenoproteins and can be accomplished by a single point mutation can transform a Sec UGA into a Cys codon. However, it has to be noted that Sec and Cys are not functionally equivalent, and Cys conversions are not neutral, although the reasons are still unclear [32]. We observed 12 conversions to Cys along vertebrates, 8 of which happened after the split of mammals (Figure 1). Some were found common to many organisms and were mapped back to their common ancestor (e.g. SelU1 in mammals), while others were found in relatively narrow lineages, sometimes even in single species (e.g., GPx6 in marmoset).

### Comparative analyses of selenoprotein families

We built multiple sequence alignment for all vertebrate selenoproteins (Supplementary Figures S1, S2, S3, S4, S5, S6, S7, S8, S9, S10, S11, S12, S13, S14, S15, S16, S17, S18, S19, S20, S21, S22, S23, S24, S25, S26, S27, S28, S29, S30) and analyzed their phylogenetic relationships and sequence features. The most conserved selenoprotein was SelT, with an impressive identity across all mammals even at the nucleotide sequence level (Supplementary Figure S31). Below, we report our analysis for the selenoprotein families with most interesting findings.

#### Selenophosphate synthetase 2

**Figure 2:**
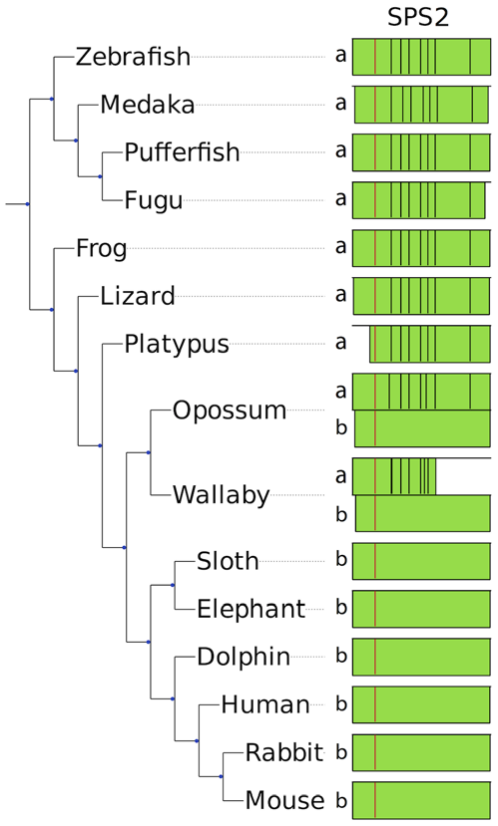
Replacement of a multiexon SPS2a by an intronless SPS2b. In the figure, the SPS2 genes found in some representative species are shown. The positions of introns along the protein sequence are displayed with black lines, and the Sec residue is displayed in red. In a few cases, the predicted genes were incomplete because of poor sequence data (e.g., the N-terminal region in platypus). Placental mammals (bottom) possess a single intronless gene, SPS2b. Non-mammalian vertebrates (top) and platypus possess a single multiexon gene, SPS2a. Marsupials (opossum and wallaby) possess both.

The function of selenophosphate synthetase 2 (SPS2) is to generate the Se donor compound (selenophosphate) necessary for Sec biosynthesis, and interestingly it is itself a selenoprotein. Although SPS2 was found as a selenoprotein in all vertebrates, we observed that a gene replacement took place. In mammals, the SPS2 gene appeared initially as a multiple exon gene (SPS2a), but was then replaced by a single exon copy (SPS2b). In monotremes and non-mammalian vertebrates, only SPS2a is present, in placental mammals only SPS2b is present, whereas marsupials still possess both genes (Figure 2). The protein alignment of SPS2a/b is provided in Supplementary Figure S22. In opossum, both SPS2a and SPS2b have strong SECIS elements (Supplementary Figure S32), and the distance between the stop codons and the SECIS element is comparable in the two versions (596 nucleotides in the single exon version and 555 nucleotides in the multi-exon version). UGA-to-SECIS distances are also comparable (1805 versus 1542 nucleotides, respectively). Due to lack of transcription data, we cannot be sure that both versions are active. In wallaby (another marsupial), we also detected both SPS2a and SPS2b genes. In this case though, we could not reconstruct the entire genes due to incomplete genome assembly. In addition, SPS2a sequence appears to contain a 2 bp insertion in the penultimate exon, which would result in a frameshift. Nonetheless, given the very high conservation of the gene also downstream of the insertion and the poor coverage of sequence data, we think that this is sequencing/assembly artifact and that the gene is intact and functional.

Overall, our results suggest that SPS2b arose by reverse transcription following the monotreme/marsupial split and eventually replaced SPS2a in placental mammals. Interestingly, opossum SPS2a is located on the X chromosome. Although it must be said that the number of available genomes assembled in chromosomes is quite limited, this is the only case in which we found an SPS2 gene on a mammalian sexual chromosome. This is almost unique also when considering all mammalian selenoprotein genes: the only exceptions are platypus GPx6 residing on chromosome X1 (though the sex chromosome system of monotremes is radically different from other mammals and is still poorly understood [35]) and a pseudogene of GPx1, described later, which is localized on chromosome X. Selenoproteins and Se pathways are linked to sex-specific traits [36]. It is known that the X chromosome is overrepresented with sex-specific genes, and is a preferred site for retrotranspositions both on and off [37]. It could be speculated that the retrotransposition generating SPS2b and its subsequent functionalization may have been a response to a previous chromosome rearrangement that brought the SPS2a gene on the chromosome X at the root of marsupials.

#### SelV and SelW

**Figure 3:**
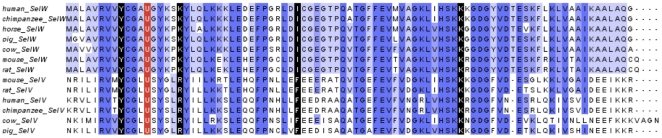
Multiple sequence alignment of SelV and SelW. The last 9 residues of SelV exon 1 and exons 2–5 are shown aligned to complete SelW sequences. The last residue of each exon is marked in black and the Sec in red.

SelV was the least conserved mammalian selenoprotein (Supplementary Figure S19) that likely arose from a duplication of SelW in the placental stem. The functions of SelV and SelW are not known, but SelV is expressed exclusively in testes [9], whereas SelW is expressed in a variety of organs. SelW and SelV exhibited the same gene structure; each contained 6 exons with intron locations and phases conserved. Coding regions were within exons 1–5. Exon 6 contained only the last portion of the 3′-UTR, including the SECIS element. Significant variation between SelW and SelV was found only in exon 1. Translated protein length of this exon has an average length of 261 residues (ranged from 228 amino acids in cat to 334 in dog), in contrast to SelW that had only 9 residues derived from exon 1 in most mammals. Only the last four residues of SelW and SelV in exon 1, which were located immediately upstream of the CxxU motif, were conserved; in contrast, their homology was high in exons 2–5 (Figure 3) as well as in the SECIS element in exon 6 (Supplementary Figure S28), suggesting that evolution of SelV by SelW gene duplication might have followed up by the addition of N-terminal sequences. Additional changes were observed in the last exon (exon 6, Supplementary Figure S33). First, a shift in the 5′ splicing site of SelV exon 6 was identified, with the effect of shortening the sequence preceding the SECIS element in this exon. SelW exon 6 had an average of 38 nucleotides from the beginning to the SECIS core, in contrast to SelV which had an average of 13 nucleotides. Second, compared to SelW, a substantial portion of the 3′-UTR downstream of the SECIS element was lost in SelV. Both changes resulted in a much shorter 3′-UTR in SelV (152 nucleotides on average) than SelW (358 nucleotides).

In a recent paper, it was reported that SelV was lost by deletion specifically in gorilla [38]. Our results confirm this finding. Indeed, we did not find SelV in any available sequences from this organism. Also, we could identify the region in the gorilla genome syntenic to the human SelV gene: consistent with a gene-specific deletion, the neighboring genes were present and conserved, while SelV was missing.

Several SelW homologs were observed across non-mammalian vertebrates. Phylogenetic analysis revealed a distinct group of proteins, SelW2. We found SelW2 as a selenoprotein in bony fishes, but also in frog and in elephant shark, which suggests that it was part of the ancestral vertebrate selenoproteome. In mammals, only a remote homolog of SelW2 is present: Rdx12 [39], which is not a selenoprotein and aligns a Cys to the Sec residue of SelW2. Frog is the only species in which we found both selenoprotein SelW2 and Rdx12. In all other tetrapods, we found only Rdx12. Thus we hypothesize that before the split of amphibians SelW2 duplicated and was immediately converted to a Cys form generating Rdx12, and then SelW2 was lost prior to the split of reptiles.

In bony fishes, we observed multiple copies of SelW2, whose phylogenetic relationships are very hard to entangle. Zebrafish had two copies of SelW2 (SelW2a, SelW2b), both selenoproteins, located in tandem on chromosome 3. The rest of bony fishes (Percomorpha) had a SelW2 protein similar to both SelW2a and SelW2b, plus a second protein located on a different chromosome (or scaffold), which we named SelW2c. In contrast, they all appear to have lost SelW1. Phylogenetic analysis shows that SelW2c proteins do not cluster with SelW2b, with Rdx12 or with SelW1 (Supplementary Figure S34). We think that most likely the SelW2a/b tandem duplication was specific to zebrafish, and that SelW2c was generated by another duplication of SelW2 at the root of Percomorpha, more or less concomitant with the SelW1 loss. Nonetheless, we cannot exclude a possibility that SelW2c is actually one of the two genes SelW2a/b or SelW1, which would have increased abruptly the sequence divergence rate in Percomorpha, confounding the phylogenetic reconstrunction. Interestingly, SelW2c in pufferfish is not a selenoprotein: the Sec codon was mutated to an arginine codon (CGA) and SECIS element was lost or degenerated. Therefore, the CxxU domain that is present in all SelW, SelW2 and SelV proteins is CxxR in this protein. We found evidence of the expression of this gene in ESTs.

#### Glutathione peroxidases

**Figure 4:**
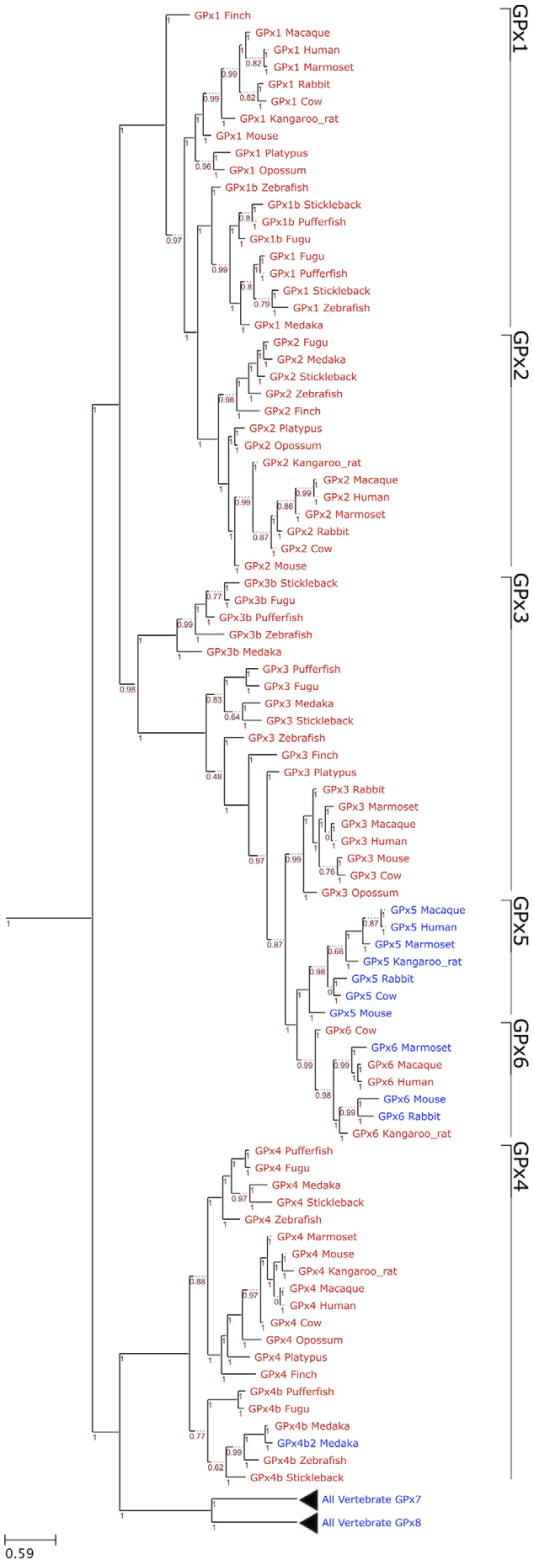
Phylogenetic tree of GPx family in eukaryotes. The figure shows a ML tree computed using the JTT substitution model. In the phylogram, Sec-containing proteins are shown in red and Cys-containing homologs are shown in blue. The GPx families are indicated on the right. The distance scale in substitutions per position is indicated at the bottom left. The branch support is shown in red.

Glutathione peroxidases are the largest selenoprotein family in vertebrates. Mammals have 8 GPx homologs, 5 of which are selenoproteins: GPx1-4, GPx6. We present here an unambiguous phylogeny of the GPx tree wherein three evolutionary groups were observed: GPx1/GPx2, GPx3/GPx5/GPx6, and GPx4/GPx7/GPx8 (Figure 4). Our findings are consistent with another study that examined GPx evolution [31]. It appeared that Cys-containing GPx7 and GPx8 evolved from a GPx4-like selenoprotein ancestor, but this happened prior to separation of mammals and fishes. GPx5 and GPx6 are the most recently evolved GPxs, which appeared to be the result of a tandem duplication of GPx3 at the root of placental mammals. Interestingly, no Sec-containing GPx5 form could be identified. As phylogeny indicates that this protein evolved from a duplication of selenoprotein GPx3, the Sec to Cys displacement must have happened very early in the evolution of GPx5.

For GPx6, we observed several independent Cys conversions: in the primate marmoset, in rat and mouse, and in rabbit (Figure 1). We suggest that the Cys-containing GPx6 was not present in the last ancestor of rabbit and rodents because the Sec-containing GPx6 was observed in other rodents, such as squirrel, guinea pig, kangaroo rat. In bony fishes, we observed three GPx duplications, generating GPx1b, GPx3b and GPx4b. All investigated species of this branch were found to have these three genes, with the exception of medaka, which apparently lost GPx1b. In this same species, we found an additional Cys copy of GPx4b, that we named GPx4b2.

Each of the mammalian Sec-containing GPx genes was highly conserved. Four of the five had better than 80% nucleotide sequence identity, while GPx1 had ∼70% sequence identity within mammalian sequences. GPx4 was one of the most conserved selenoproteins with better than 90% nucleotide sequence identity. Furthermore, considering full length selenoprotein sequences (i.e., including signal peptides), GPx4 had the highest level of conservation of any selenoprotein.

#### Thioredoxin reductases

TRs control the redox state of thioredoxins, key proteins involved in redox regulation of cellular processes. Mammals have three TR isozymes: cytosolic TR1, _itochondrial TR3, and TGR. Only two of these, TR1 and TR3, were detected in fish genomes. We thus investigated the phylogenesis of TGR. Previous studies have revealed various transcript (splicing forms) and/or protein (isoforms) variants in each mammalian TR in mammals [40]–[46] (see reviews [47], [48]). All TR1 alternative splicing was upstream of the first coding exon (exon 1) of the major form of TR1. Upstream exons were given these letter designations, 5′ to 3′: U1, A, U2, B, C, D1, D2, E, F, G, and H. Among the many splicing forms of TR1, one coded for an N-terminal Grx domain (Grx-TR1) [40], [42]. This TR1 form was derived from alternative exons A, B, C, and E (followed by common exons), with translation beginning in exon A. We found that in fish the Grx domain is present in the major form of TR1. In mammals, the major form of TR1 lacked this domain, but this occurred in TGR and in the TR1 alternative isoform mentioned above. Notably, the Grx-TR1 isoform was absent in rodents (but its fossil sequences could be identified [40]).

Sequence-based phylogenetic analyses suggested that mammalian TGR and TR1 evolved by duplication of the protein that corresponds to fish TR1. TR1 and TGR first appeared together in amphibians. Comparing mammalian TGR and the Grx-TR1 form with fish TR1, we found significant homology among the three proteins (Supplementary Figure S35). In addition, exon and intron boundaries were the same in all three genes. Interestingly, zebrafish TR1 had higher homology to mammalian TGR than to mammalian Grx-TR1. On the other hand, synteny placed zebrafish TR1 together with mammalian TR1 based on conservation of the downstream gene (upstream genes were different in all three TR genes). Overall, the data suggests that mammalian TR1 and TGR evolved by gene duplication from the ancestral protein that is similar to fish TR1, and this happened prior to the appearance of amphibians. Some time after the duplication, the Grx domain was retained in TR1 only in an alternative isoform, which was lost in rodents.

Sequence analysis highlighted also an important change in the predicted active site of Grx domains of mammalian TGR. In Grx-TR1s, fish TR1s and amphibian, reptile, and bird TGRs, we find a conserved CxxC motif. In mammalian TGRs, the second Cys in the Grx domain of TGR was mutated to serine (CxxS motif). Additionally, we found an interesting form of Grx-TR1 in cow where the motif was CRC. In the CxC motif, the two Cys residues may form a catalytic disulfide bond, similar to fish and mammals.

Another interesting isoform of TR1 is one identified in a previous study, containing a thioredoxin-fold domain [40]. In this isoform, alternative exons B and H, or just H, were included upstream of exon 1 with translation beginning in exon H. While EST data were found for this version only in rodents, there was overwhelming sequence similarity of exon H that suggests its importance even if there is a lack of transcriptome data. Exon H, and by extension this isoform, was identified in all placental mammals, but was absent in early mammals and in the rest of vertebrates. One last isoform is worth mentioning: isoform 4 [40], consisting of exons D1, D2, and E (with translation beginning in exon D2), was found to already occur in chicken, and was easily identified by sequence similarity in many inspected mammals (horse, opossum, and all rodents). Furthermore, EST data from humans, cows, and chickens confirmed the widespread expression of this isoform in a variety of tissues.

#### Iodothyronine deiodinases

The iodothyronine deiodinases (Dio) regulate activation and inactivation of thyroid hormones [49]. There are three Dio enzymes known in mammals, all of which contain Sec: Dio1, Dio2, Dio3. The deiodinases possess a thioredoxin-fold and show significant intrafamily homology. As mentioned above, we found the protein Dio3 duplicated in all bony fishes (Dio3b). Dio3 irreversibly inactivates the thyroid hormone by deiodination of the inner tyrosyl ring [50]. Interestingly, all detected Dio3 genes (including Dio3b) are intronless. All other genes in vertebrates, apart from SPS2b, were found to consist of multiple exons.

Dio2 is an ER-resident protein which activates the thyroid hormone by deiodination of the outer tyrosyl ring [50]. An interesting feature in Dio2 is that its mRNA has a second in-frame UGA codon. It was previously found that, in a cell culture system, the second UGA could insert Sec when the first UGA codon was mutated [51]. We extended translation to the next stop codon (after the second UGA), which was located an additional 9 (all mammals with the exception of primates) to 21 (in primates only) nucleotides downstream, but the additional amino acids were not conserved (Supplementary Figure S36). Thus, it appears that the primary function of the second UGA is to serve as stop codon.

#### Selenoprotein I

**Figure 5:**
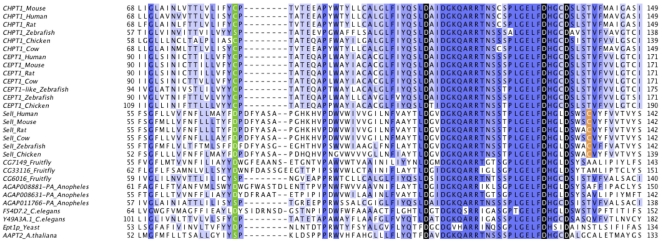
Multiple sequence alignment of SelI and its homologs. The multiple sequence alignment of the active site and preceding regions of CHPT1, CEPT1, and SelI is shown. Homologs are labeled with the annotated name. Proteins in the bottom section comprise a large group of diverse proteins containing the same domain. The most critical residues are marked in red. The residue in green marks the end of the first transmembrane domain. The cysteine residue near the active site emerged specifically in SelI proteins is marked in orange.The full length alignment is provided in Supplementary Figure S37 and the corresponding phylogenetic tree in Supplementary Figure S38.

Selenoprotein I (SelI) is one of the least studied selenoproteins. It contains a highly conserved CDP-alcohol phosphatidyltransferase domain. This domain is typically encountered in choline phosphotransferases (CHPT1) and choline/ethanolamine phosphotransferases (CEPT1). CHPT1 catalyzes the transfer of choline to diacylglycerol from CDP-choline [52]. CEPT1 catalyzes an analogous reaction but accepts both choline and ethanolamine. SelI has seven predicted transmembrane domains, which correspond to the predicted topologies of CHPT1 and CEPT1. The most critical portion of this structure is located between the first and second transmembrane domains, and there are three aspartic acids, which are critical for function. Figure 5 shows an alignment of the active site region of SelI and its closest sequence homologs. The full alignment is shown in Supplementary Figure S37, and a phylogenetic tree based on that alignment is shown in Supplementary Figure S38. Not only are the three aspartic acids conserved in all SelI proteins, but the entire active region is highly similar between SelI and its homologs. The most prominent difference between SelI and its homologs is a C-terminal extension in SelI, which contains Sec. The function of this extension is unknown. We were unable to find Cys forms with homology to the SelI C-terminal extension.

Sec residues are often involved in selenenylsulfide bonds with cysteines. Thus, we searched for cysteines emerged specifically in SelI proteins. We selected the cysteines completely conserved in SelI sequences and missing in all other sequence homologs. There were three such cysteines, at positions 133, 229 and 310 of human SelI (Supplementary Figure S37). The cysteine at position 133 (Figure 5) is the best candidate: it is predicted to reside on the same membrane side (internal) as the Sec, and it is also extremely close to the conserved aspartic acids.

In a recent work [53], human SelI protein was tested for CHPT1/CEPT1 enzymatic activities, reporting a specific ethanolamine phosphotransferase (EPT) activity. However, the authors used a bacterial expression system for purification of human SelI. Since eukaryotic SECIS elements are not recognized in bacteria, a truncated form of SelI was expressed, lacking the Sec residue and the rest of the C-terminus. Therefore, the function of intact SelI may be different, especially since the Sec residue of selenoproteins is known to be essential for function. Truncated forms of some selenoproteins, such as TR [54], show activity towards non-primary substrates. As truncated forms of selenoproteins are normally not observed in vivo, most of such activities are probably not biologically relevant. For these reasons, we believe that the real molecular function of SelI has yet to be discovered. One plausible possibility is that the EPT activity is just the first step in SelI function, with phosphatidylethanoloamine further processed in a Sec-dependent step. Another possibility is that the Sec extension provides completely different substrate specificity to SelI.

### Vertebrate-specific selenoproteins

We were interested to know what fraction of the vertebrate selenoproteome is found uniquely in vertebrates. Therefore, we searched all vertebrate selenoproteins in the sequenced basal chordates (amphioxus, tunicates), and, as a control, in any other sequenced eukaryotes as well. Among the ancestral 28 selenoproteins, 6 were detected uniquely in vertebrates: Fep15, GPx2, Dio2, Dio3, SelI, SelPb. Most of them (Fep15, GPx2, SelI, SelPb) showed at least partial conservation of intron structure with their closest homologs (Sep15, GPx1, CDP-alcohol phosphatidyltransferases, SelP, respectively). This may suggest that they were generated during the whole genome duplication occurred at the root of vertebrates [55]. These 6 selenoproteins, together with the 17 selenoproteins generated through duplication within vertebrates (GPx1b, GPx3b, GPx4b, GPx6, Dio3b, SelT1b, SelT2, MsrB1b, SelU1b, SelU1c, SelW2b, SelW2c, SelJ2, SelO2, TGR, SPS2b, SelV), constitute the set of vertebrate-specific selenoproteins.

### Analysis of UTRs, SECIS elements and UGA locations of mammalian selenoprotein genes

The untranslated regions (UTRs) of mRNAs are important sites where regulatory elements are typically found. 3′-UTRs are especially important for selenoprotein mRNAs as this is the location of SECIS elements in eukaryotes and archaea. We analyzed the lengths of 5′ and 3′-UTRs of mammalian selenoproteins (Supplementary Figures S39, S40). On average, the length of 5′-UTRs was 127 nucleotides, whereas that of 3′-UTRs was 1027 nucleotides. These observations fit the general characteristic of vertebrate mRNAs [56]. Dio2 had both the longest average 5′- and 3′-UTRs of all selenoprotein genes (409 and 5174 nucleotides, respectively). The shortest average 5′-UTR was observed in SelO, 61 nucleotides. SelV, despite having its 3′-UTR split into two exons, had the shortest 3′-UTRs with an average length of 152 nucleotides. We also examined selenoprotein lengths versus the UTR size, but did not observe significant correlation between them.

**Figure 6:**
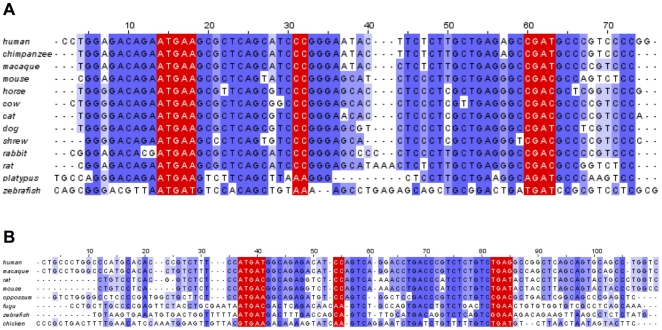
SECIS elements of SelM and SelO. Multiple sequence alignment of SelM (A) and SelO (B) SECIS elements. Critical regions are marked in red.

The SECIS element is present in all eukaryotic selenoprotein genes and is the fundamental signal for Sec insertion. While the overall stem-loop structure of the SECIS element is critical to its function, several especially important regions (and bases) have been identified. First, the base of the main stem has non-Watson-Crick interacting bases, known as the Quartet, or the core, including the invariant GA/GA pairs [5], [57], [58]. Next, in the apical loop, two unpaired bases are important for function, although their exact role is not known. In most cases, these two bases are AA. A comprehensive analysis of vertebrate SECIS elements showed that, as expected, almost all examined SECIS elements have the GA/GA quartet and AA in the apical loop. The exception included two selenoproteins, SelM and SelO, in which we found CC in the apical loop (Figure 6). The CC in SelM SECIS elements was only found in placental mammals, while all other vertebrates had AA. In SelO SECIS elements, the CC sequence was found in all mammals and in no other species. Thus, it appears that the CC forms of SECIS elements evolved specifically in mammals. Further analysis did not show any significant features that correlate with the presence of CC pattern.

The SECIS elements of most selenoprotein genes were wholly contained within the exon containing the stop codon. However, in several selenoprotein genes (SelH, SelT, SelV, SelW, and TR3), the 3′-UTR was split between two exons and the SECIS element was entirely located in the last exon. In one selenoprotein, SelK, the exon which contained the stop codon had a splice site immediately adjacent to the stop codon, and thus, the entire 3′-UTR was located in the next (last) exon. Finally, the two selenoproteins SelL and SelP have multiple Sec residues. The two Sec residues in SelL are only two residues apart and are inserted with the help of a single SECIS element [59]. SelP has a varying number of Sec residues and is unique in that it contains two SECIS elements. These two SECIS elements were separated by an average of 334 nucleotides and were always located in the same exon in the 3′-UTR in examined vertebrates.

To better understand general features of Sec insertion, we examined the distance between Sec-encoding UGA codons and SECIS elements (UGA-to-SECIS). Previous studies have attempted to define a minimal distance between these cis-elements in the mRNA. In one study performed on Dio1, the minimum spacing was defined as 51–111 nucleotides [60]. Other studies have shown that the location of the UGA can be varied within the gene and still maintain efficient UGA decoding, and that a SECIS element can be added to a non-selenoprotein 3′-UTR and an in-frame UGA be decoded as Sec [57], [61], [62]. We observed a wide range of UGA-to-SECIS distances (from 207 to 5207 nucleotides) for mammalian selenoproteins, all greater than the 51–111 base minimum. The average distance for all mammalian selenoproteins was 872 nucleotides. Dio2 and TR3 had the average longest and shortest UGA-to-SECIS distances, respectively.

### Identification of pseudogenes

Over the years, pseudogenes have been described for various selenoproteins, such as GPx1 [63], SelW [64], GPx4 [65], GPx2 [66], and Sep15 [67]. In our study, a total of 11 selenoprotein genes were found to be represented by additional pseudogenes in mammals (Table 2). Most of these pseudogenes had frameshifts or other mutations compromising their functionality. We observed a tendency for shorter selenoproteins to have more pseudogenes. The average length of selenoproteins with pseudogenes was 182 amino acids (10 kb genes), whereas selenoproteins which had no pseudogenes had an average length of 386 amino acids (24 kb genes).

**Table 2:** Mammalian selenoprotein pseudogenes.

Selenoprotein	# Pseudogenes	Organisms (# pseudogenes)
SelT	9	Human (2), Chimpanzee (2), Mouse (2), Rabbit (2), Horse
GPx1	3	Human (1), Squirrel (1), Rabbit (1)
GPx2	1	Human (1)
GPx4	4	Mouse (2), Rat (1), Microbat (1)
MsrB1	3	Human (1), Chimpanzee (1), Macaque (1)
SelH	5	Rat (1), Rabbit (1), Shrew (1), Hedgehog (1), Armadillo (1)
SelK	>27	Human (3), Chimpanzee (3), Macaque (3), Galago (2), Mouse (5), Rat (4), Guinea Pig (1), Squirrel (2), Dog (1), Cat (1), Microbat (2)
SelS	1	Hedgehog (1)
SelW	19	Human (2), Chimpanzee (2), Orangutan (1), Gibbon (1), Macaque (2), Rat (1), Cow (2), Dog (1), Cat (2), Microbat (1), Hedgehog (1), Elephant (1), Armadillo (2)
Sep15	5	Galago (1), Dog (1), Armadillo (2), Opossum (1)
SPS2b	4	Human (2), Macaque (1), Guinea Pig (1)

The selenoproteins with pseudogenes, the number of total pseudogenes identified in all mammals, and their occurrence in individual organisms are given.

**Figure 7:**
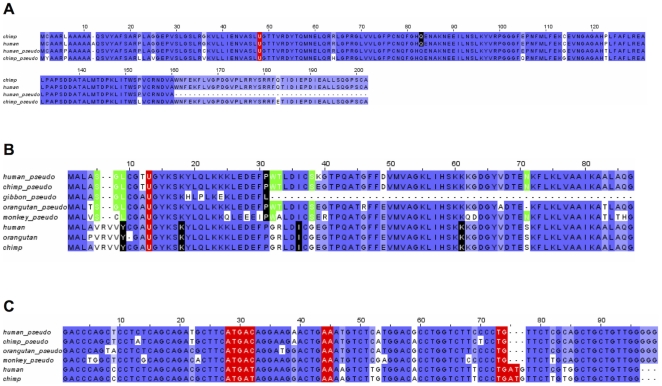
Multiple sequence alignment of selenoprotein genes and pseudogenes. A. GPx1. Multiple sequence alignment of human and chimpanzee GPx1 pseudogenes. B. SelW. The last residue of each exon is marked in black and Sec in red. Residues marked in green are described in the text. C. SECIS elements of SelW and SelW pseudogene.

Among the 11 selenoproteins with pseudogenes, SelK had more than any other selenoprotein (27 pseudogenes in 11 organisms), and rodents had the highest number. For example, mouse and rat had 5 and 4 SelK pseudogenes, respectively. SelW was another selenoprotein, which had many pseudogenes (19 in 13 organisms). An interesting GPx1 pseudogene was identified in humans and chimpanzees. The active site (surrounding the Sec) was conserved in both the functional and pseudogene versions of GPx1 and the overall conservation was quite high (Figure 7A). Three codon positions were particularly interesting (positions 6, 114, and 123). At each of these positions the residues translated from the pseudogenes matched, but were different than the residues in the corresponding position in GPx1. Therefore, it appeared the GPx1 pseudogene had been maintained since the human/chimpanzee split with few differences between the human and chimpanzee copies of the pseudogene. Furthermore, SECIS elements were also intact in these pseudogenes. However, a single base mutation at amino acid 161 in the human pseudogene sequence (TGG->TAG) resulted in a premature stop codon downstream of the active site. Due to this mutation and no supporting EST data, it is unlikely that this pseudogene is expressed. The Ka/Ks ratio (used as an indicator of the selective pressure) was 1.58 for this gene, which suggested the possibility of positive selection.

A similar case was observed with SelW. This pseudogene arose sometime after the split between marmoset and macaque, but before macaque split with subsequent primates. Consequently, this pseudogene was identified in macaque, gibbon (first exon of the pseudogene only), orangutan, chimpanzee and human. Several features of this pseudogene are peculiar. First, while the potential protein sequences of pseudogenes were highly homologous to SelW, the gene structure was different. The pseudogene consisted of two coding exons whereas SelW had five coding exons. The first exon of the pseudogene covered most of the first three coding exons of SelW and the second exon the remainder of SelW. Further analysis suggested that this gene was subject to positive selection. In Figure 7B, highlighted in green, are residues conserved in the pseudogenes but different in the corresponding positions in SelW. This situation occurred in 7 different positions in the pseudogenes. Similar to the GPx1 case above, this was indicative of constraint on the evolution of this gene. Furthermore, where nucleotide deletions occurred they happened in triplets thus preserving the reading frame. At positions 6 and 7 in the multiple sequence alignment, six bases were removed from the gene in all the pseudogenes. At position 71 in the alignment, the orangutan had a deleted amino acid, maintaining the reading frame. Additionally, the Ka/Ks ratio for human/macaque (the two most distant organisms in the dataset) was 0.59. Together, these data suggest that this pseudogene may have been under purifying selection for millions of years since it first appeared following the macaque/marmoset split.

However, further analysis suggested that this gene is not presently functional. First, the lack of ESTs in any of the representative organisms was inconsistent with this gene being expressed (or it only expresses in a very narrow niche). In addition, its SECIS element had a critical single base mutation. As discussed above, one of the salient features of SECIS elements is the GA/GA base-pairing in the stem loop necessary for binding of the proteins involved in Sec insertion [58]. An alignment of SECIS elements found in SelW and the pseudogenes (Figure 7C) showed that the pseudogene SECIS elements were missing the necessary GA sequence towards the end of the SECIS element. The SECIS elements from the pseudogenes still formed an appropriately shaped stem loop structure, but current evidence suggests that the missing GA should prevent Sec insertion. Together, this data suggests that while selection on this pseudogene has occurred, it is unlikely to be a currently active protein coding gene and that the in-frame UGA, if the gene was expressed, would result in early termination of translation.

### Identification of alternative splicing forms

Alternative splicing has previously been reported for several selenoproteins, including TR1 [40]–[42], [45]–[48], TR3 [43], Dio2 [68], Sep15 [67], and GPx4 [69], [70]. We examined ESTs for all mammalian selenoproteins to characterize alternative splicing forms of these proteins. A challenge to identify splicing variants is the dependence on the quantity of EST data available. Only six mammals (human, mouse, rat, macaque, dog, and cow) had a sufficient number of ESTs to provide useful information. We found an association between the number of ESTs available for an organism and the number of identified variants (Supplementary Figure S41), suggesting that more variants may still be discovered as new sequences become available. In human, we found 17 selenoproteins to have alternative splicing isoforms. Supplementary Figure S42 shows the number of splicing forms identified for each of them. TR1 alternative splicing is discussed in detail above and is the most abundant of all mammalian selenoproteins, with at least 10 splicing forms. Three selenoproteins (SelT, Dio1, and TR3) had each 4 identified splicing isoforms.

**Figure 8:**
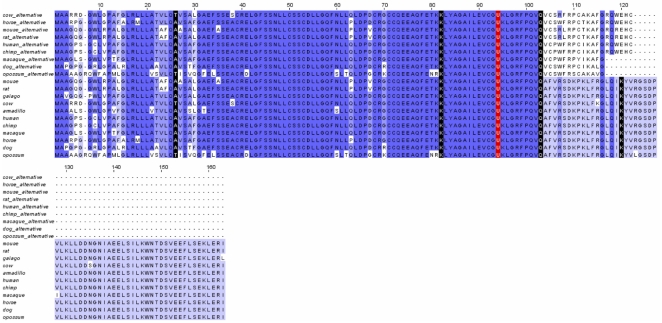
Multiple sequence alignment of Sep15 and a Sep15 alternative isoform. The last residue of each exon is marked in black and Sec in red. For human and mouse, ESTs support the presence of the isoform. For the other species shown, the protein sequences were predicted simulating skipping the 4th exon.

Of note was a splice variant in the Sep15 gene. In this isoform, the entire 4th exon was removed during processing of the pre-mRNA (Figure 8), resulting in a frameshift in the next exon and premature stop codon. However, there was evidence that this isoform may be expressed, primarily from the high number of ESTs supporting this variant. In humans, 41 ESTs from 26 libraries, representing a variety of tissues, supported this variant. In total, there were only 99 ESTs for this portion of Sep15, so ∼41% of ESTs for this region represented this variant. The variant was conserved in the mouse, where a single EST supported it. We expect that upcoming additional EST data for other mammals will confirm the presence of this isoform also in other species.

SelT is another selenoprotein for which we observed a previously unreported alternative isoform. This isoform contained an extra exon in the first intron. Multiple early stop codons and a frameshift were introduced by this new exon. We examined the new form for occurrence of an alternative translation start site, but no good candidates were found in this exon. Any transcript including this exon would thus code for a short protein which would be inactive. However, this form was supported by 13 human ESTs from several libraries and tissues. 15% (13 of 88) of the ESTs from these libraries supported this alternative form.

Two additional isoforms were identified in SelO, both of which were conserved in mice, rats, and humans. In the first, the penultimate exon was included in the transcript. This version was supported by 1 EST in humans, 10 ESTs in rats, and more than 30 ESTs in mice. The first full codon in the intron region was a stop codon in all three organisms, so there was high conservation along the entire protein, and they all terminated at the same location. The second newly identified isoform in SelO was similar to the previous, except that in this case the last intron was included in the mature transcript. Again, it was conserved in humans (1 EST), mice (16 ESTs), and rats (6 ESTs). This variant resulted in a frameshift in humans and rats, but not mice. Termination occurred in a different location in each of the three organisms with only mice still predicted to code for Sec.

We also identified a new isoform in GPx4. This isoform was conserved in mice and cows with 4 ESTs and 29 ESTs, respectively. In this isoform, the last intron was included in the mature transcript. No frameshift occurred in either of the animals; however, in mice a premature stop codon was introduced while the cow sequence was predicted to terminate as usual. An interesting point to consider was that termination in mice, although premature, was not far from the stop codon of the major form and was far from the Sec, so perhaps this isoform could be functional.

MsrB1 is another selenoprotein with alternative splicing forms. In most mammals, MsrB1 had 4 exons. In mice two different splicing forms were identified: a 4 exon version and a 5 exon version. The 5 exon version contained an extra intron in the 3′-UTR, so the protein sequence was unchanged. EST data suggested that the forms are equally expressed. Rats appeared to have only the 5 exon version, whereas other mammals appeared to have only the 4 exon version. We recently experimentally verified the occurrence of these forms, both of which result in the expression of the active MsrB1 [71].

Lastly, an interesting transcript variant was identified in SelS in humans. The major form of SelS contained 6 exons, whereas the alternative version had 7. Similar to MsrB1, the alternative splicing modified only the 3′-UTR. In the major form, the 3′-UTR and the last coding portion of the gene were in exon 6. In the alternative version, most of the 3′-UTR of the major form was spliced out and an entirely different 3′-UTR further downstream was included in the transcript. The unique feature of this splicing variant is that the alternative form did not contain a SECIS element. This would result in a non-functional truncated protein as Sec cannot be inserted. However, the detection of 13 human ESTs from numerous libraries and tissues suggested that this variant does in fact exist. A similar case was found for GPx3, although with less EST support. A variant in the 3′-UTR of GPx3 was identified in humans, featuring an extra intron in the 3′-UTR which corresponds almost exactly to the SECIS element.

## Discussion

Although much effort has been devoted to identifying selenoprotein genes and characterizing Sec insertion machinery, evolution of the vertebrate selenoproteome is incompletely understood. Important insights concerning the vertebrate selenoproteomes and individual selenoproteins have previously been provided based on the analyses of a limited number of sequenced genomes [25], [32]. In the present study, we scrutinized Sec- and Cys-containing homologs of known eukaryotic selenoprotein families in 44 vertebrate genomes, including 34 mammals. The number of organisms examined in this study should be considered sufficiently deep to identify the main themes in selenoprotein evolution. Although ongoing vertebrate genome projects will undoubtedly uncover various clade-specific features and allow refinements, the general features of the utilization and evolution of Se should not change. Across all vertebrates, a set of 45 selenoproteins was identified, with at most 38 represented in a single organism (zebrafish). 27 selenoproteins were found to be unique to vertebrates. 20 of them were generated through duplication of an existing selenoprotein in some vertebrate lineage, while 6 of them were part of the predicted ancestral selenoproteome. This implies that these latter 6 proteins (GPx2, Dio2, Dio3, SelI, SelPb, Fep15) were generated at the root of vertebrates. Individual mammalian selenoproteomes consist of 24/25 selenoproteins, from a set of 28. Our results reinforce the idea that the mammalian selenoproteome has remained relatively stable. However, a number of evolutionary events that changed its composition were observed (Figure 1): GPx6 and SelV were originated, SelPb was lost, SPS2b appeared and replaced SPS2a, SelV was lost in gorilla, and selenoproteins SelU1, Dio3 and GPx6 were converted to their Cys-containing forms in major or minor mammalian lineages.

**Figure 9:**
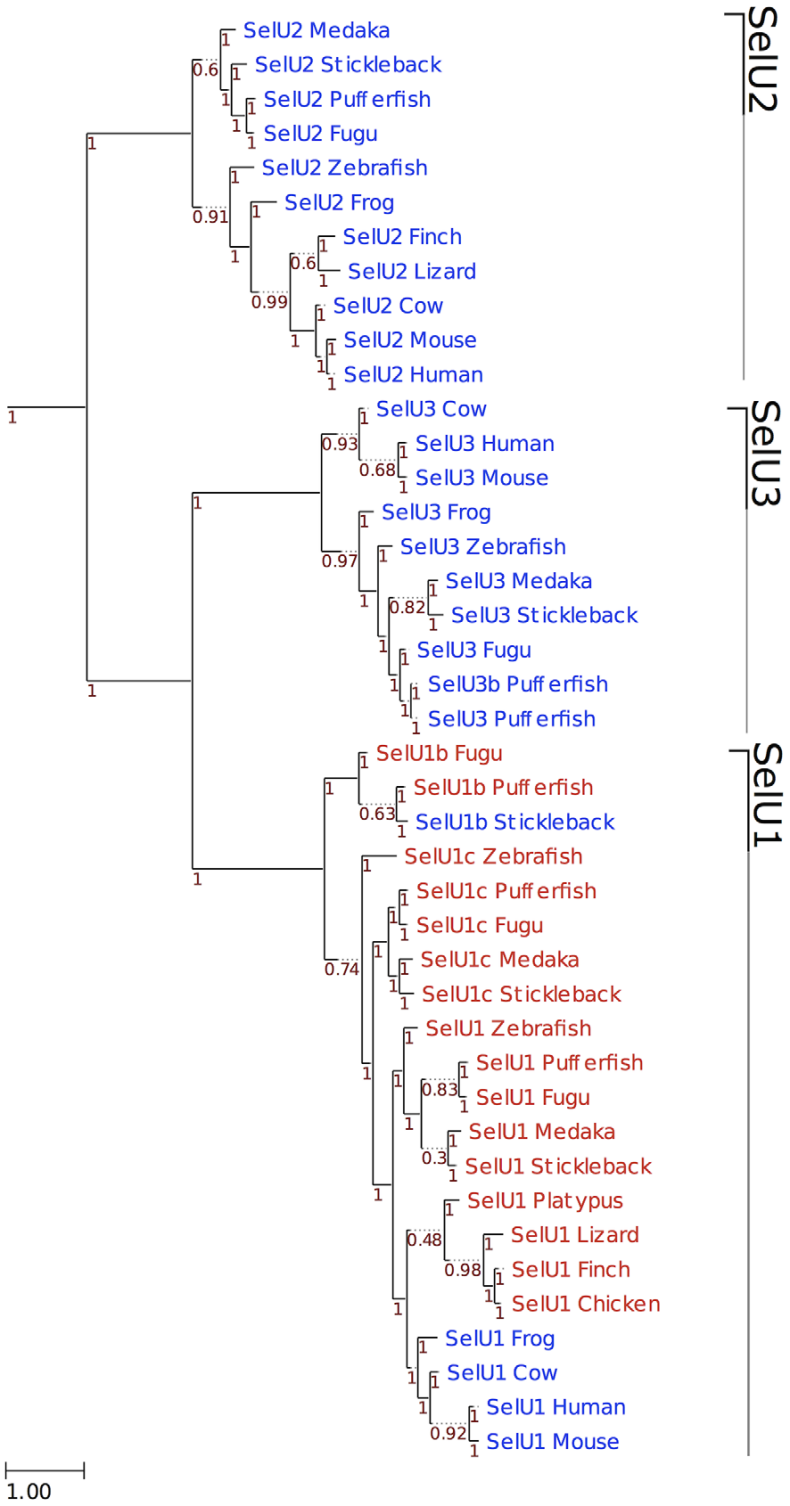
Phylogeny of SelU family in vertebrates. ML tree computed using the JTT substitution model. Sec-containing proteins are shown in red, whereas the Cys-containing homologs are shown in blue. At the bottom left, the distance scale in substitutions per position is shown. Branch support is shown along the tree in red.

The ancestral vertebrate selenoproteome was uncertain, as fish had many selenoproteins resulting of genome duplication and gene duplication within bony fishes [25]. Previously, it has been suggested that the ancestral vertebrate selenoproteome consists of 31 selenoproteins: Dio1-3, GPx1-4, SelH, SelI, SelJ, SelK, SelL, SelM, SelN, SelO, SelP, SelPb, MsrB1, SelS, SelT1, SelU1-3, SelV, SelW1, SelW2a, Sep15, SPS2, TR1, TR3 and TGR [32]. In this study, we examined the occurrence of these selenoproteins in additional mammals and newly sequenced organisms which are important outgroups for understanding the evolution of different vertebrate clades (such as platypus and opossum). Particularly, we used both genomic and Trace databases for reconstruction of the selenoproteome of the phylogenetically oldest group of living jawed vertebrates, the elephant sharks. As a result, a number of new aspects were uncovered: (i) Fep15, which was previously thought to evolve in bony fish, was detected as a selenoprotein in elephant shark and as a Cys homolog in frog, and therefore should be viewed as part of the ancestral selenoproteome; (ii) TGR was found exclusively in tetrapods; (iii) SelV was found exclusively in placental mammals; (iv) phylogenetic analysis of Sec- and Cys-containing forms of the SelU family suggested that all Sec-containing SelU sequences belong to the SelU1 group (Figure 9). Mammals contain three Cys-containing SelU proteins (SelU1-3), whereas some fish (such as fugu and pufferfish) have three Sec-containing SelU proteins. It was previously thought that the three Cys-containing SelU proteins in mammals evolved from the three Sec-containing SelU sequences in fish. In this study, we could not find evidence that supports an early Sec-to-Cys conversion event for SelU2 and SelU3 proteins. Thus, the revised ancestral selenoproteome consists of the following 28 selenoproteins: GPx1-4, TR1, TR3, Dio1-3, SelH, SelI, SelJ, SelK, SelL, SelM, SelN, SelO, SelP, SelPb, MsrB1, SelS, SelT1, SelU1, SelW1, SelW2, Sep15, Fep15 and SPS2a (Figure 1).

Our analysis also uncovered the changes in the ancestral selenoproteome across vertebrates. Bony fishes were confirmed to be a lineage featuring several duplications. We predicted 14 in total: Dio3b, GPx1b, GPx3b, GPx4b, GPx4b2, MsrB1b, SelJ2, SelO2, SelT2, SelT1b, SelU1b, SelU1c, SelW2b and SelW2c. Interestingly, we found 3 selenoprotein duplications specifically in zebrafish (SelO2, SelT1b, SelW2b). As more fish sequences become available, further analysis will tell how common recent and lineage specific these duplications are. We also predicted all Sec to Cys conversions along the vertebrate tree, finding 12 such events. Particularly interesting was the case of GPx6, which was converted to the Cys form in at least 3 mammalian lineages independently. One of these events occurred in marmoset, a unique case among all 9 primates investigated. Notably, we observed proteins that do not bear Sec in any organism, but were generated through duplication of selenoprotein genes. In these cases (GPx5, GPx4b2, Rdx12), the conversion of the Sec TGA to a Cys codon must have happened early after the duplication, probably before the duplicated gene haplotype became fixed.

Comparative analyses of nucleotide and protein sequences of vertebrate selenoproteins revealed complex evolutionary histories in several families. First, SelV most likely arose from duplication of SelW in the ancestor of placental mammals, followed by addition of N-terminal sequences whose function is unclear as well as a deletion of a substantial portion of the 3′-UTR. Second, our analysis of GPx1-8 families highlighted three evolutionarily related groups: GPx1/GPx2, GPx3/GPx5/GPx6 and GPx4/GPx7/GPx8 (Figure 4). GPx4 appeared to be the most ancient GPx, whereas GPx5 and GPx6 were the most recently evolved GPx forms. Third, phylogenetic analyses of TR1 and TGR showed that these proteins evolved by gene duplication from an ancestral TR protein that is similar to a fish Grx-containing TR1. TR1 then suffered the loss of the Grx domain, except in some organisms (such as humans), which still retain it as an alternative isoform, whereas TGR acquired a new function (related to spermatogenesis) during evolution.

One of the most important features of selenoprotein genes is the SECIS element, which is located in the 3′-UTR. The most critical parts of the SECIS element are the SECIS core (located within the stem) and the two conserved nucleotides (of unknown function) in the apical loop. Within every examined SECIS element the GA/GA, paired non-canonically, were essential and conserved. Additionally, the two unpaired nucleotides within the apical loop are typically adenines; however, SECIS elements of SelM and SelO evolved cytosines in these positions specifically within mammals.

We also examined additional features of mammalian selenoprotein genes. First, we identified interesting pseudogenes of GPx1 and SelW. These genes showed patterns of high conservation, including Ka/Ks values that may suggest active selective pressure. However, other characteristics indicate that they cannot code for functional proteins. Lack of EST data suggests that they are not (at least widely) expressed. It has been reported that quite few pseudogenes can go through the process of transcription in a tissue-specific manner [72]. They may play a role in regulation and expression of homologous genes or other genes [73]–[77]. Thus, it is possible that these pseudogenes may still be expressed in narrow niches to regulate the mRNA stability of SelW or for other functions.

Second, we identified a number of alternative splicing forms for the majority of mammalian selenoproteins in different organisms that had not been previously reported. This data may provide new insights into the post-transcriptional regulation of selenoprotein genes in mammals. Many of the alternative transcripts reported here also possess features that suggest they cannot code a functional protein, particularly due to the presence of frameshifts. The evidence of transcription and the conservation in multiple species suggests nevertheless some biological role. The alternative splicing forms that appeared to be conserved in multiple species (such as Sep15, SelT, SelO, GPx4 and MsrB1) represent top candidates for further experimental investigation.

Concluding, in this work we carried out comprehensive analyses of selenoproteomes in sequenced vertebrates to better define the roles of selenium and selenoproteins in these organisms. Our data provide a wealth of information on the composition and evolution of vertebrate and mammalian selenoproteomes. We revised the ancestral vertebrate selenoproteome and traced its evolution across all sequenced vertebrate lineages. This provided new insights into the evolution of selenoprotein families, in particular of glutathione peroxidases and thioredoxin reductases. Furthermore, we performed comparative analyses of gene structures and SECIS elements in mammalian selenoproteins, identified novel alternative splicing forms, and reported unusually conserved selenoprotein pseudogenes.

## Materials and Methods

### Genomic sequences and resources

All vertebrate genomes with significant sequence coverage from the current Entrez Genome Project at NCBI were used in this study (a total of 44 organisms). Additional databases that are related to each organism, such as Trace Archive database, EST database, non-redundant protein and nucleotide databases, were also retrieved from NCBI.

### Identification and analyses of Sec/Cys-containing homologs, UTRs, SECIS elements, alternative splicing forms

We used several representative sequences of all known eukaryotic selenoproteins that were reported in previous studies as queries to search for Cys- and Sec-containing homologs in mammals and other vertebrates via BLAST with default parameters [78], [79]. The automated predictions by program Selenoprofiles 2 [80] were also examined. For selenoprotein superfamilies (those including many genes sharing high homology, such as GPx and TR), the subfamilies were assigned based on the phylogenetic analysis. Gene losses were trusted only when observed in multiple species, or when both a high coverage genome assembly and abundant ESTs were available. The set of vertebrate-specific selenoproteins were determined by searching all ancestral selenoprotein sequences in the genomes of two chordate outgroups: amphioxus and sea squirt. For proteins not found in these species, additional searches were also performed in all non-vertebrate animal sequences. For selenoprotein superfamilies, a phylogenetic analysis of the non-vertebrate candidate sequences along with the vertebrate members was performed to assign subfamilies. UTRs were determined using EST data, and multiple sequence alignments were used to predict UTRs in animals with inadequate EST data. SECIS elements were predicted using SECISearch program [9]. Instances of alternative splicing were identified using BLAST search against EST data.

### Multiple sequence alignment and phylogenetic analysis

Multiple sequence alignments were performed using ClustalW [81] and Mafft [82]. Phylogenetic reconstruction was performed as follows. ML trees were reconstructed using the best-fitting evolutionary model (BestML). To select the evolutionary model best fitting each protein family, a phylogenetic tree was reconstructed using a Neighbour Joining (NJ) approach as implemented in BioNJ [83]. The likelihood of this topology was computed, allowing branch-length optimization, using seven different models (JTT, LG, WAG, Blosum62, MtREV, VT and Dayhoff), as implemented in PhyML version 3.0 [84]. The two evolutionary models best fitting the data were determined by comparing the likelihood of the used models according to the AIC criterion [85]. Then, ML trees were derived using these two models with the default tree topology search method NNI (Nearest Neighbor Interchange). A similar approach based on NJ topologies to select the best-fitting model for a subsequent ML analysis has been shown previously to be highly accurate [86]. Branch support was computed using an aLRT (approximate likelihood ratio test) parametric test based on a chi-square distribution, as implemented in PhyML Finally, multiple sequence alignments were visualized with Jalview [87], and phylogenies with Ete2 [88].

## Supporting Information

Figure S1 **Multiple sequence alignment of Dio1.** The approximate positions of introns are marked in black and the Sec is shown in red.
(TIFF)

Figure S2 **Multiple sequence alignment of Dio2.** Residues are marked as in Supplementary Figure S1.
(TIFF)

Figure S3 **Multiple sequence alignment of Dio3.** Residues are marked as in Supplementary Figure S1.
(TIFF)

Figure S4 **Multiple sequence alignment of GPx1.** Residues are marked as in Supplementary Figure S1.
(TIFF)

Figure S5 **Multiple sequence alignment of GPx2.** Residues are marked as in Supplementary Figure S1.
(TIFF)

Figure S6 **Multiple sequence alignment of GPx3.** Residues are marked as in Supplementary Figure S1.
(TIFF)

Figure S7 **Multiple sequence alignment of GPx4.** Residues are marked as in Supplementary Figure S1.
(TIFF)

Figure S8 **Multiple sequence alignment of GPx6.** Residues are marked as in Supplementary Figure S1.
(TIFF)

Figure S9 **Multiple sequence alignment of MsrB1.** Residues are marked as in Supplementary Figure S1.
(TIFF)

Figure S10 **Multiple sequence alignment of SelH.** Residues are marked as in Supplementary Figure S1.
(TIFF)

Figure S11 **Multiple sequence alignment of SelI.** Residues are marked as in Supplementary Figure S1.
(TIFF)

Figure S12 **Multiple sequence alignment of SelK.** Residues are marked as in Supplementary Figure S1.
(TIFF)

Figure S13 **Multiple sequence alignment of SelM.** Residues are marked as in Supplementary Figure S1.
(TIFF)

Figure S14 **Multiple sequence alignment of SelN.** Residues are marked as in Supplementary Figure S1.
(TIFF)

Figure S15 **Multiple sequence alignment of SelO.** Residues are marked as in Supplementary Figure S1.
(TIFF)

Figure S16 **Multiple sequence alignment of SelP.** Residues are marked as in Supplementary Figure S1. Note that there are multiple Sec in each protein.
(TIFF)

Figure S17 **Multiple sequence alignment of SelPb.** Residues are marked as in Supplementary Figure S1.
(TIFF)

Figure S18 **Multiple sequence alignment of SelS.** Residues are marked as in Supplementary Figure S1.
(TIFF)

Figure S19 **Multiple sequence alignment of SelT.** Residues are marked as in Supplementary Figure S1.
(TIFF)

Figure S20 **Multiple sequence alignment of SelV.** Residues are marked as in Supplementary Figure S1.
(TIFF)

Figure S21 **Multiple sequence alignment of SelW and SelW2 proteins.** Residues are marked as in Supplementary Figure S1.
(TIFF)

Figure S22 **Multiple sequence alignment of Sep15.** Residues are marked as in Supplementary Figure S1.
(TIFF)

Figure S23 **Multiple sequence alignment of SPS2.** Residues are marked as in Supplementary Figure S1. Note that in more ancient mammals and vertebrates the SPS2 gene is a multi-exon gene.
(TIFF)

Figure S24 **Multiple sequence Alignment of TGR.** Residues are marked as in Supplementary Figure S1.
(TIFF)

Figure S25 **Multiple sequence alignment of TR1.** Residues are marked as in Supplementary Figure S1.
(TIFF)

Figure S26 **Multiple sequence alignment of TR3.** Residues are marked as in Supplementary Figure S1.
(TIFF)

Figure S27 **Multiple sequence alignment of SelL.** The Sec is shown in red.
(TIFF)

Figure S28 **Multiples sequence alignment of Fep15.** Residues are marked as in Supplementary Figure S1.
(TIFF)

Figure S29 **Multiple sequence alignment of SelJ.** Residues are marked as in Supplementary Figure S1.
(TIFF)

Figure S30 **Multiple sequence alignment of SelU1.** Residues are marked as in Supplementary Figure S1.
(TIFF)

Figure S31 **Multiple sequence alignment of mammalian SelT1 coding sequences.** The last residue of each exon is marked in black, and the codon corresponding to the Sec is shown in red.
(TIFF)

Figure S32 **SECIS in SPS2a and SPSb.** Multiple sequence alignment of opossum SPS2a and SPS2b, platypus SPS2a, and human SPS2b SECIS elements. Critical portions are marked in red.
(TIFF)

Figure S33 **Multiple sequence alignment of SelW and SelV 3′-UTRs.** Critical portions of the SECIS elements are marked in red. The last nucleotide of exon 5 is marked in black.
(TIFF)

Figure S34 **Phylogenetic tree of SelW1, SelW2 and SelV proteins.** ML phylogenetic tree of SelW1, SelW2 and SelV protein sequences computed using the WAG substitution model. The branch support for each node (computed as described in the methods) is shown in red. The bar at the bottom left shows the scale in substitutions per position. The Rdx12 gene was found in all tetrapodes but only frog, mouse and human were included in the phylogenetic tree. In contrast all SelW2 detected were included: this gene is missing in all tetrapodes apart from frog. SelW1 is missing from bony fishes apart from zebrafish. SelV was detected in all placentals except gorilla but only rat, cow and human were included. Note that while the SelV-SelW1 duplication is clear and well supported, the rest of the tree is more confused. Nonetheless SelW2c, SelW2b and Rdx12 appear to have been generated by independent duplications.
(TIFF)

Figure S35 **Multiple sequence alignment of TGR, zebrafish TR1, and GRx-containing TR1.** Residues are marked as in Figure S1. Note positions where zebrafish TR1 and TGR match, but are different than GRx-containing TR1 (i.e., positions 43, 142, 143, 149, 150, 324, etc.).
(TIFF)

Figure S36 **Multiple sequence alignment of extended Dio2 sequences.** The last residue of each exon is marked in black and the Sec residues in red. The second Sec, residue 269, is the stop codon or potential second Sec.
(TIFF)

Figure S37 **Multiple sequence alignment of SelI and its sequence homologs.** Homologs are labeled with the annotated name. Some sequences not annotated as CHPT1 or CEPT1 were also included, as they contain the same domain. Important residues in the active sites are marked in red. The last residue of each side of all predicted transmembrane regions are marked in green. Selenocysteines are marked in red. The cysteines emerged specifically in SelI proteins are also marked: the best candidate (near the catalytic side, on the same side of membrane of Sec) is marked in orange, while the other 2 cysteines are marked in yellow.
(TIFF)

Figure S38 **Phylogenetic tree of SelI and its sequence homologs.** ML tree computed using WAG model and the alignment shown in supplementary figure S37. The bar at the bottom left shows the scale in substitutions per position, while the branch support for each node is shown in red.
(TIFF)

Figure S39 **5′-UTR lengths.** 5′-UTR lengths are shown for various mammals. No length means there was insufficient EST data to define the 5′-UTR.
(TIFF)

Figure S40 **3′-UTR lengths.** 3′-UTR lengths are reported for various mammals. No length means there was insufficient EST data to define the 3′-UTR.
(TIFF)

Figure S41 **Number of ESTs versus number of splicing forms.** Left axis corresponds to the number of available ESTs (millions) and the right axis to the number of identified splicing forms identified in the listed animals.
(TIFF)

Figure S42 **Splicing forms per selenoprotein.** The numbers of splicing forms for each of 25 selenoproteins in mammals are shown.
(TIFF)

Table S1 **Scientific names of species investigated in this study.**
(DOCX)
