# Supplementary figures and images for: Composition and Evolution of the Vertebrate and Mammalian Selenoproteomes
Source: PLoS One. 2012 Mar 30;7(3):e33066. doi: 10.1371/journal.pone.0033066 (PMC3316567; doi:10.1371/journal.pone.0033066)

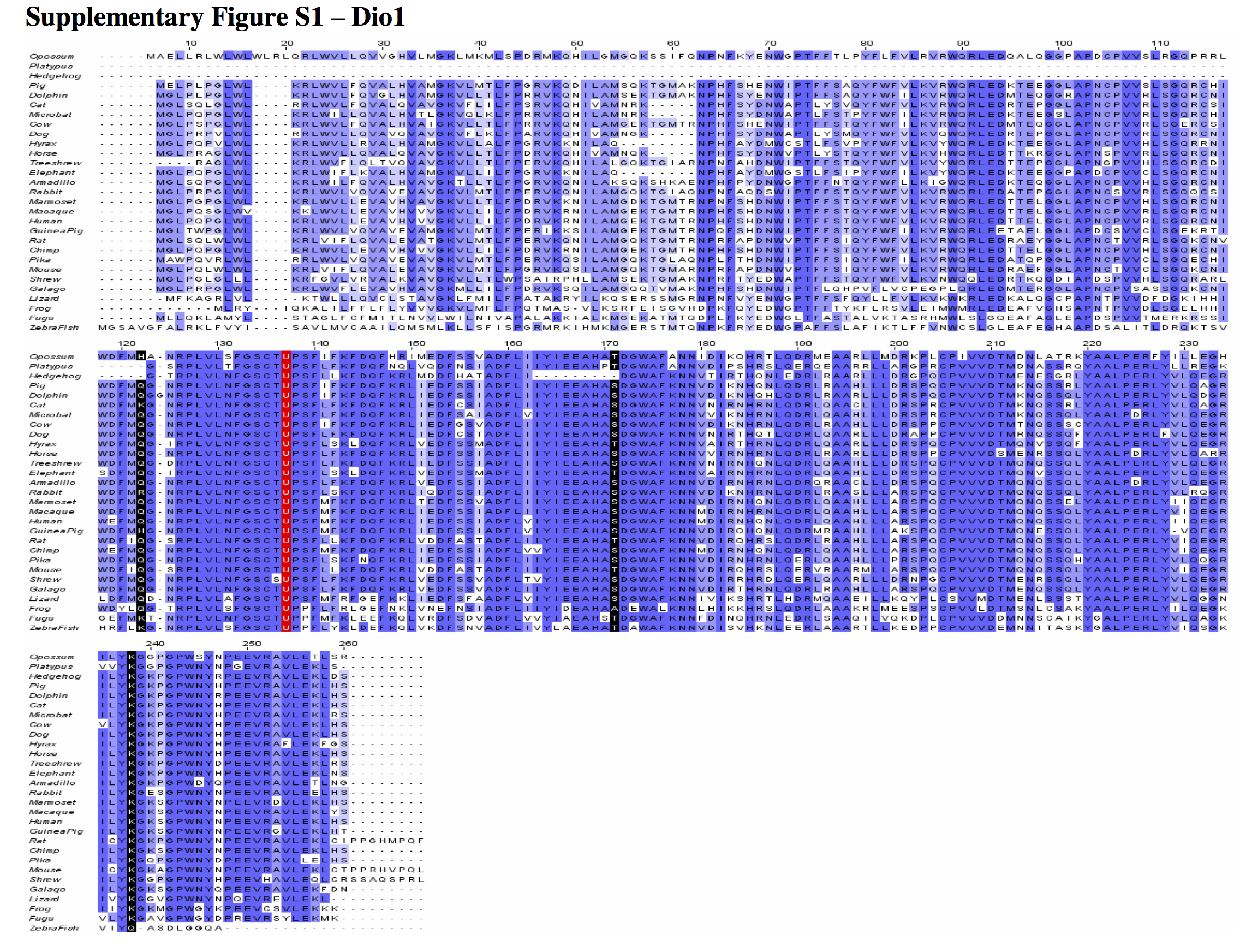

Supplement: Figure S1 — Multiple sequence alignment of Dio1. The approximate positions of introns are marked in black and the Sec is shown in red. (TIFF) [file pone.0033066.s001.tif]

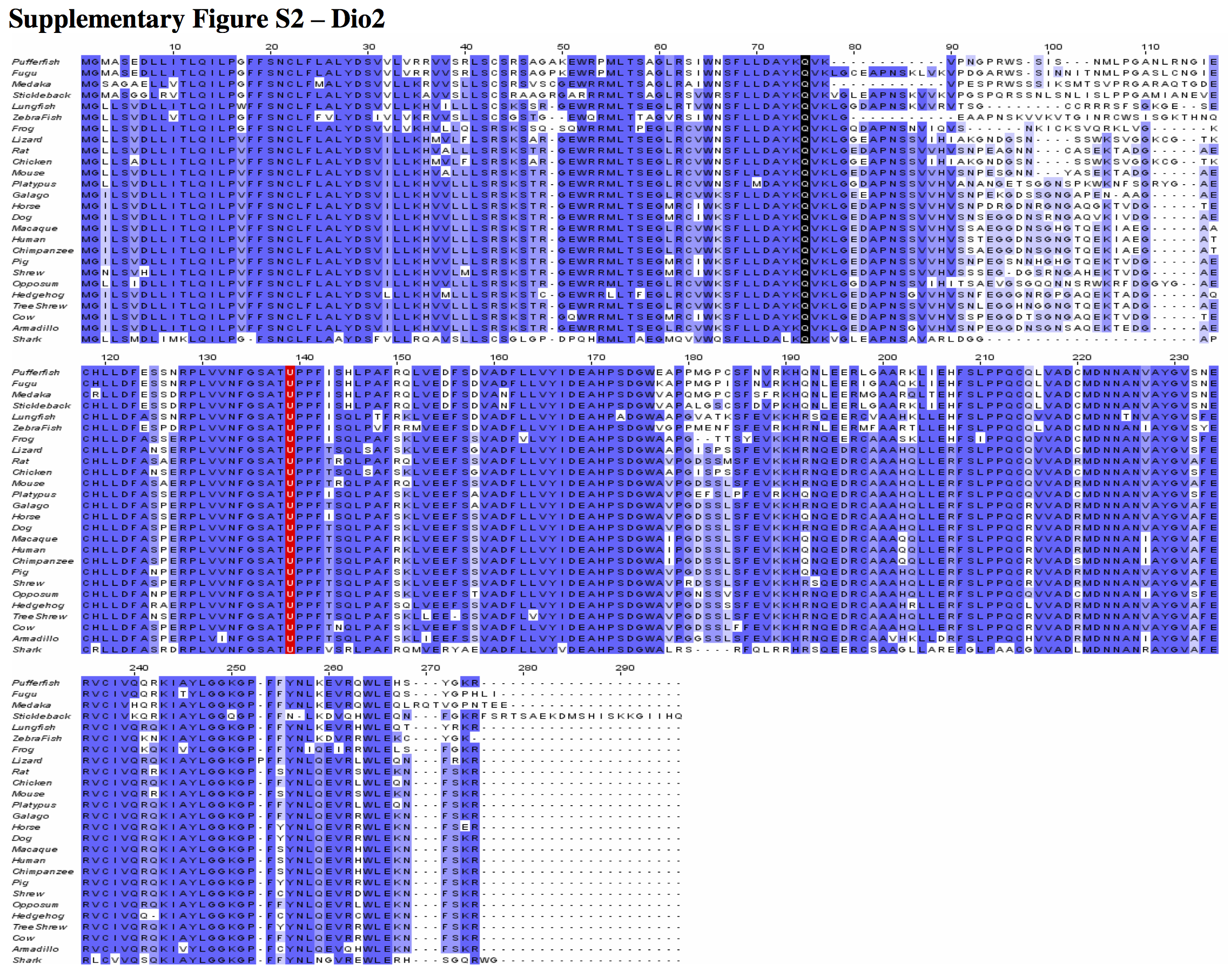

Supplement: Figure S2 — Multiple sequence alignment of Dio2. Residues are marked as in Supplementary Figure S1. (TIFF) [file pone.0033066.s002.tif]

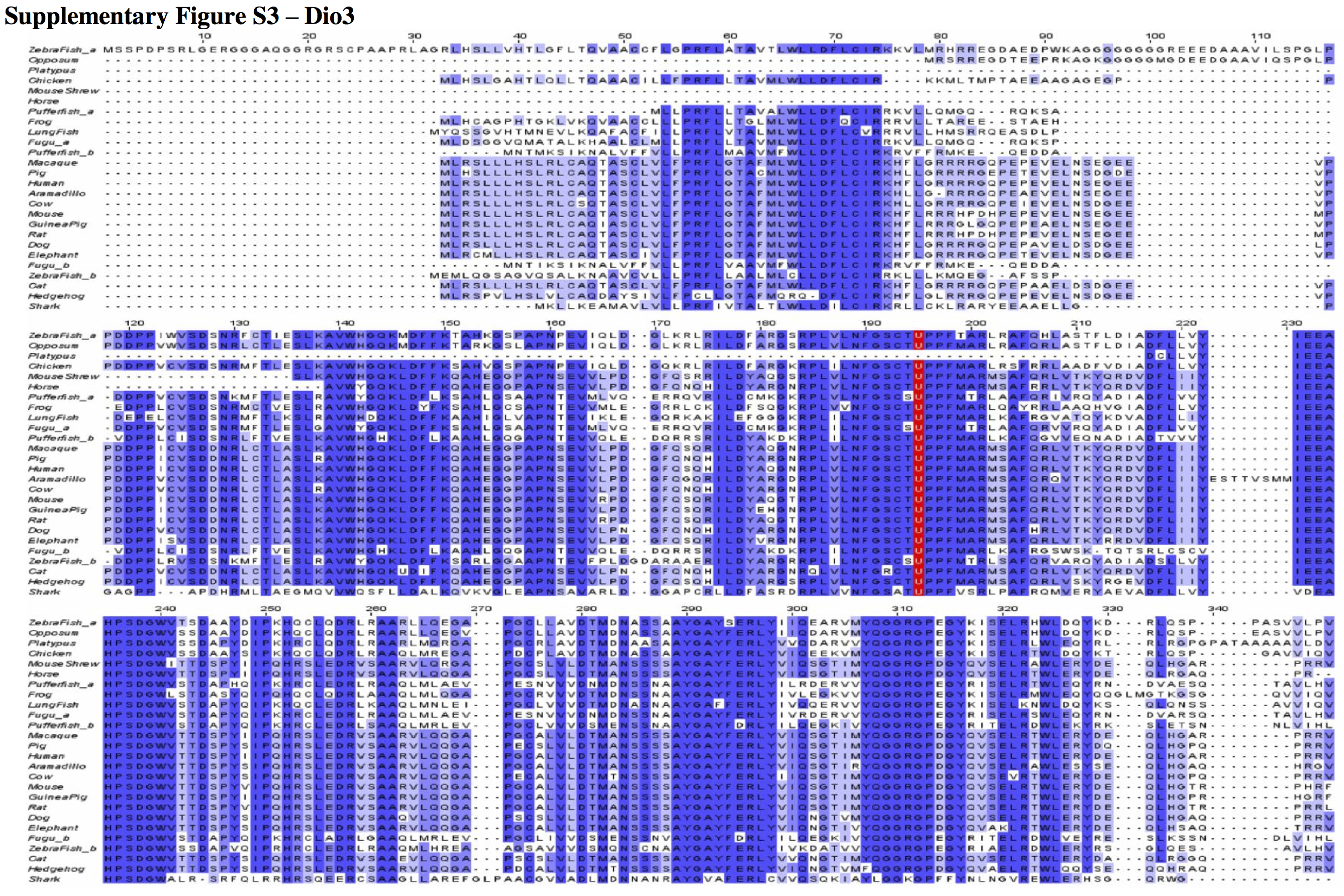

Supplement: Figure S3 — Multiple sequence alignment of Dio3. Residues are marked as in Supplementary Figure S1. (TIFF) [file pone.0033066.s003.tif]

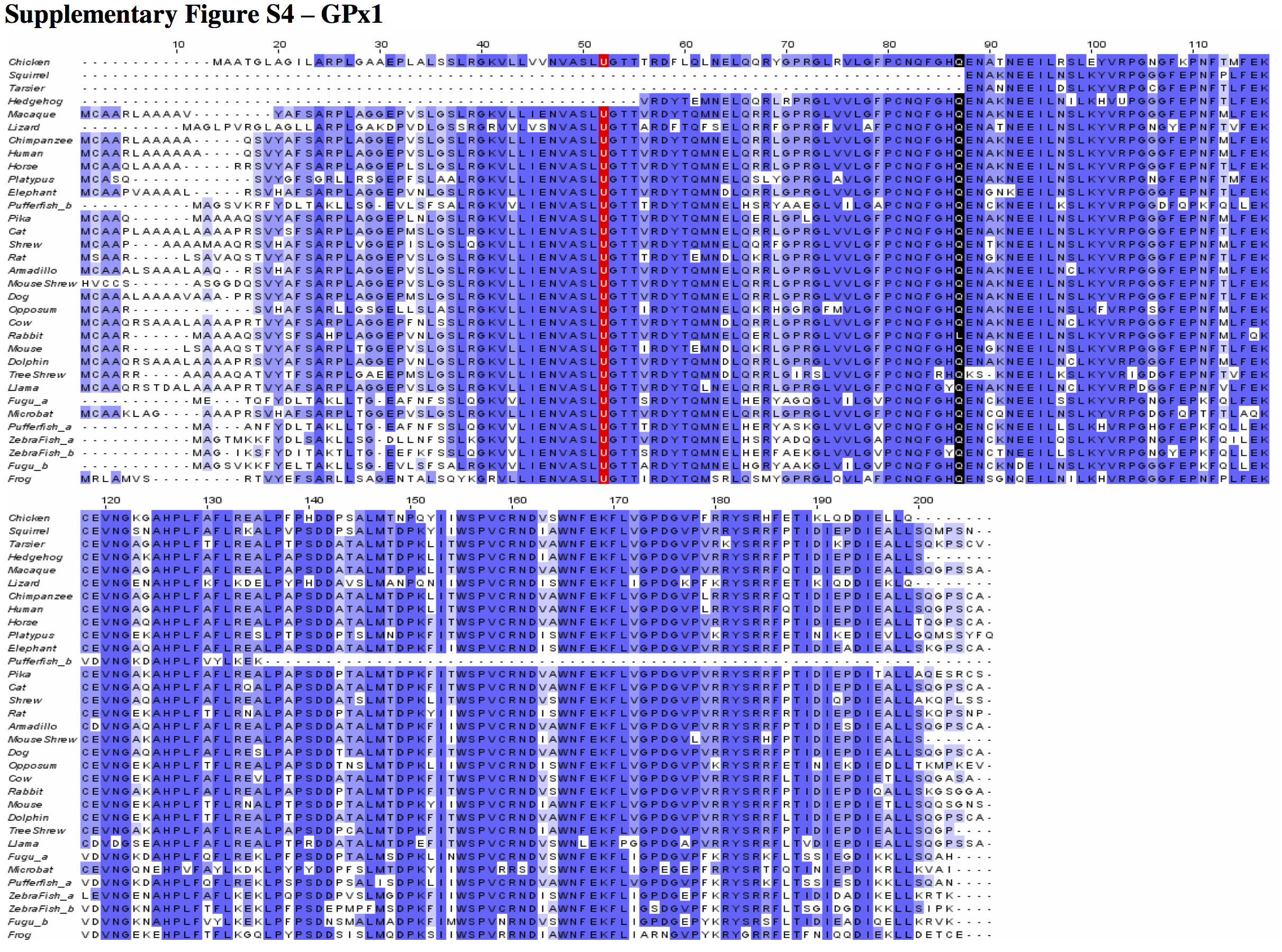

Supplement: Figure S4 — Multiple sequence alignment of GPx1. Residues are marked as in Supplementary Figure S1. (TIFF) [file pone.0033066.s004.tif]

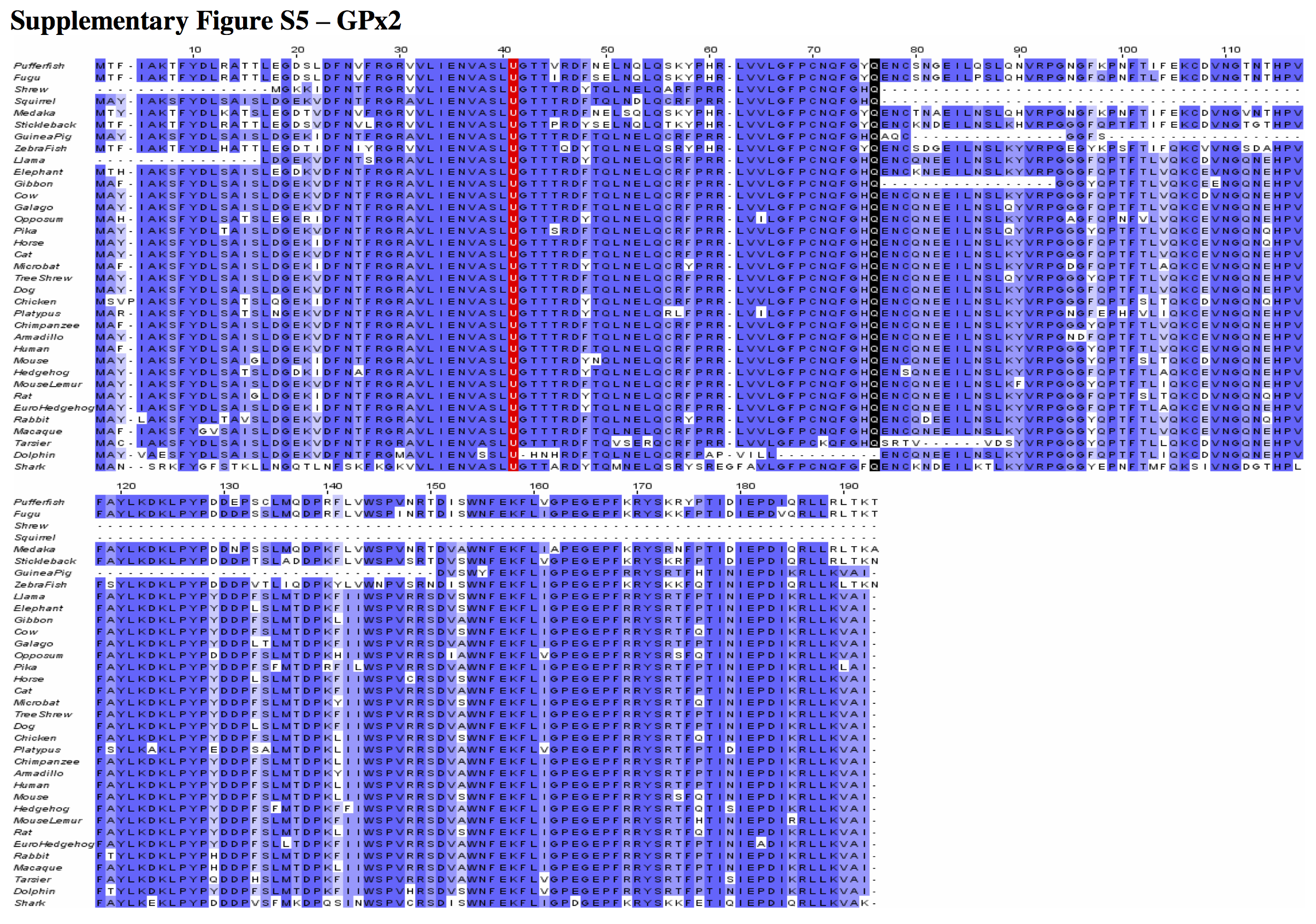

Supplement: Figure S5 — Multiple sequence alignment of GPx2. Residues are marked as in Supplementary Figure S1. (TIFF) [file pone.0033066.s005.tif]

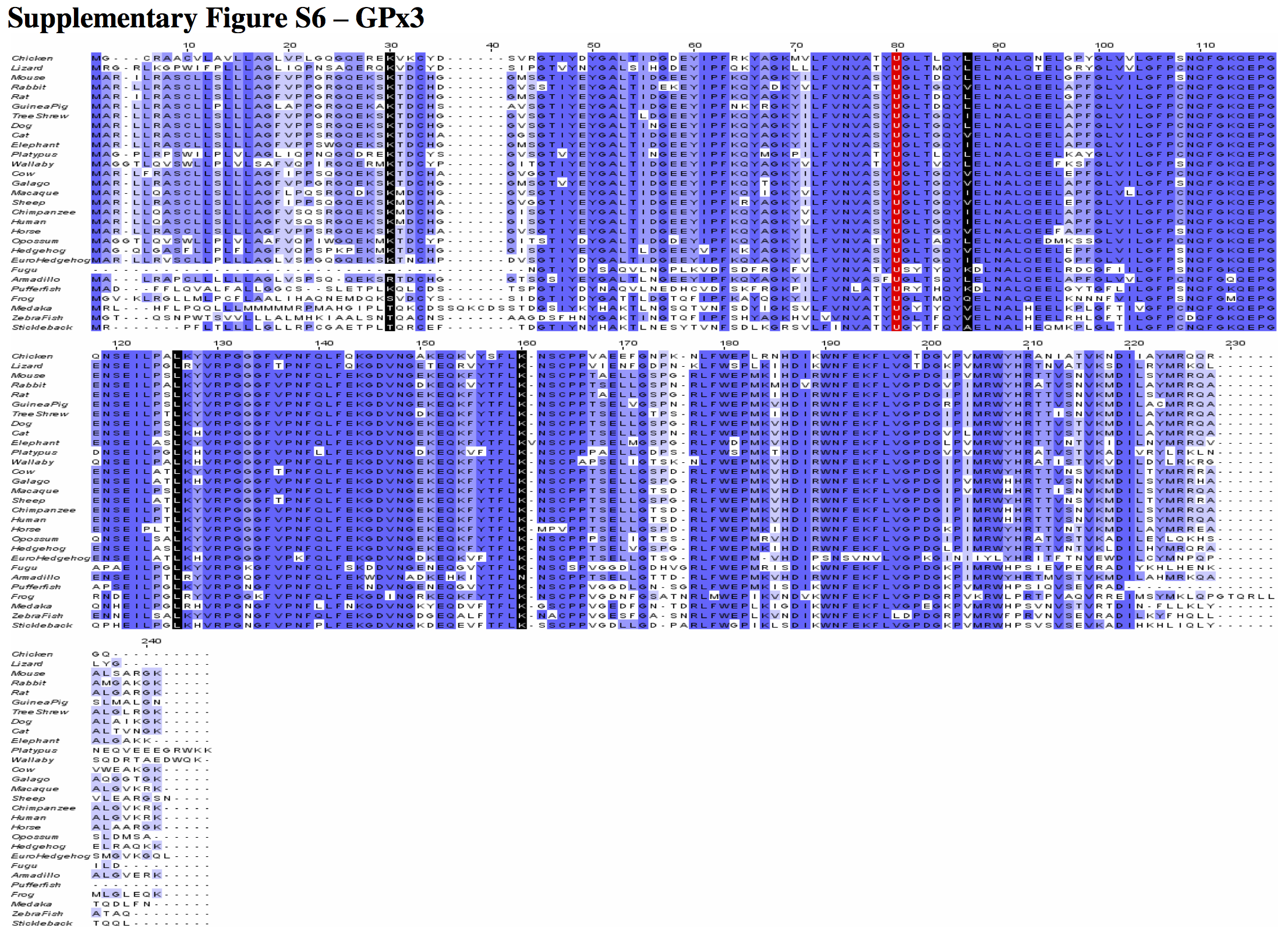

Supplement: Figure S6 — Multiple sequence alignment of GPx3. Residues are marked as in Supplementary Figure S1. (TIFF) [file pone.0033066.s006.tif]

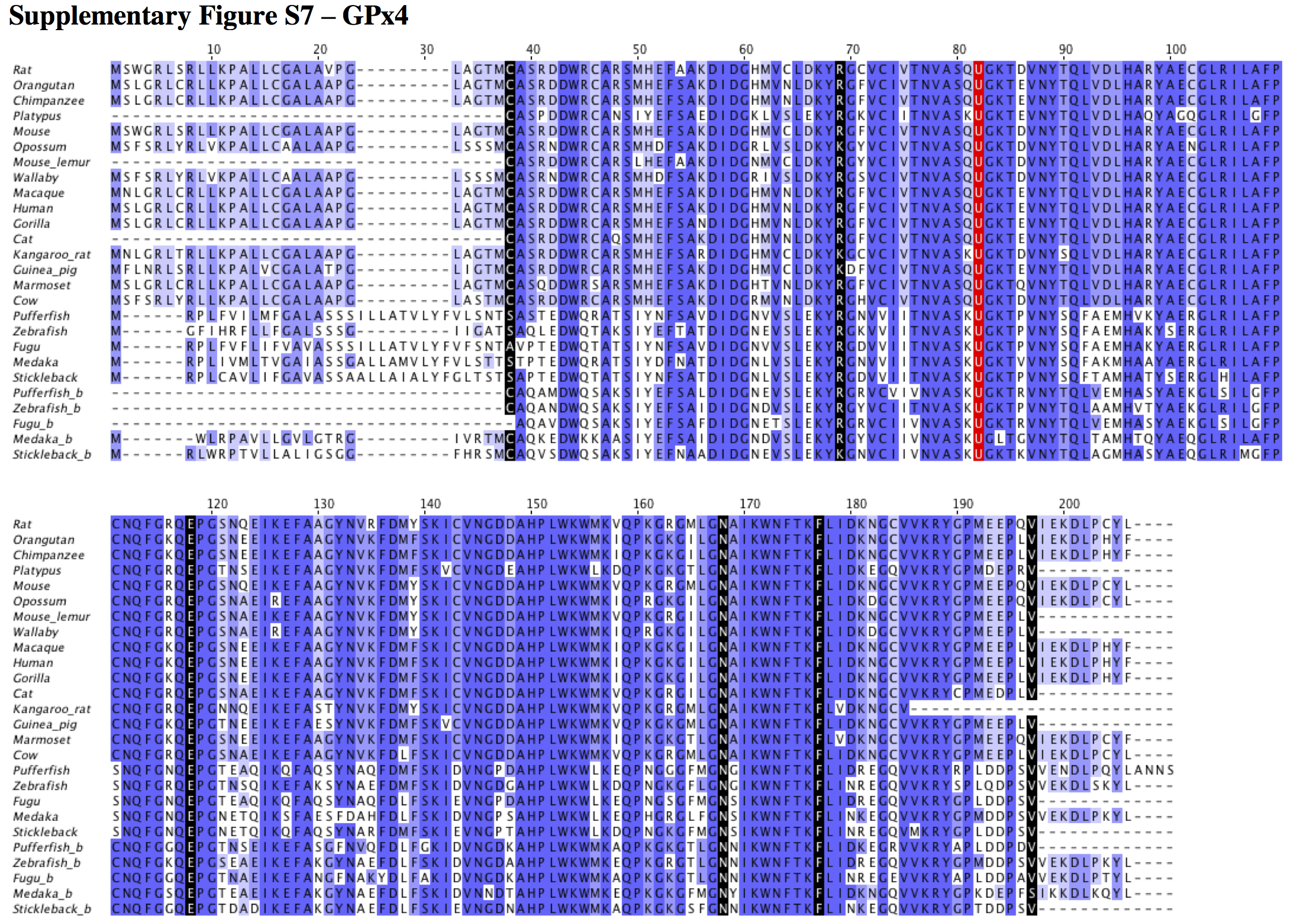

Supplement: Figure S7 — Multiple sequence alignment of GPx4. Residues are marked as in Supplementary Figure S1. (TIFF) [file pone.0033066.s007.tif]

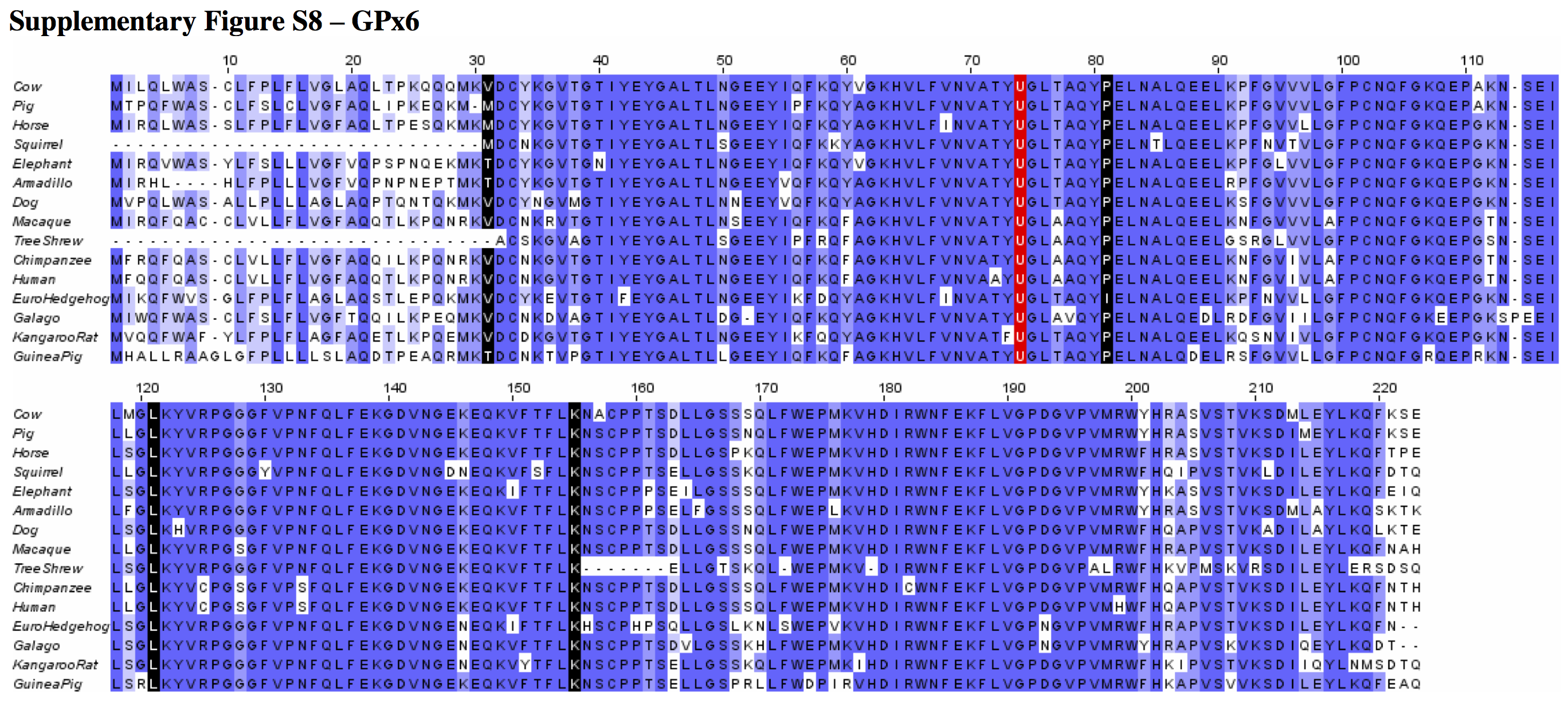

Supplement: Figure S8 — Multiple sequence alignment of GPx6. Residues are marked as in Supplementary Figure S1. (TIFF) [file pone.0033066.s008.tif]

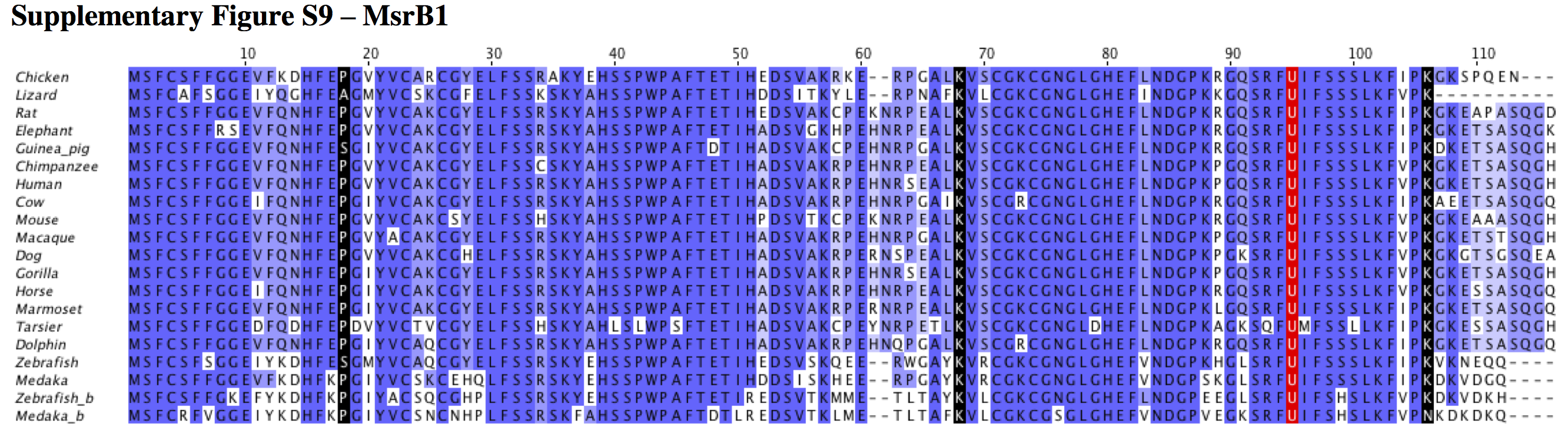

Supplement: Figure S9 — Multiple sequence alignment of MsrB1. Residues are marked as in Supplementary Figure S1. (TIFF) [file pone.0033066.s009.tif]

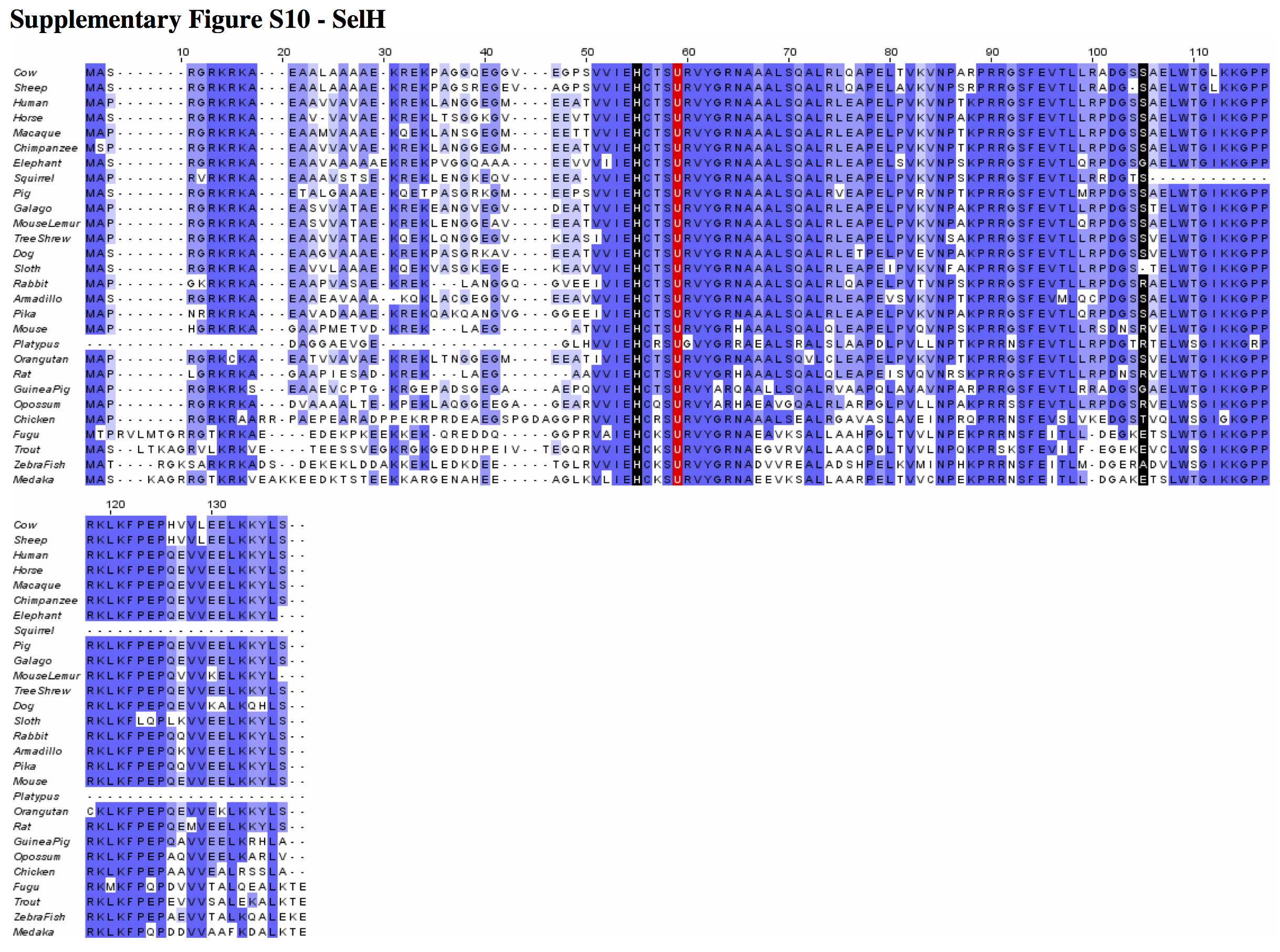

Supplement: Figure S10 — Multiple sequence alignment of SelH. Residues are marked as in Supplementary Figure S1. (TIFF) [file pone.0033066.s010.tif]

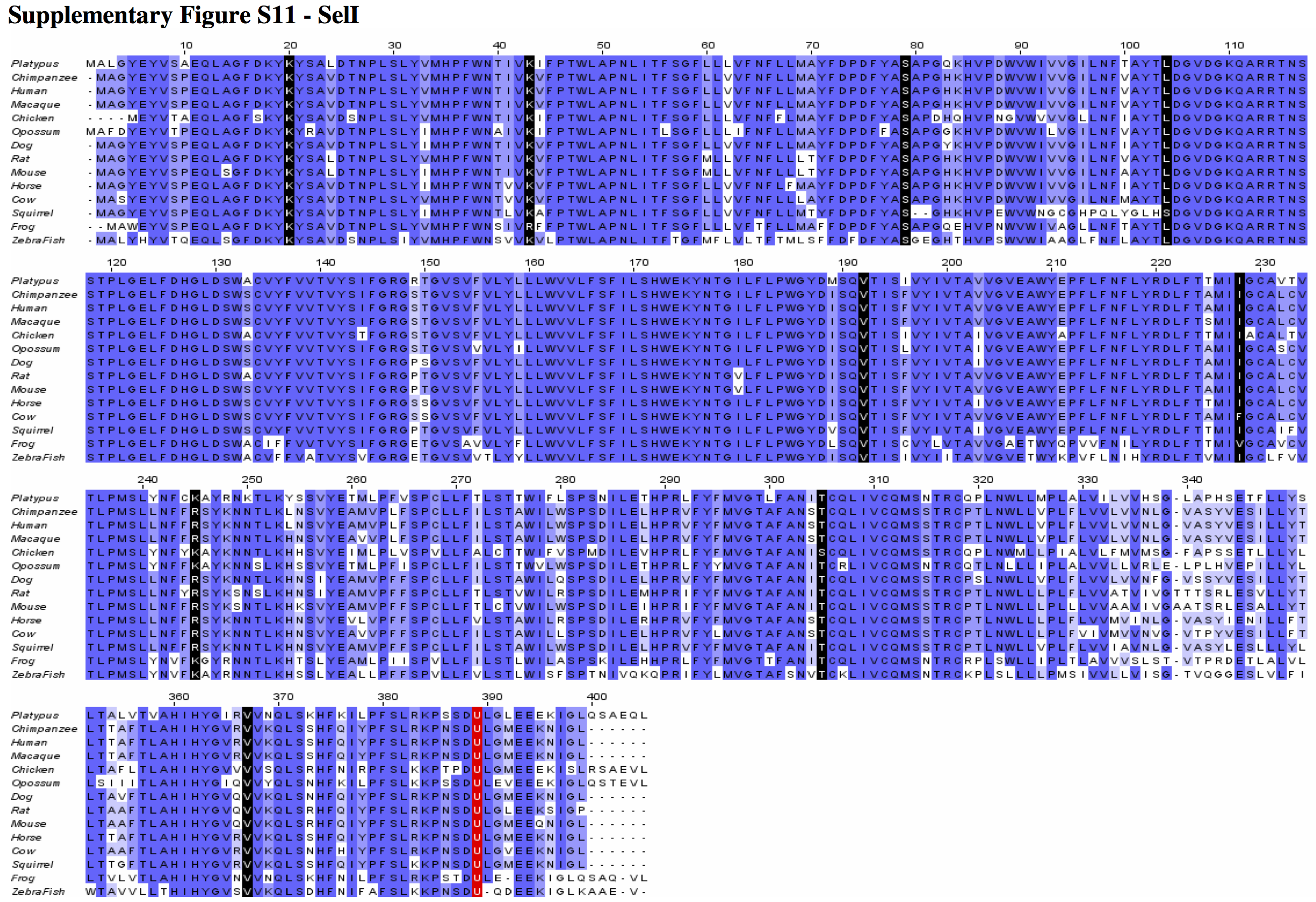

Supplement: Figure S11 — Multiple sequence alignment of SelI. Residues are marked as in Supplementary Figure S1. (TIFF) [file pone.0033066.s011.tif]

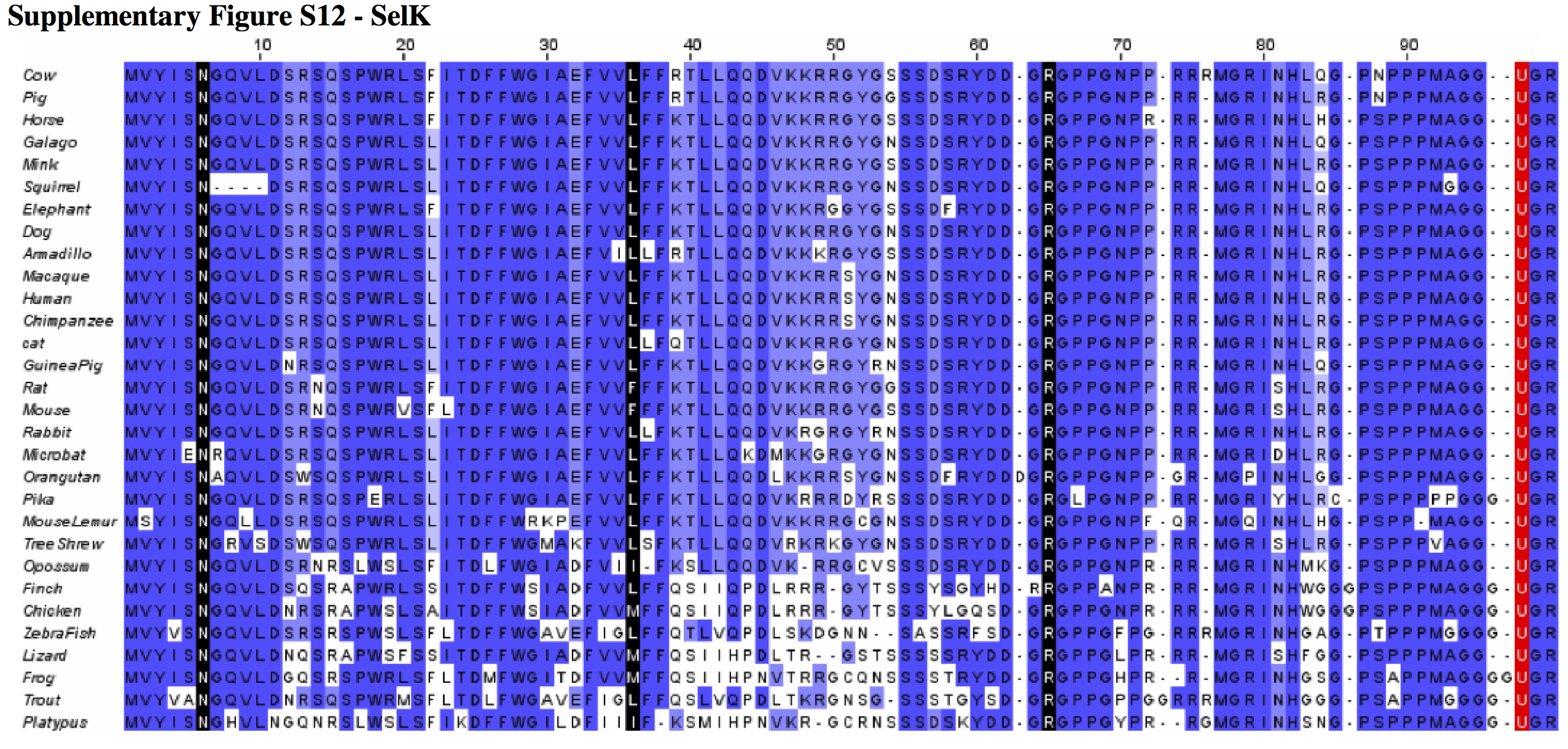

Supplement: Figure S12 — Multiple sequence alignment of SelK. Residues are marked as in Supplementary Figure S1. (TIFF) [file pone.0033066.s012.tif]

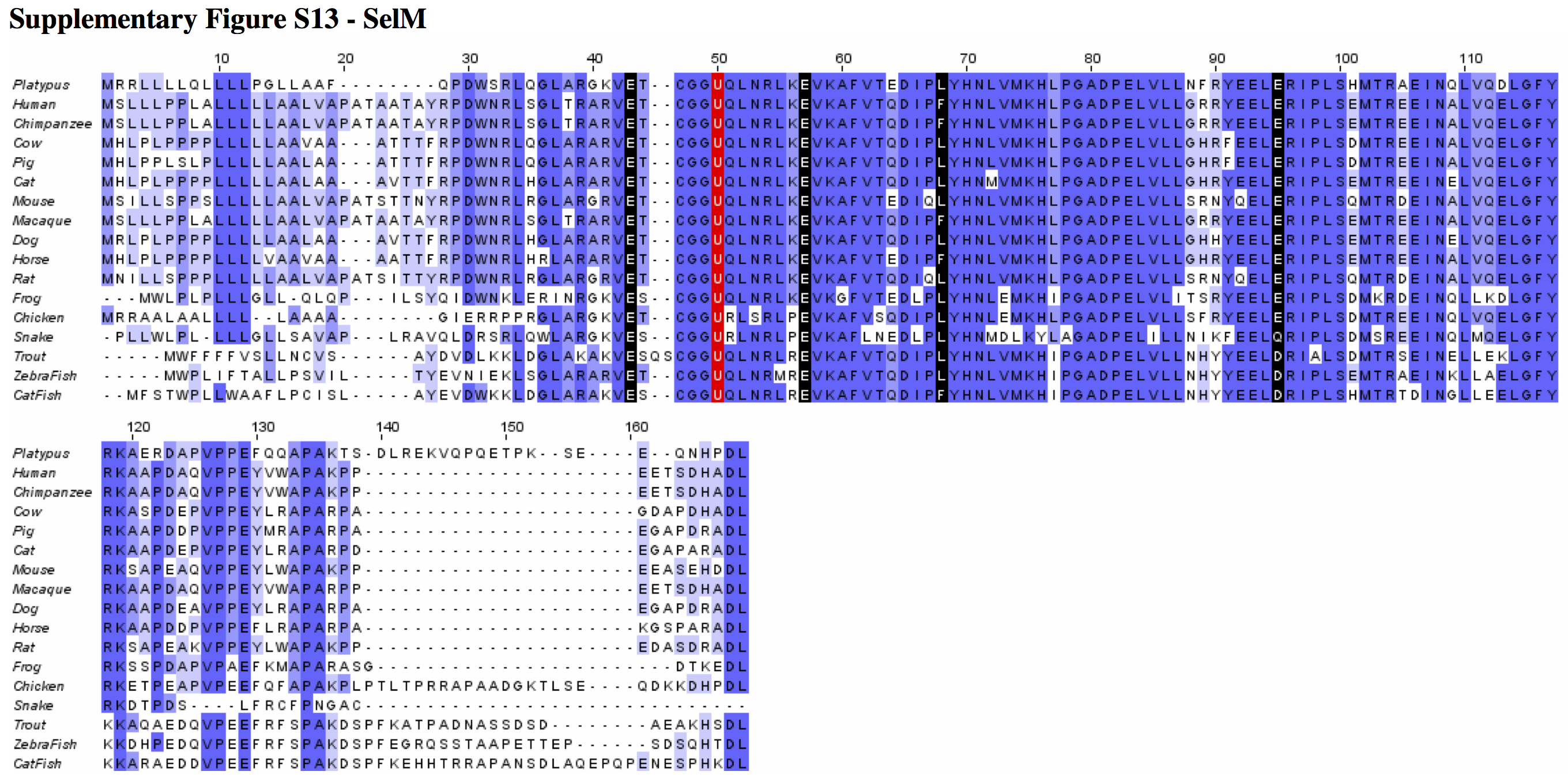

Supplement: Figure S13 — Multiple sequence alignment of SelM. Residues are marked as in Supplementary Figure S1. (TIFF) [file pone.0033066.s013.tif]

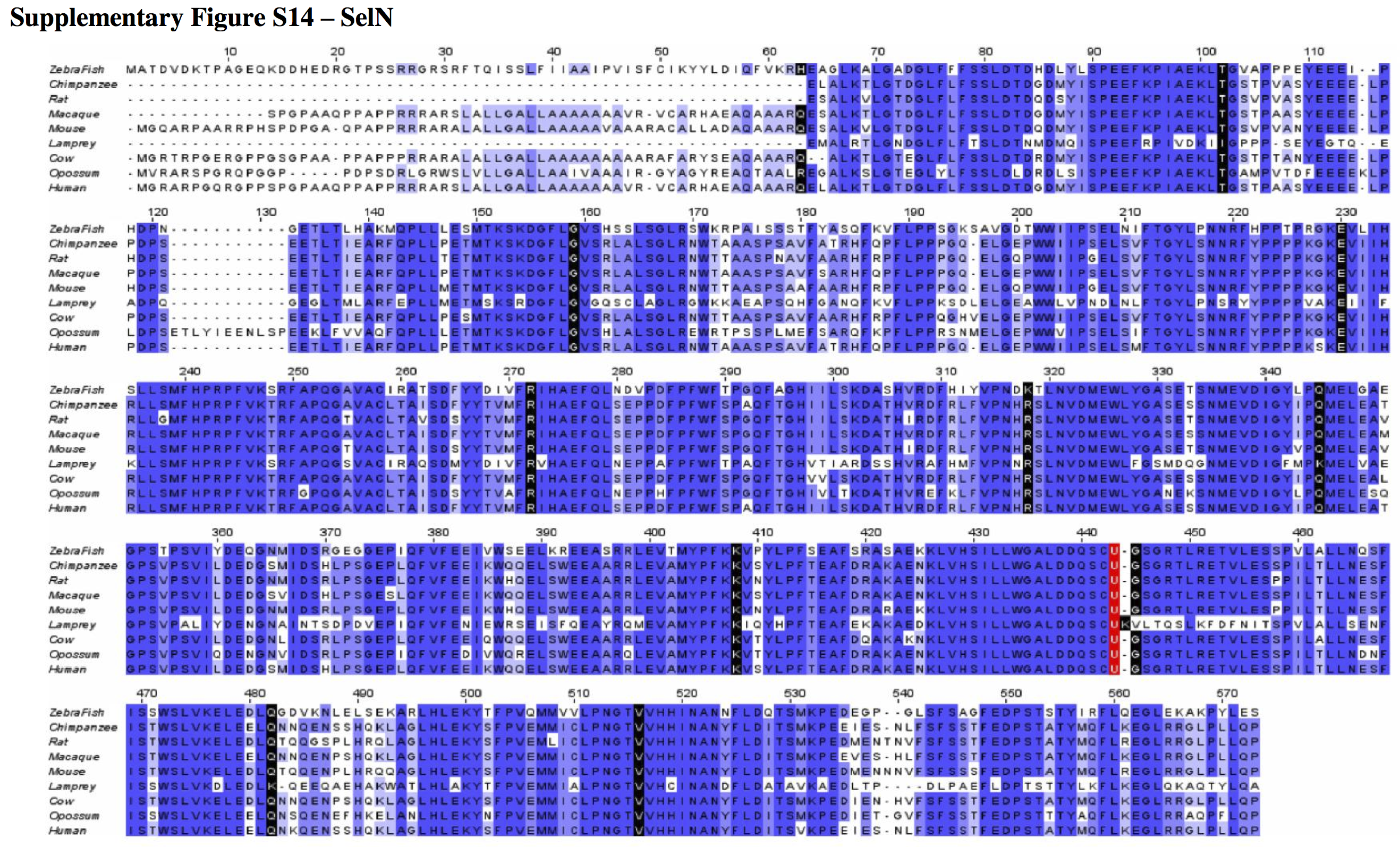

Supplement: Figure S14 — Multiple sequence alignment of SelN. Residues are marked as in Supplementary Figure S1. (TIFF) [file pone.0033066.s014.tif]

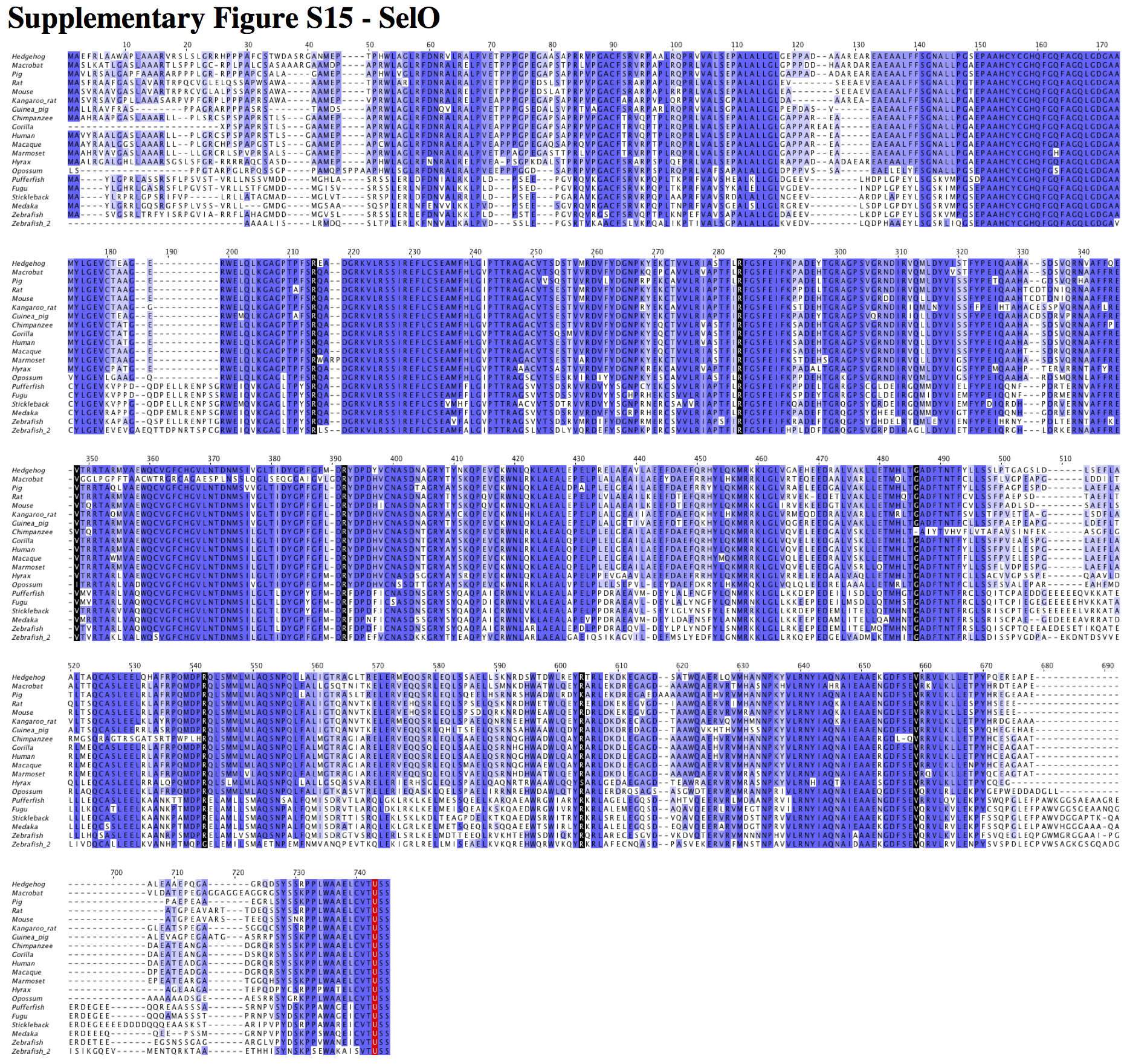

Supplement: Figure S15 — Multiple sequence alignment of SelO. Residues are marked as in Supplementary Figure S1. (TIFF) [file pone.0033066.s015.tif]

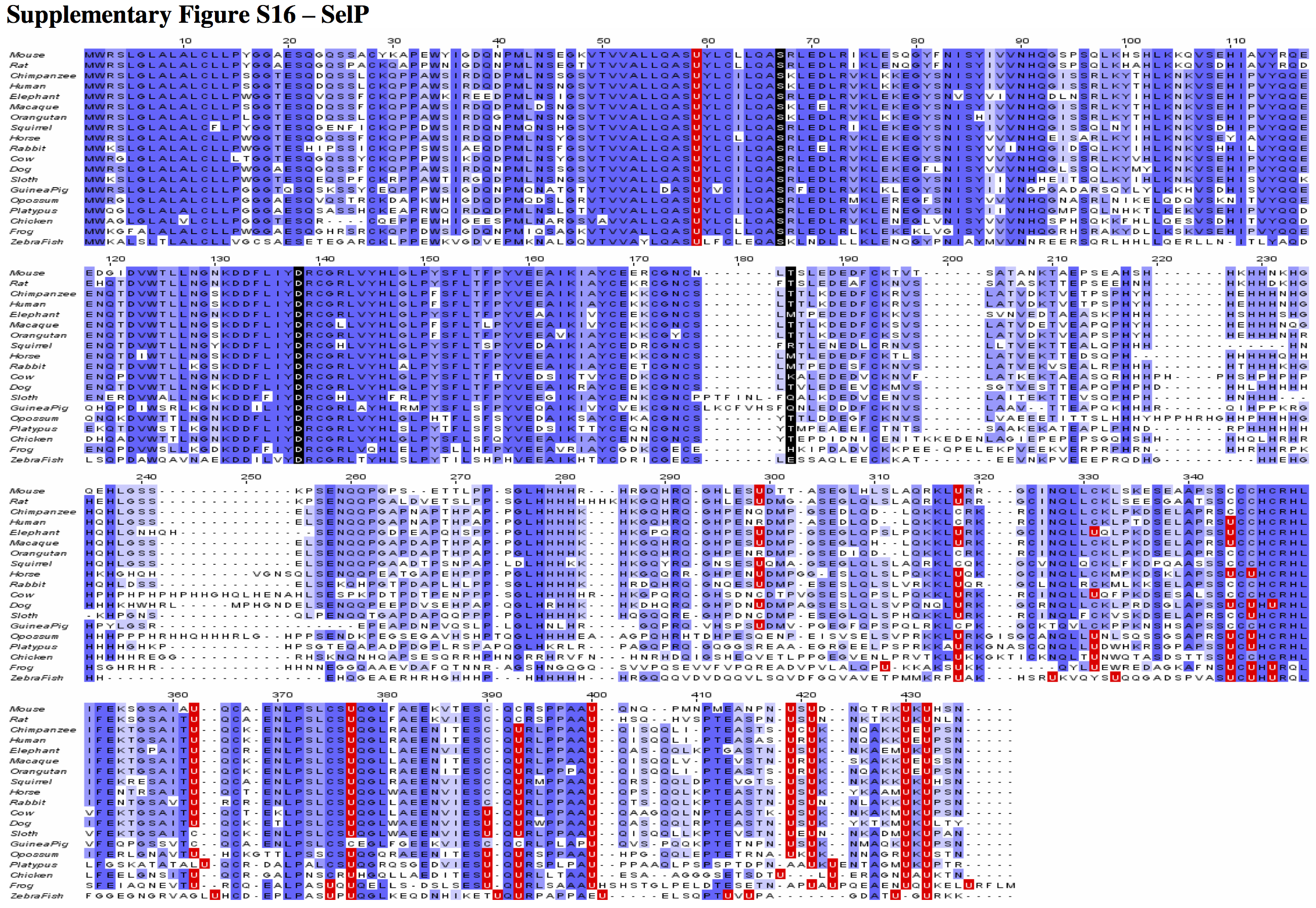

Supplement: Figure S16 — Multiple sequence alignment of SelP. Residues are marked as in Supplementary Figure S1. Note that there are multiple Sec in each protein. (TIFF) [file pone.0033066.s016.tif]

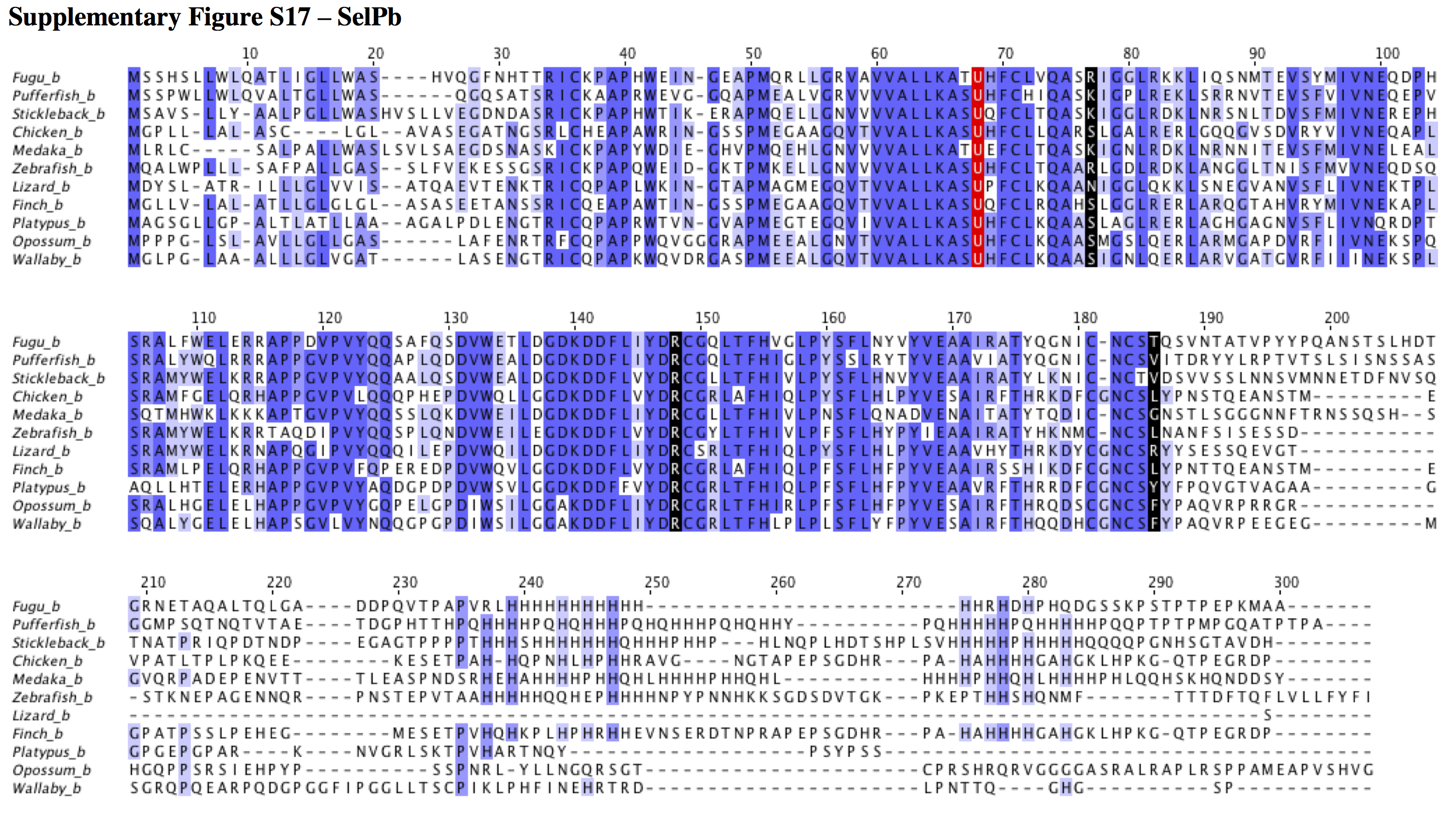

Supplement: Figure S17 — Multiple sequence alignment of SelPb. Residues are marked as in Supplementary Figure S1. (TIFF) [file pone.0033066.s017.tif]

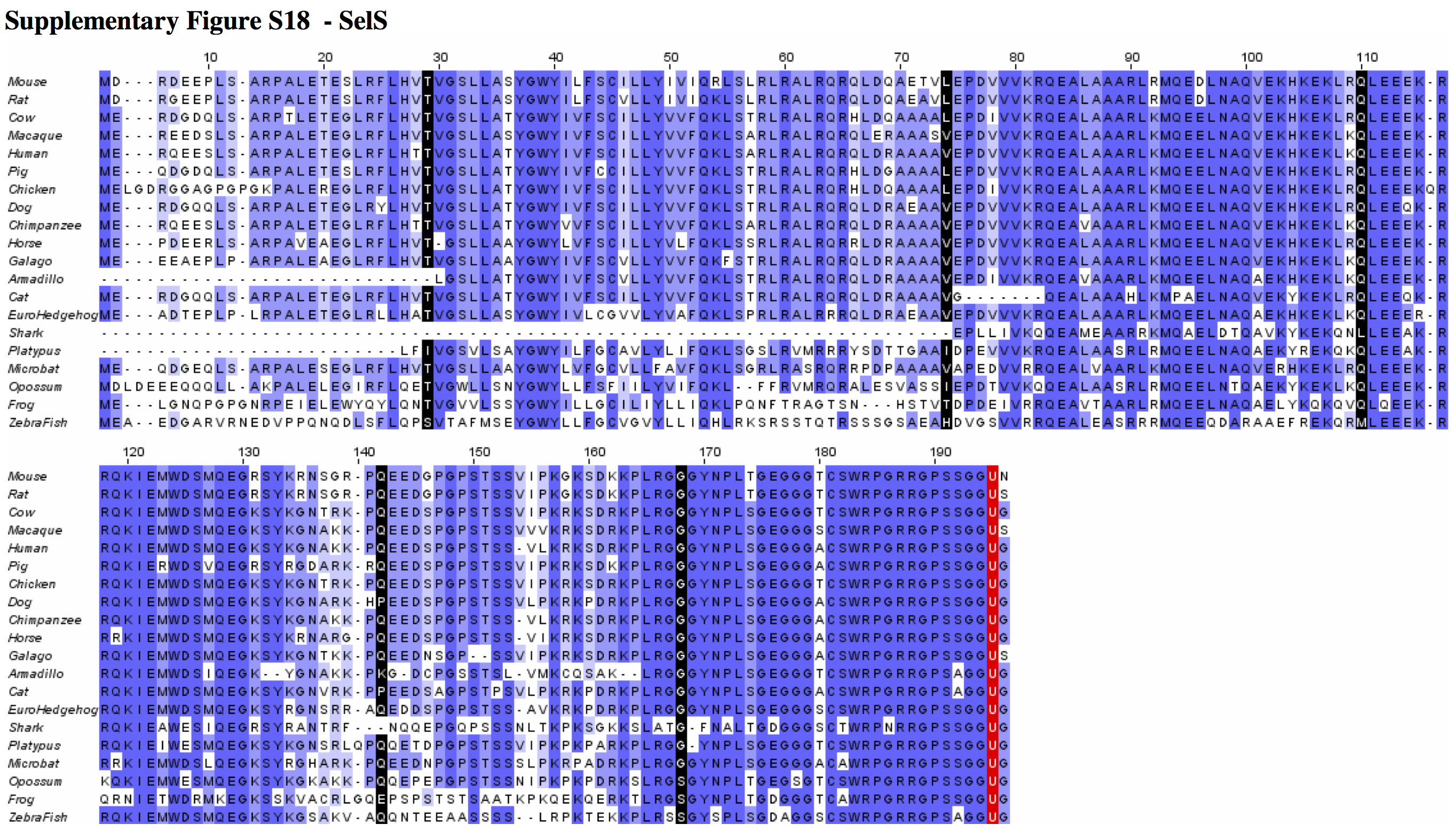

Supplement: Figure S18 — Multiple sequence alignment of SelS. Residues are marked as in Supplementary Figure S1. (TIFF) [file pone.0033066.s018.tif]

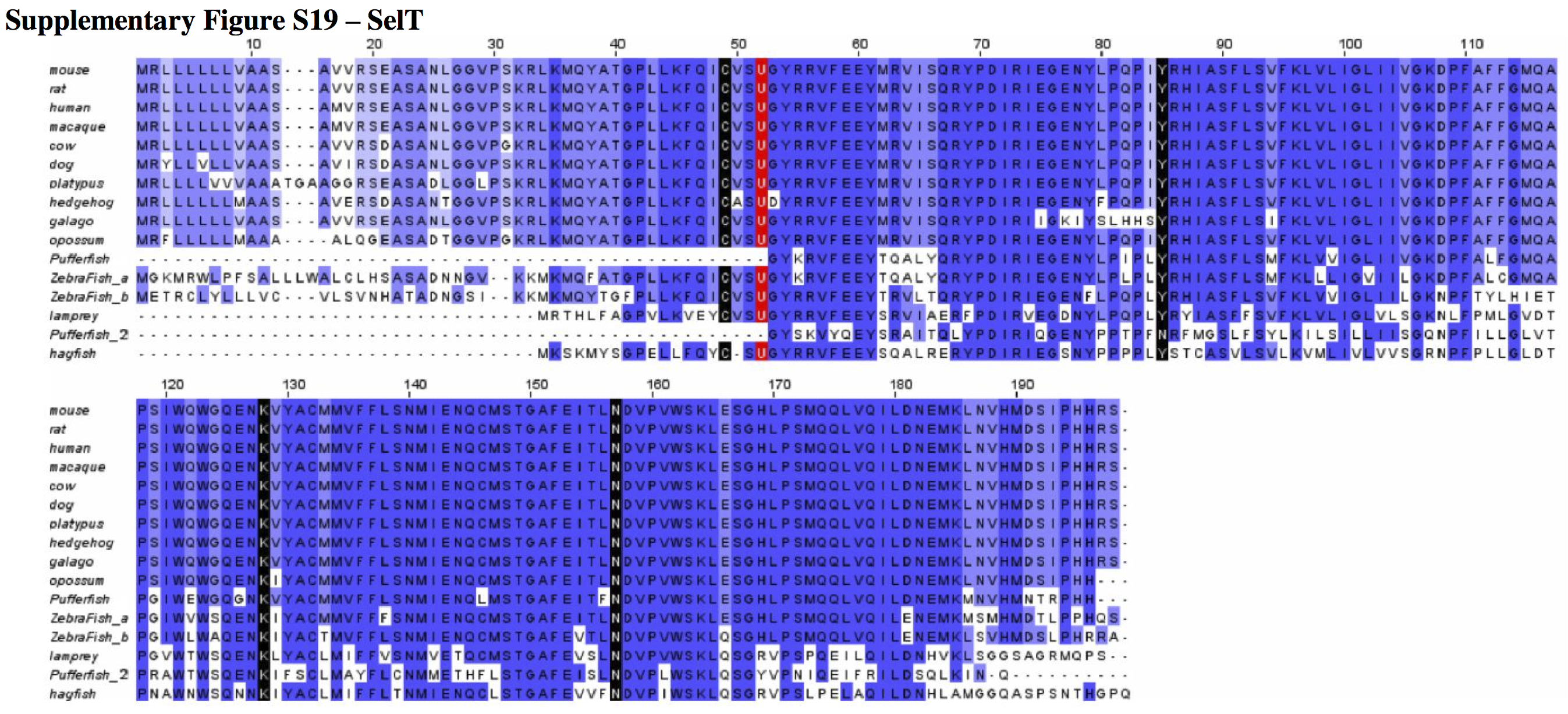

Supplement: Figure S19 — Multiple sequence alignment of SelT. Residues are marked as in Supplementary Figure S1. (TIFF) [file pone.0033066.s019.tif]

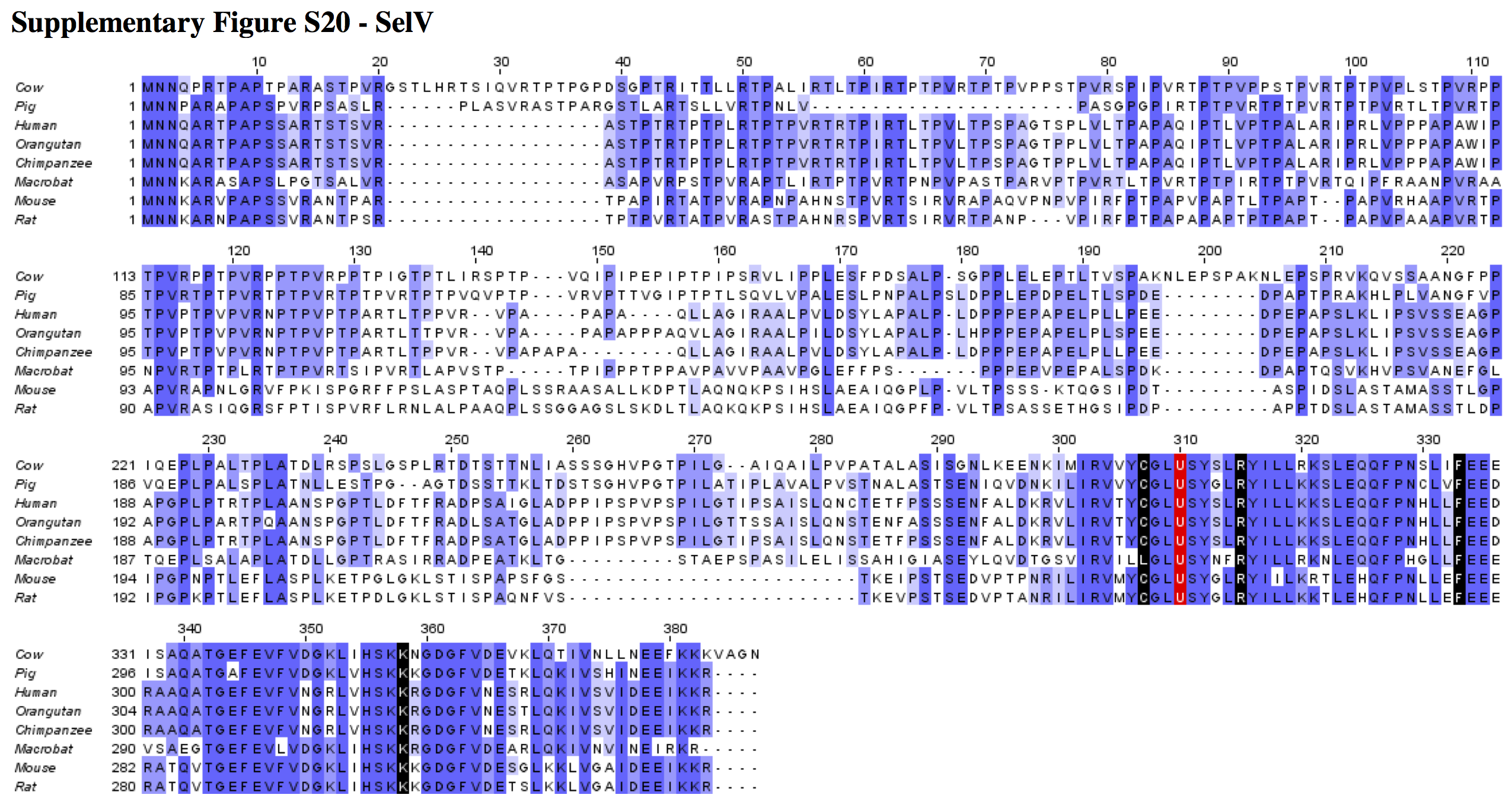

Supplement: Figure S20 — Multiple sequence alignment of SelV. Residues are marked as in Supplementary Figure S1. (TIFF) [file pone.0033066.s020.tif]

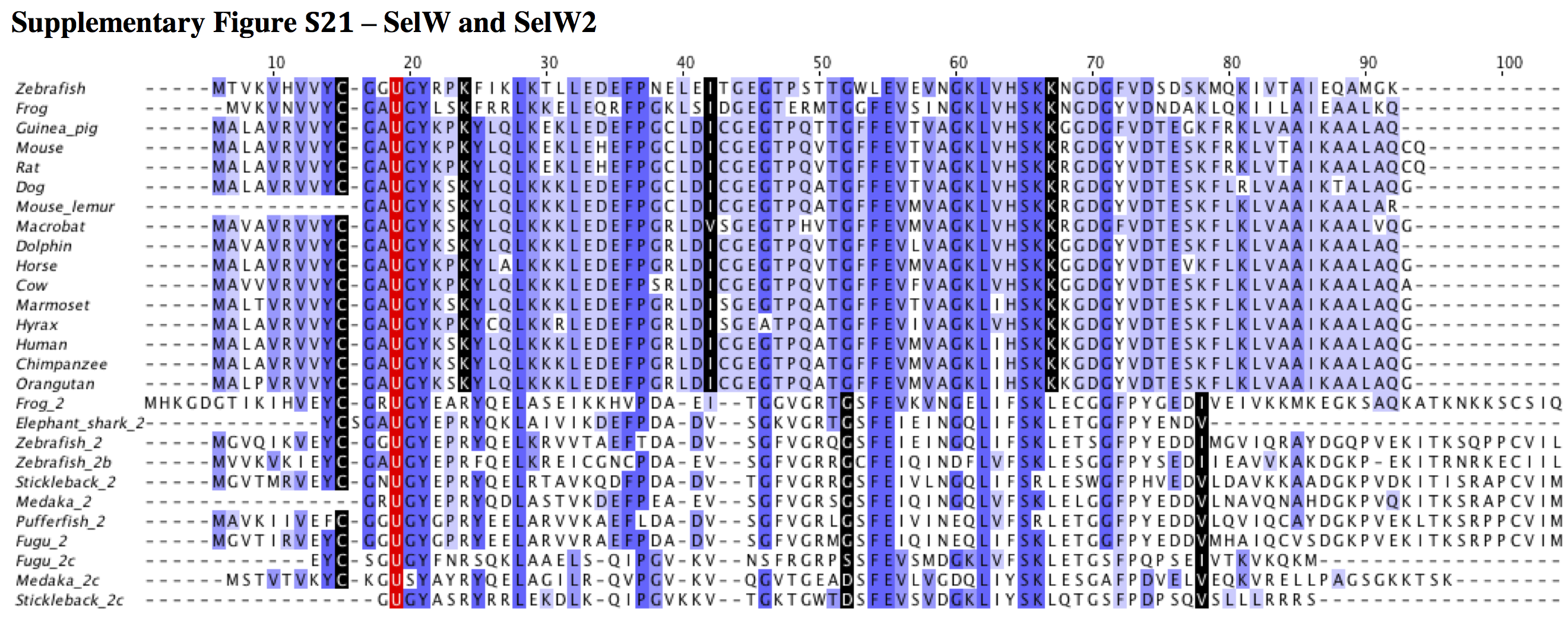

Supplement: Figure S21 — Multiple sequence alignment of SelW and SelW2 proteins. Residues are marked as in Supplementary Figure S1. (TIFF) [file pone.0033066.s021.tif]

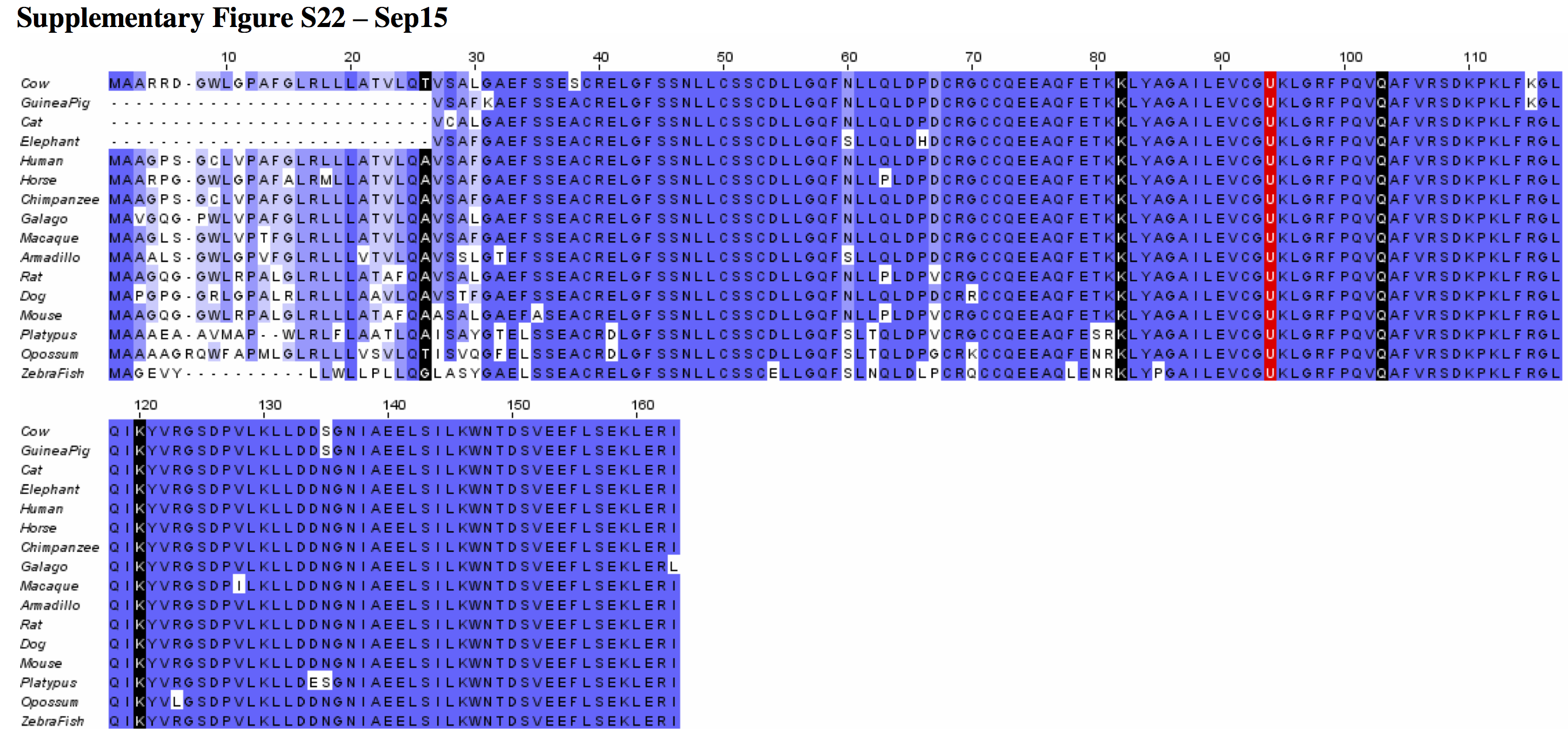

Supplement: Figure S22 — Multiple sequence alignment of Sep15. Residues are marked as in Supplementary Figure S1. (TIFF) [file pone.0033066.s022.tif]

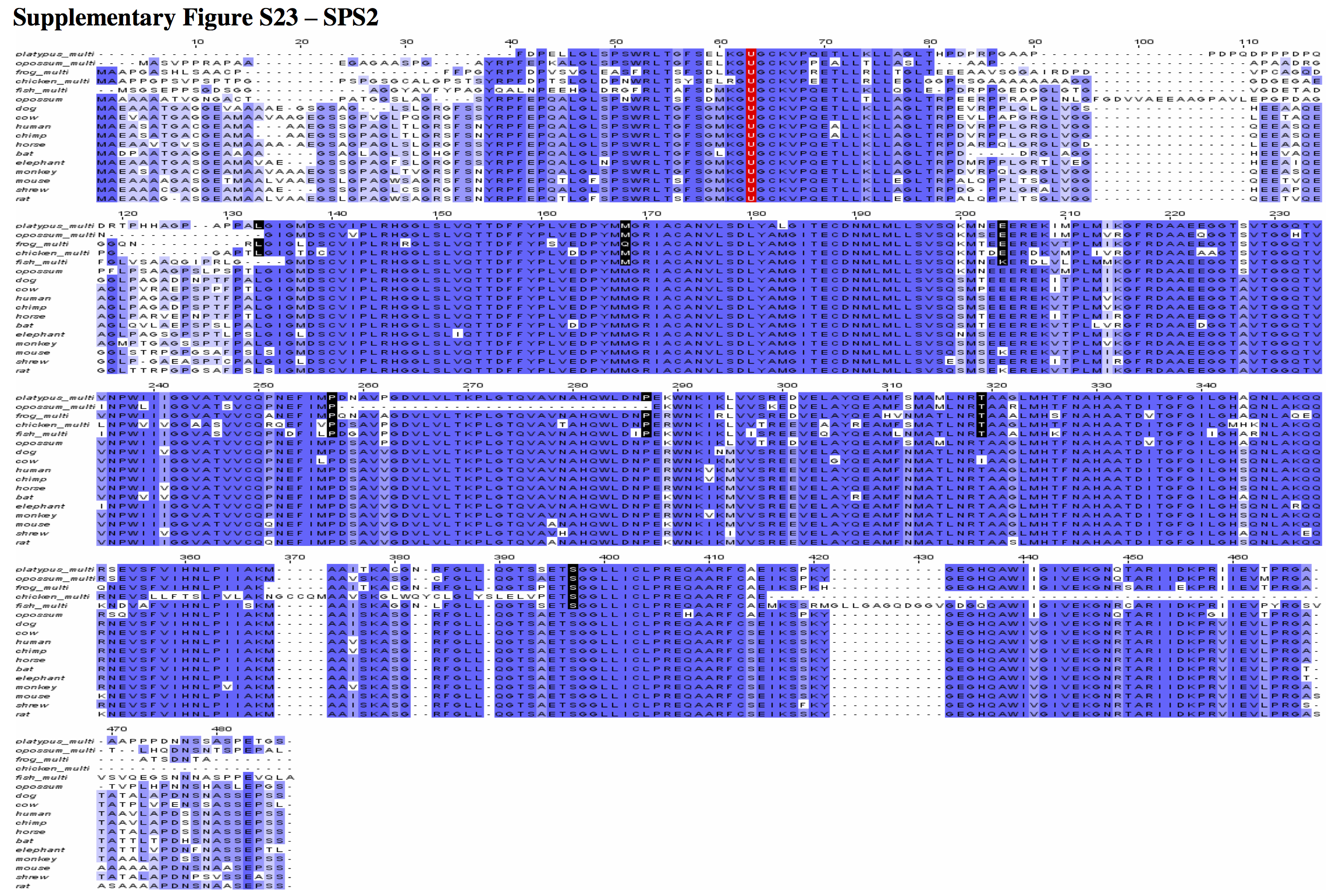

Supplement: Figure S23 — Multiple sequence alignment of SPS2. Residues are marked as in Supplementary Figure S1. Note that in more ancient mammals and vertebrates the SPS2 gene is a multi-exon gene. (TIFF) [file pone.0033066.s023.tif]

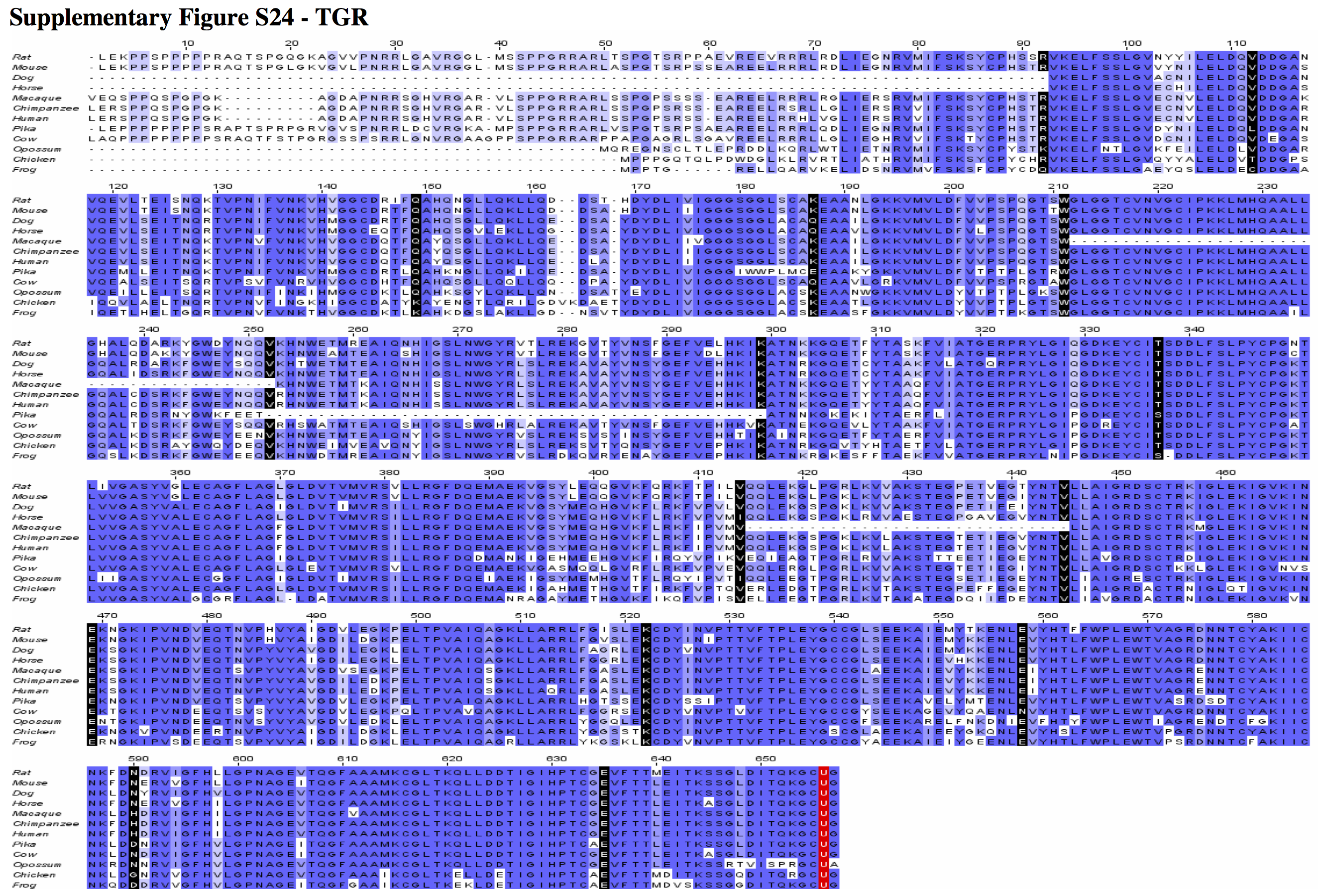

Supplement: Figure S24 — Multiple sequence Alignment of TGR. Residues are marked as in Supplementary Figure S1. (TIFF) [file pone.0033066.s024.tif]

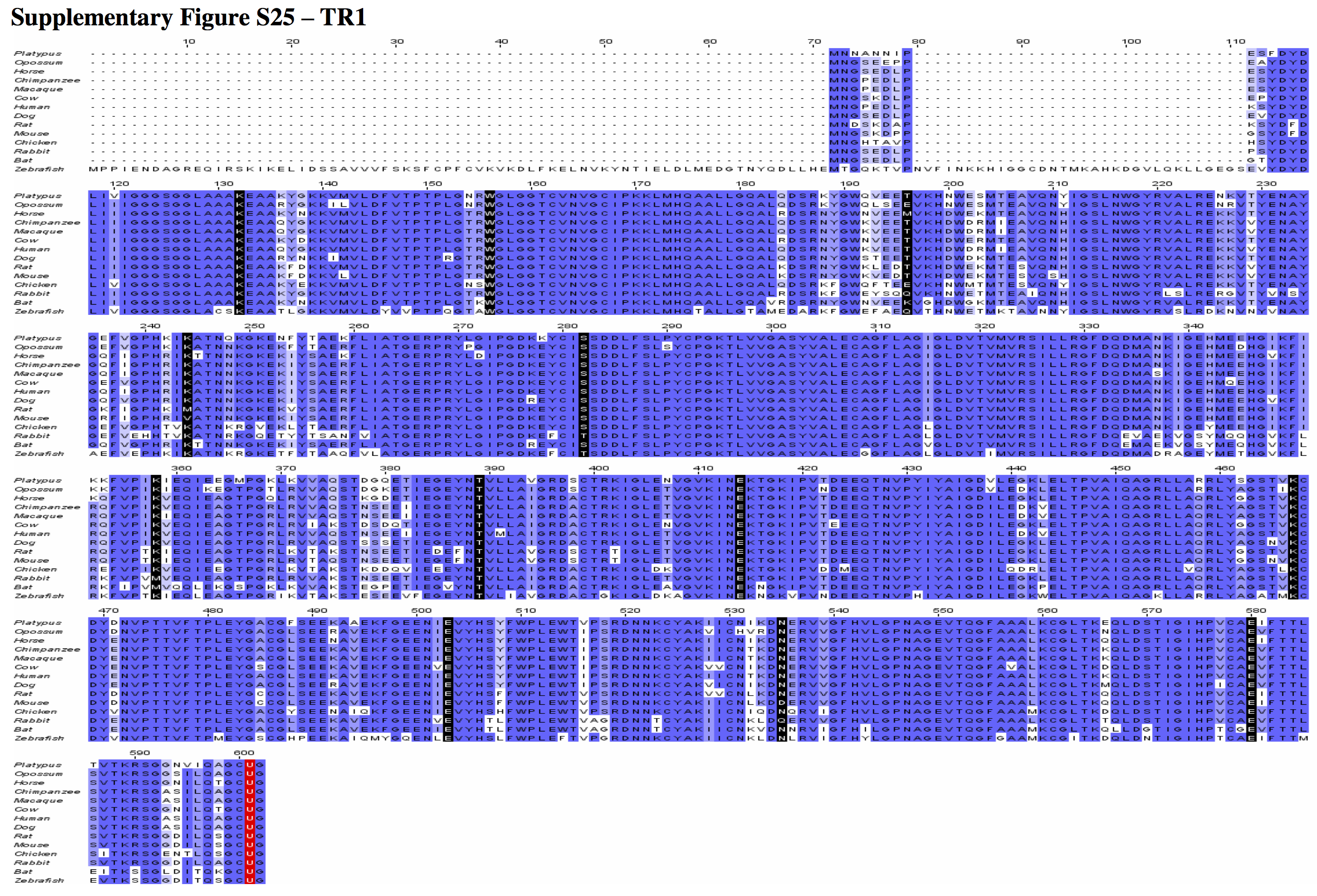

Supplement: Figure S25 — Multiple sequence alignment of TR1. Residues are marked as in Supplementary Figure S1. (TIFF) [file pone.0033066.s025.tif]

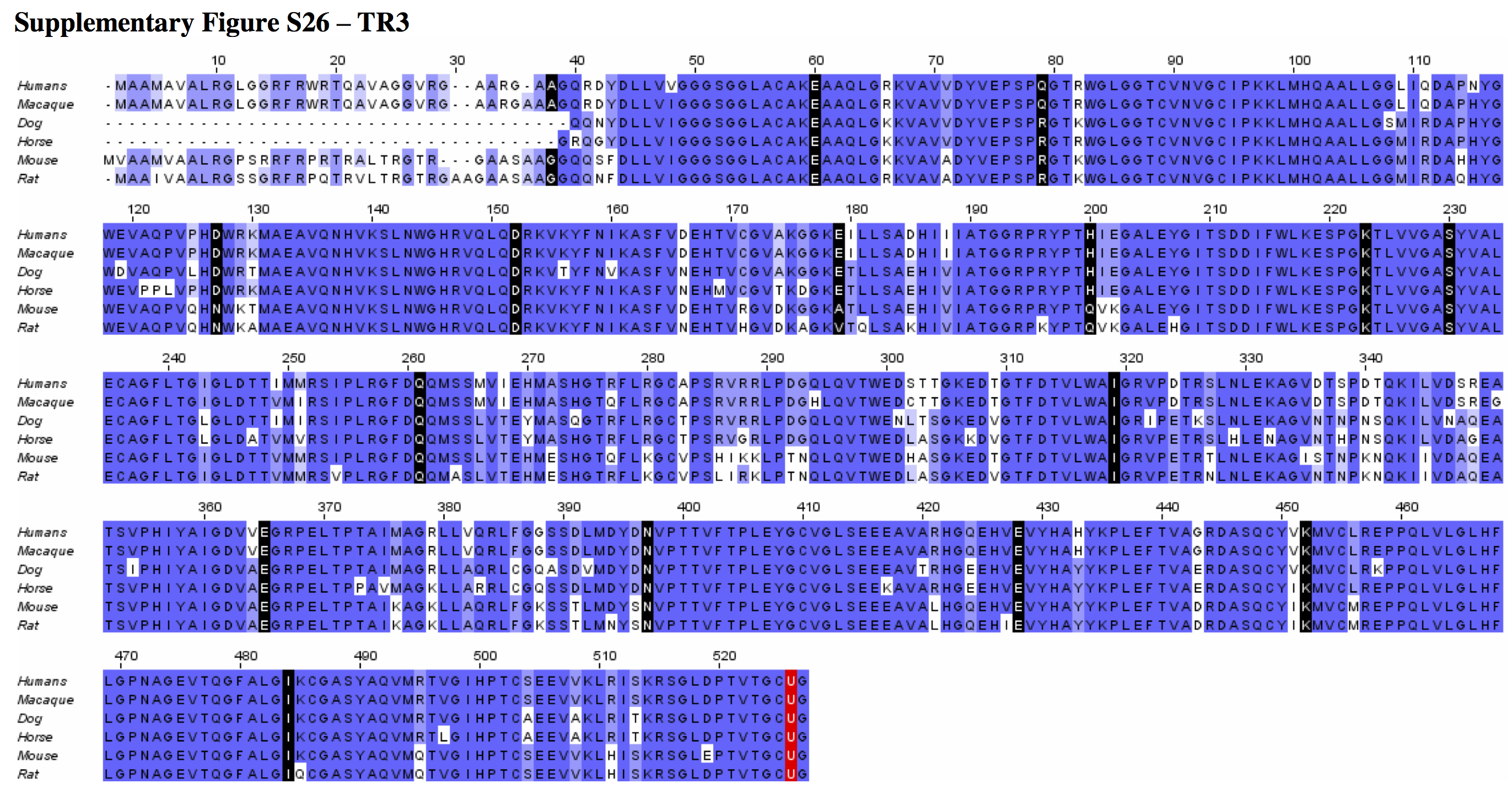

Supplement: Figure S26 — Multiple sequence alignment of TR3. Residues are marked as in Supplementary Figure S1. (TIFF) [file pone.0033066.s026.tif]

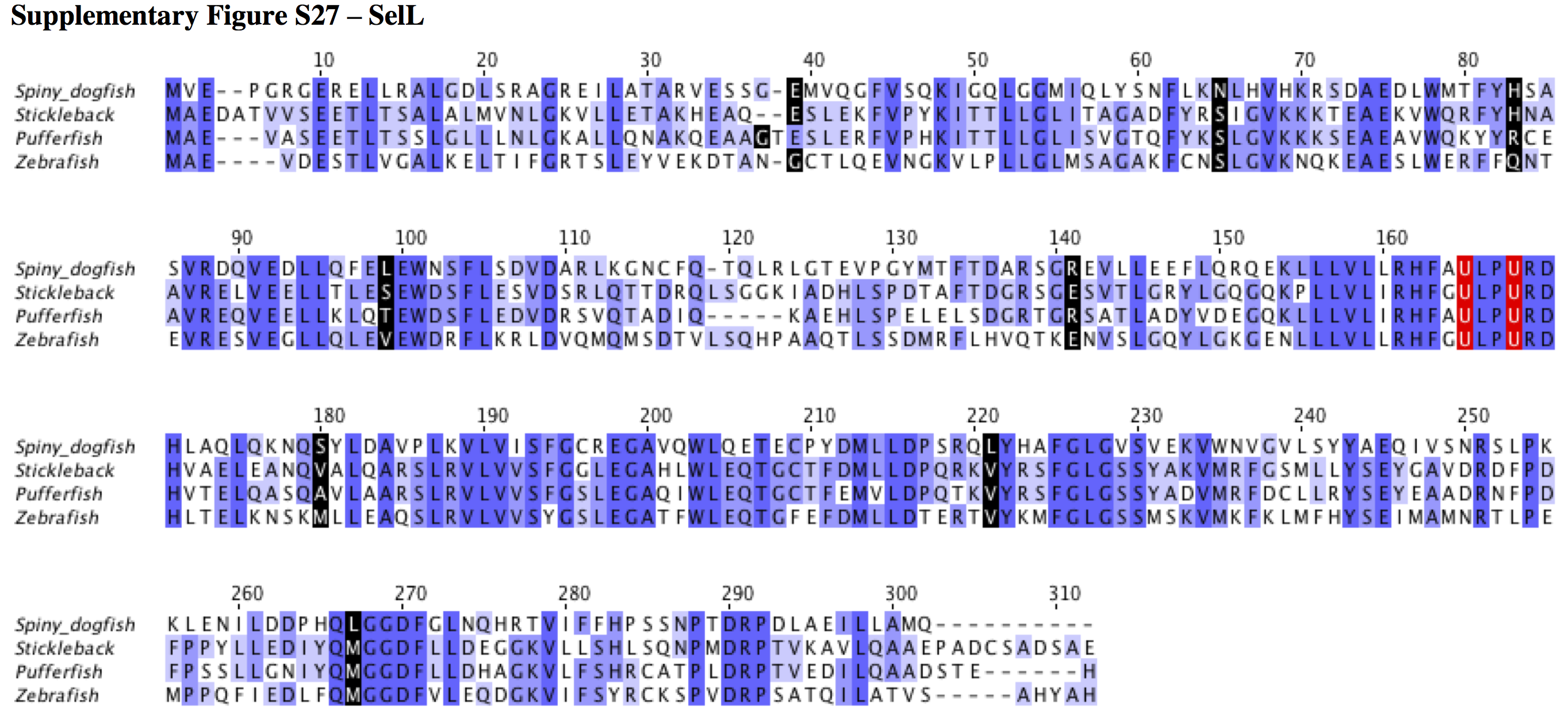

Supplement: Figure S27 — Multiple sequence alignment of SelL. The Sec is shown in red. (TIFF) [file pone.0033066.s027.tif]

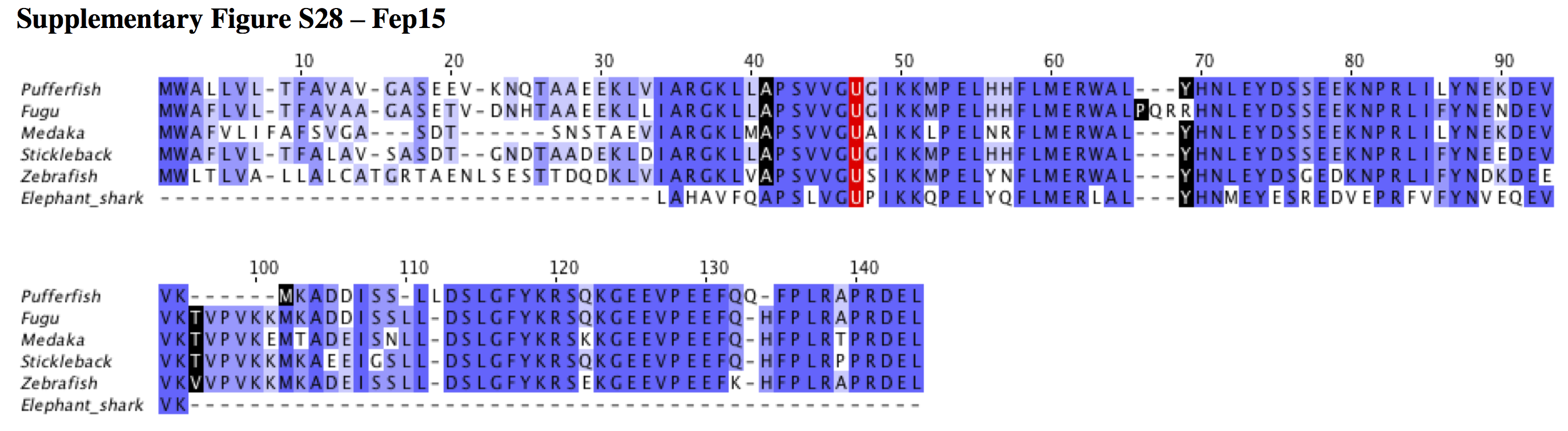

Supplement: Figure S28 — Multiples sequence alignment of Fep15. Residues are marked as in Supplementary Figure S1. (TIFF) [file pone.0033066.s028.tif]

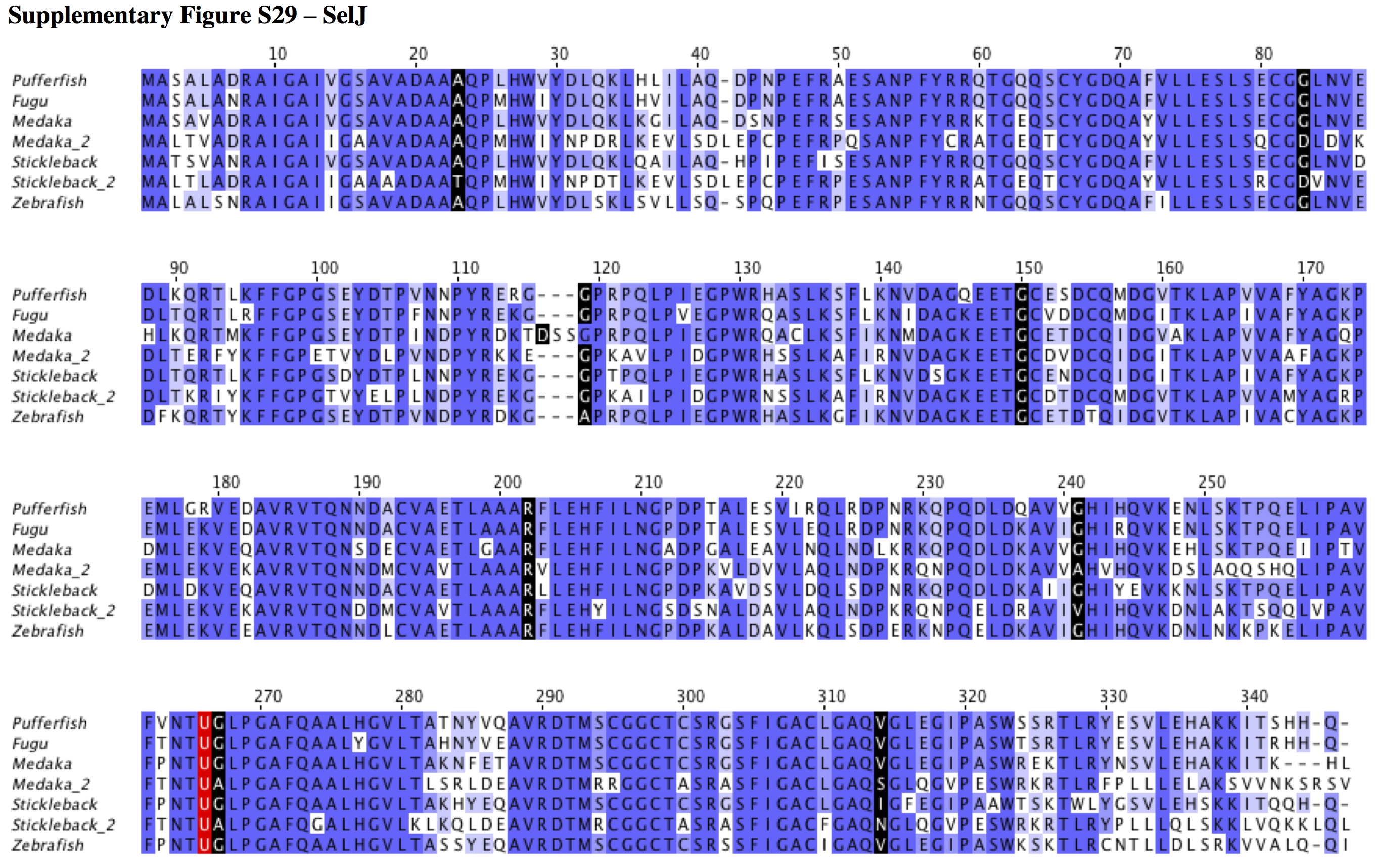

Supplement: Figure S29 — Multiple sequence alignment of SelJ. Residues are marked as in Supplementary Figure S1. (TIFF) [file pone.0033066.s029.tif]

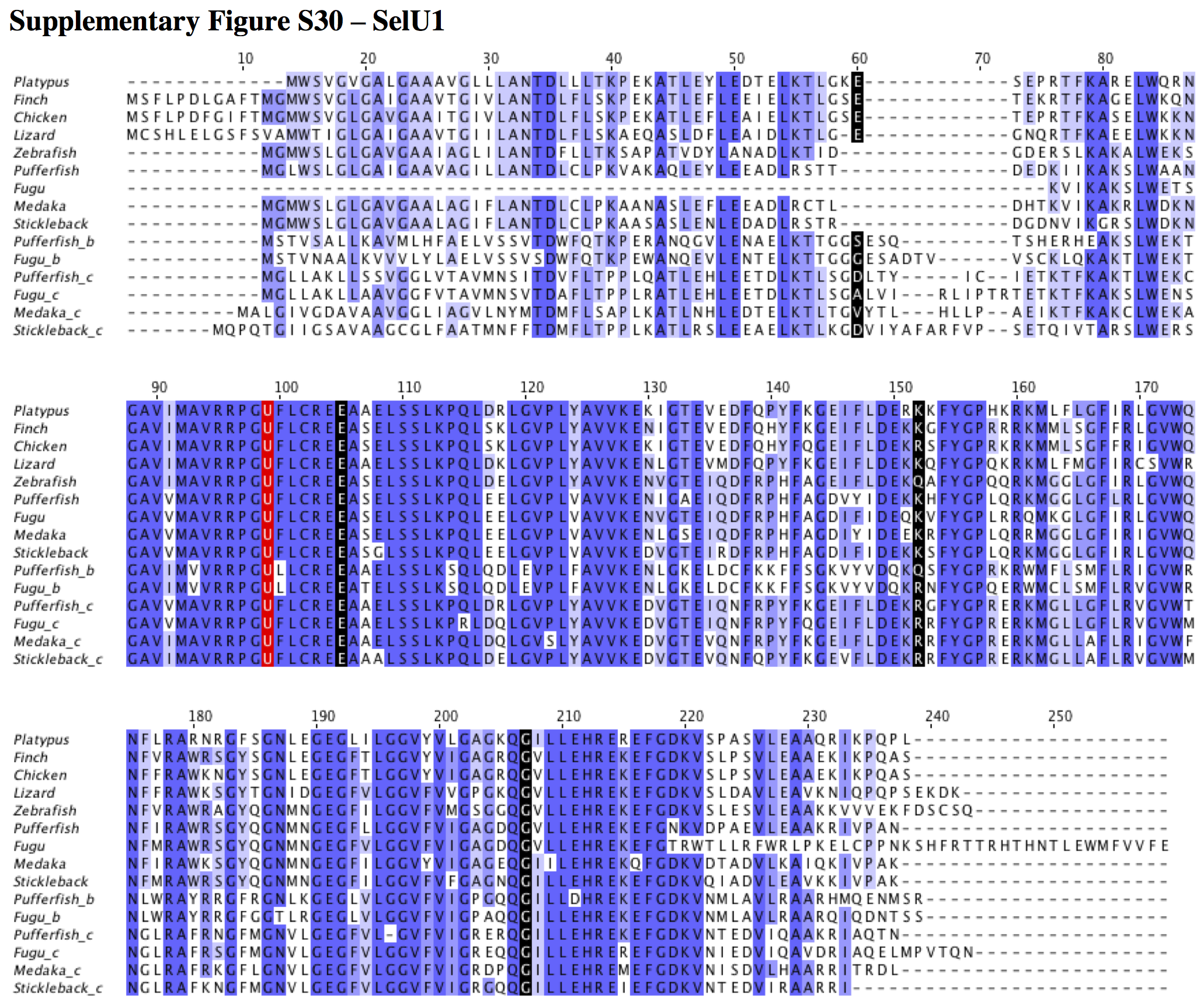

Supplement: Figure S30 — Multiple sequence alignment of SelU1. Residues are marked as in Supplementary Figure S1. (TIFF) [file pone.0033066.s030.tif]

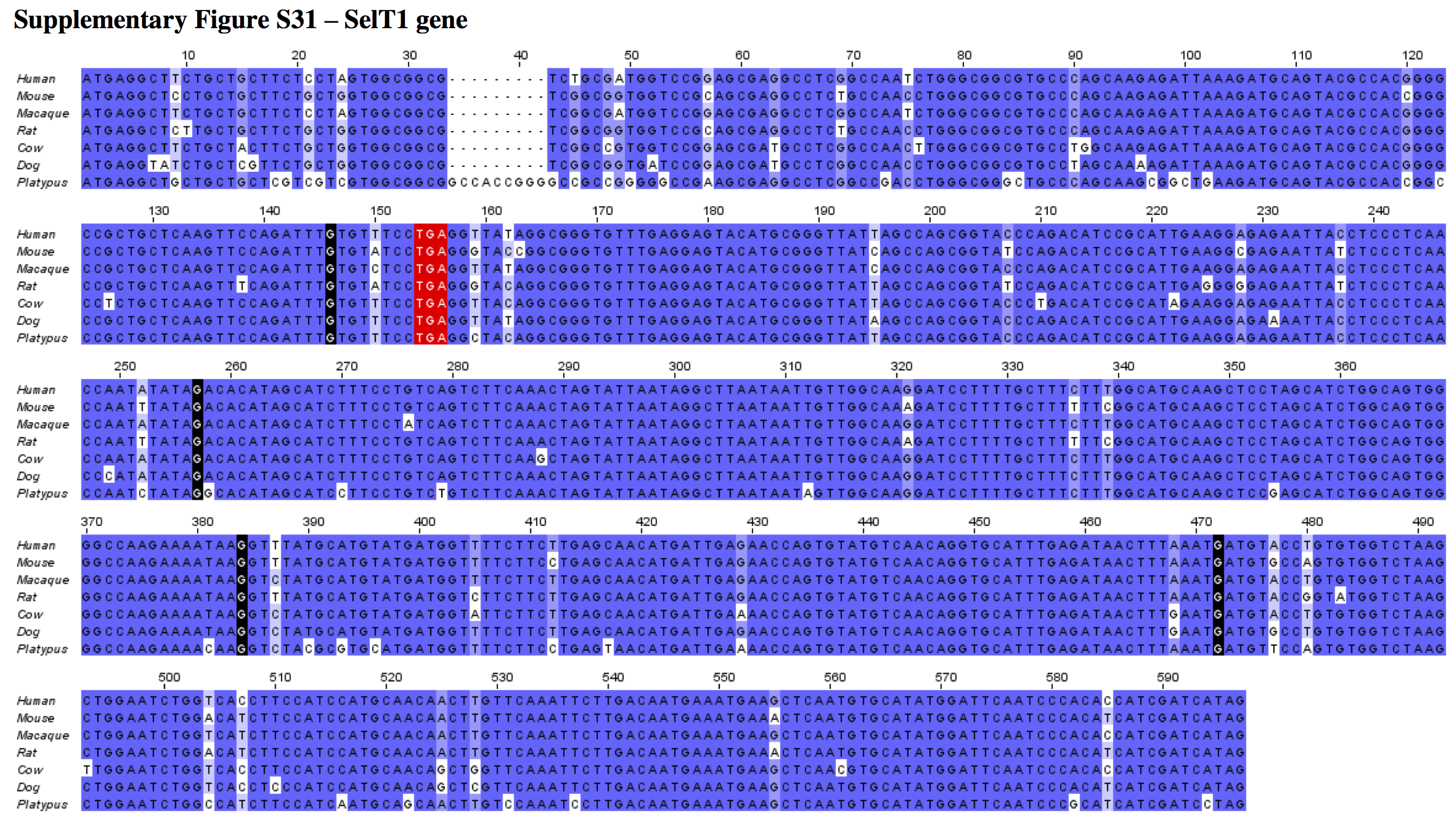

Supplement: Figure S31 — Multiple sequence alignment of mammalian SelT1 coding sequences. The last residue of each exon is marked in black, and the codon corresponding to the Sec is shown in red. (TIFF) [file pone.0033066.s031.tif]

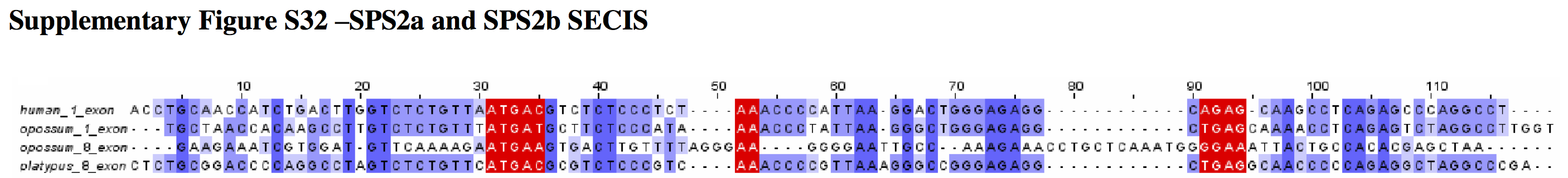

Supplement: Figure S32 — SECIS in SPS2a and SPSb. Multiple sequence alignment of opossum SPS2a and SPS2b, platypus SPS2a, and human SPS2b SECIS elements. Critical portions are marked in red. (TIFF) [file pone.0033066.s032.tif]

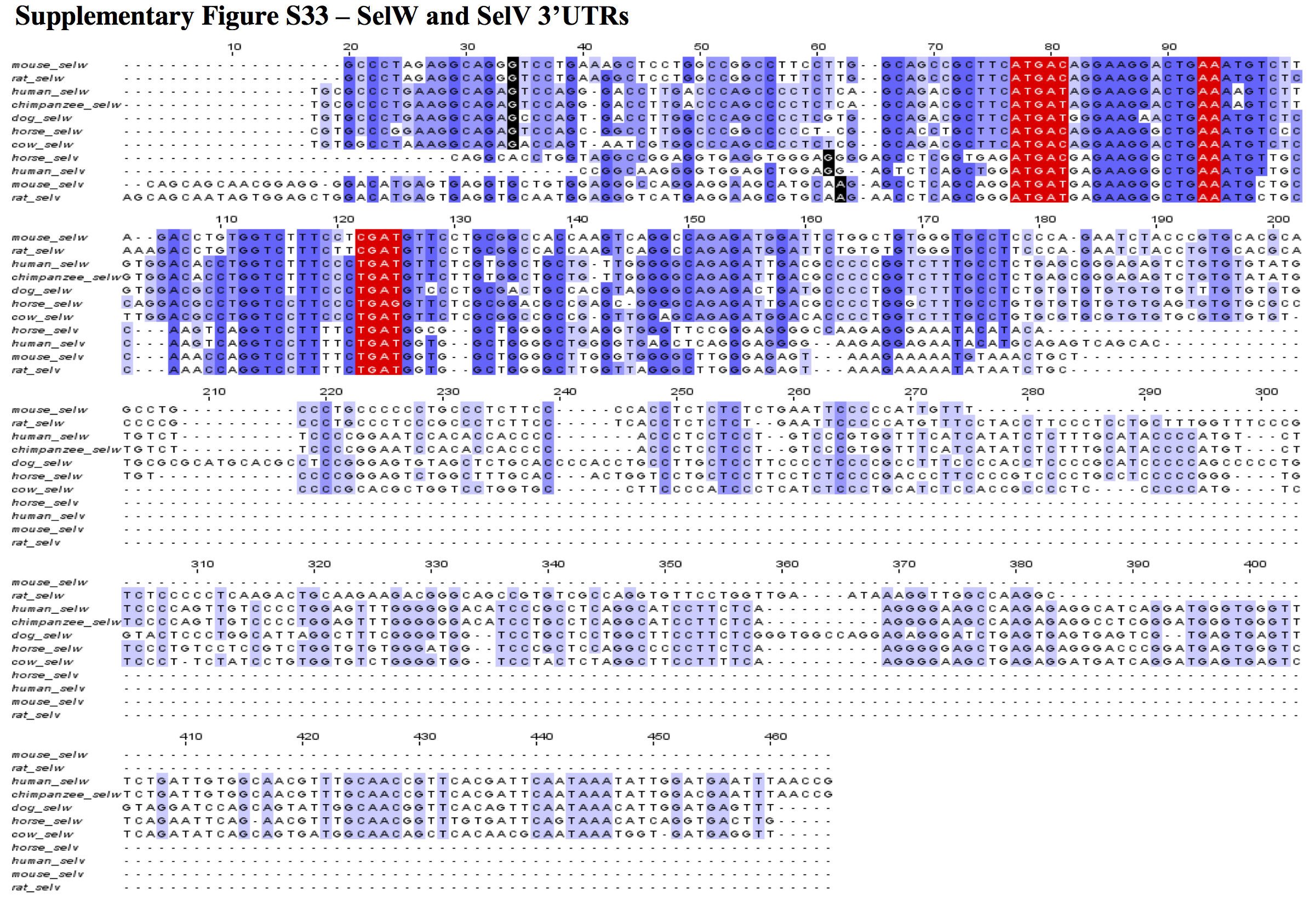

Supplement: Figure S33 — Multiple sequence alignment of SelW and SelV 3′-UTRs. Critical portions of the SECIS elements are marked in red. The last nucleotide of exon 5 is marked in black. (TIFF) [file pone.0033066.s033.tif]

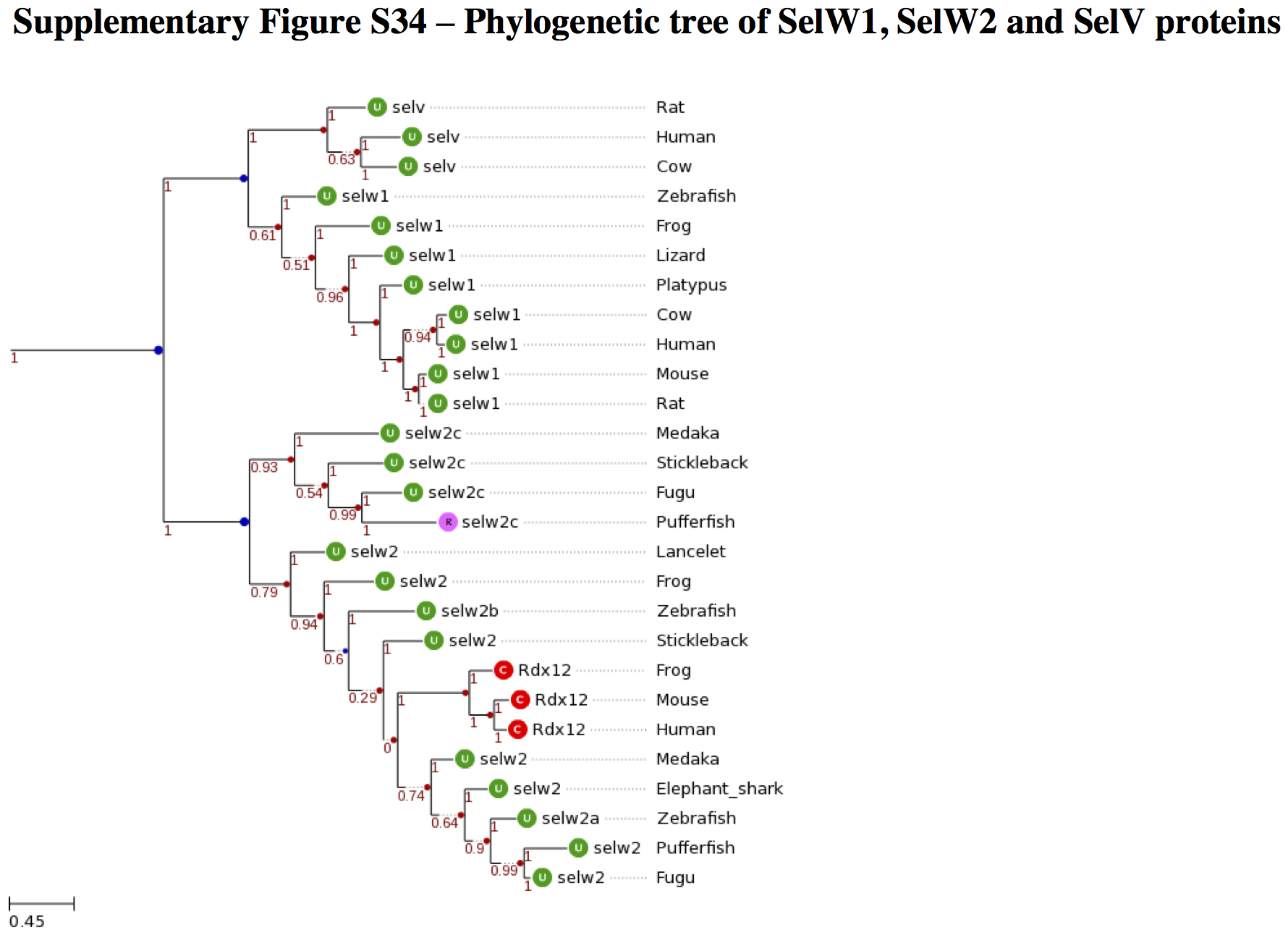

Supplement: Figure S34 — Phylogenetic tree of SelW1, SelW2 and SelV proteins. ML phylogenetic tree of SelW1, SelW2 and SelV protein sequences computed using the WAG substitution model. The branch support for each node (computed as described in the methods) is shown in red. The bar at the bottom left shows the scale in substitutions per position. The Rdx12 gene was found in all tetrapodes but only frog, mouse and human were included in the phylogenetic tree. In contrast all SelW2 detected were included: this gene is missing in all tetrapodes apart from frog. SelW1 is missing from bony fishes apart from zebrafish. SelV was detected in all placentals except gorilla but only rat, cow and human were included. Note that while the SelV-SelW1 duplication is clear and well supported, the rest of the tree is more confused. Nonetheless SelW2c, SelW2b and Rdx12 appear to have been generated by independent duplications. (TIFF) [file pone.0033066.s034.tif]

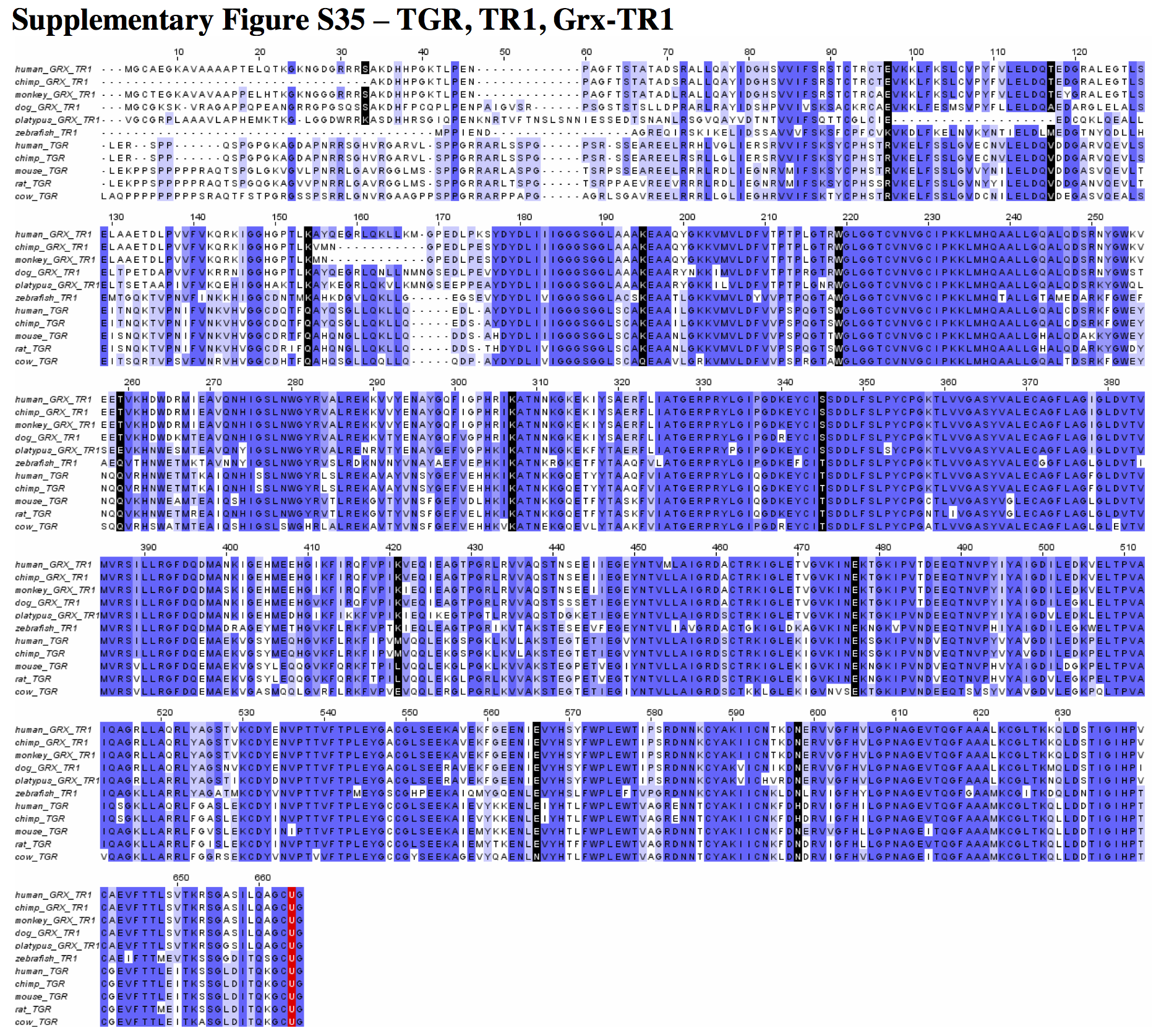

Supplement: Figure S35 — Multiple sequence alignment of TGR, zebrafish TR1, and GRx-containing TR1. Residues are marked as in Figure S1. Note positions where zebrafish TR1 and TGR match, but are different than GRx-containing TR1 (i.e., positions 43, 142, 143, 149, 150, 324, etc.). (TIFF) [file pone.0033066.s035.tif]

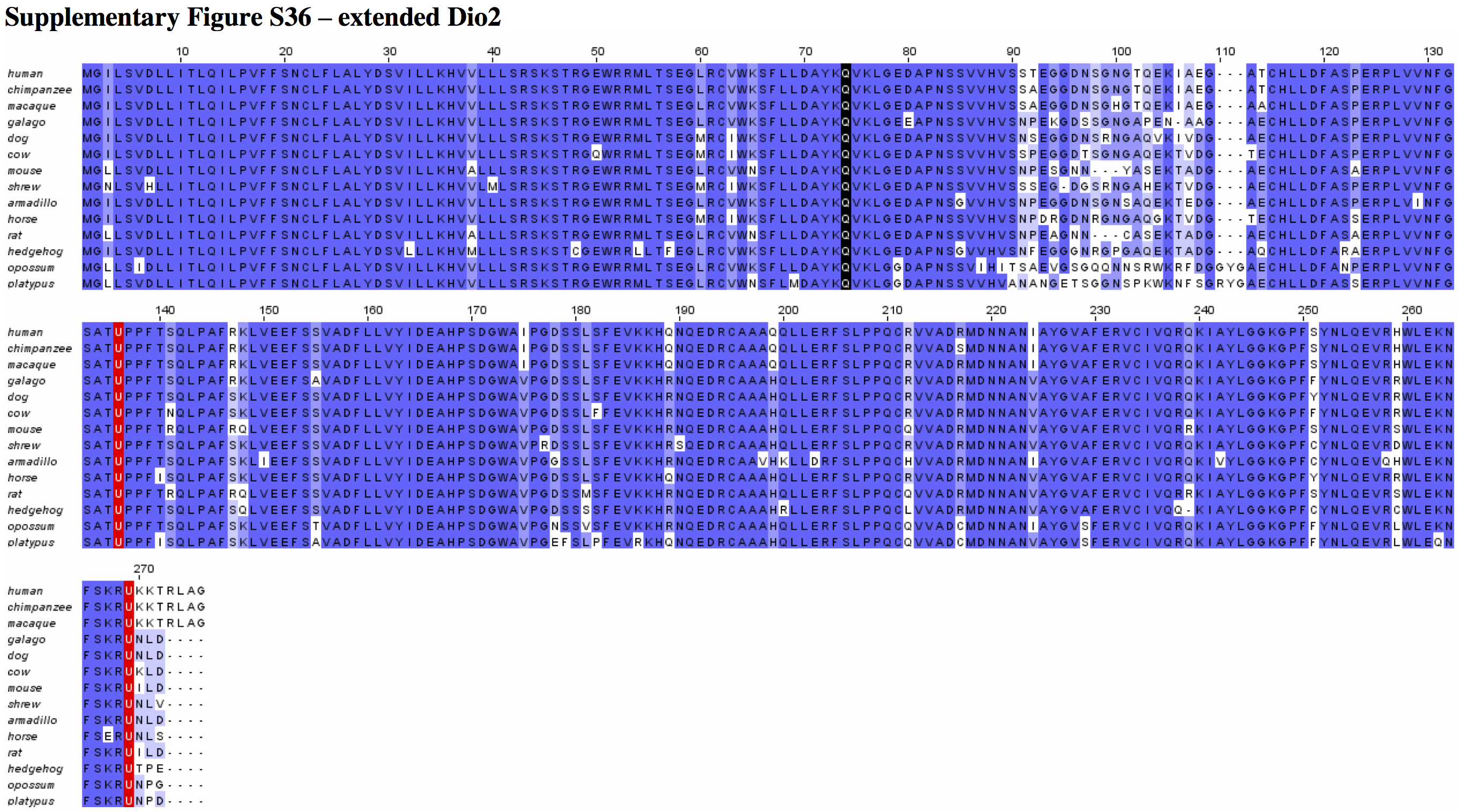

Supplement: Figure S36 — Multiple sequence alignment of extended Dio2 sequences. The last residue of each exon is marked in black and the Sec residues in red. The second Sec, residue 269, is the stop codon or potential second Sec. (TIFF) [file pone.0033066.s036.tif]

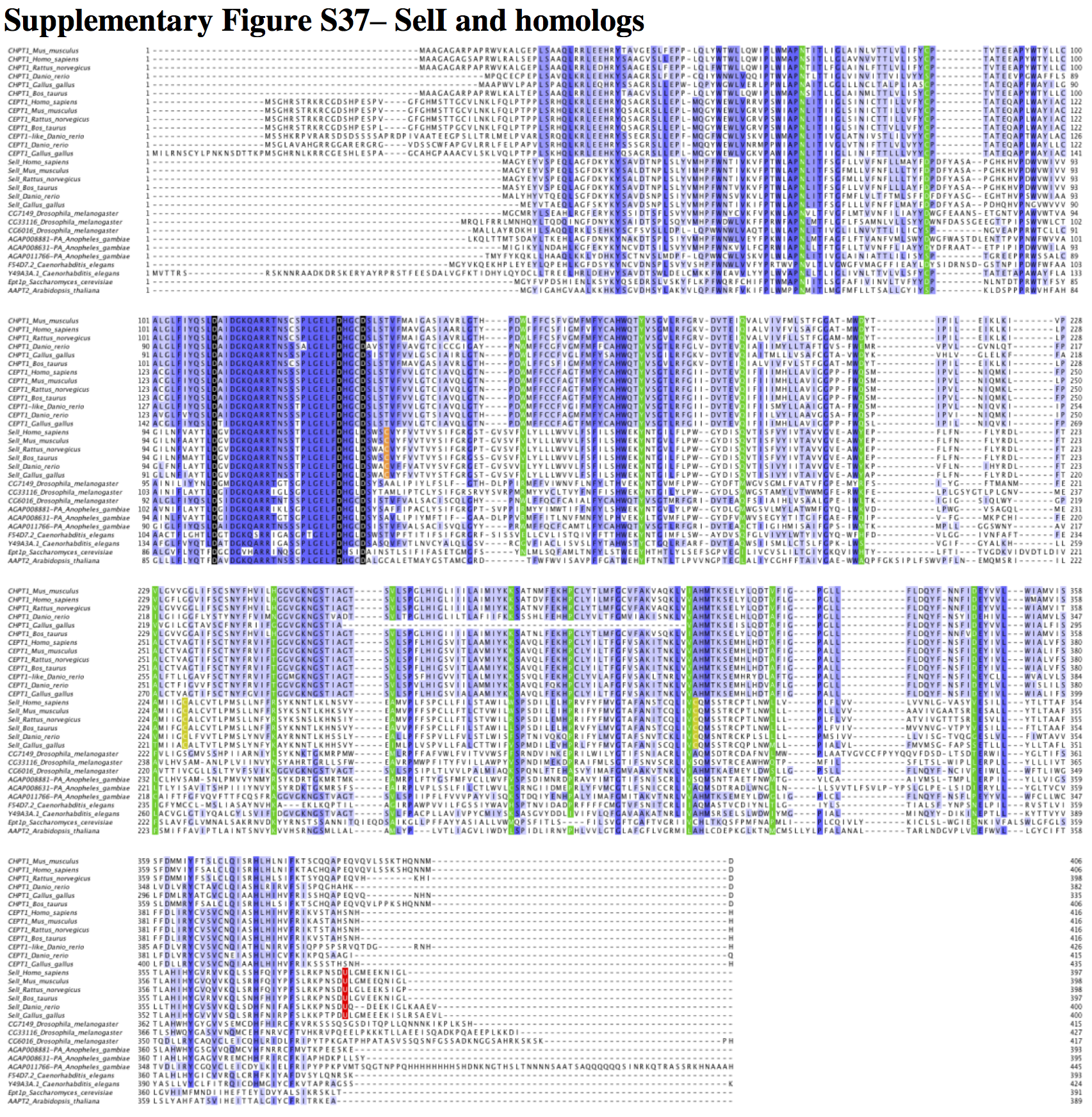

Supplement: Figure S37 — Multiple sequence alignment of SelI and its sequence homologs. Homologs are labeled with the annotated name. Some sequences not annotated as CHPT1 or CEPT1 were also included, as they contain the same domain. Important residues in the active sites are marked in red. The last residue of each side of all predicted transmembrane regions are marked in green. Selenocysteines are marked in red. The cysteines emerged specifically in SelI proteins are also marked: the best candidate (near the catalytic side, on the same side of membrane of Sec) is marked in orange, while the other 2 cysteines are marked in yellow. (TIFF) [file pone.0033066.s037.tif]

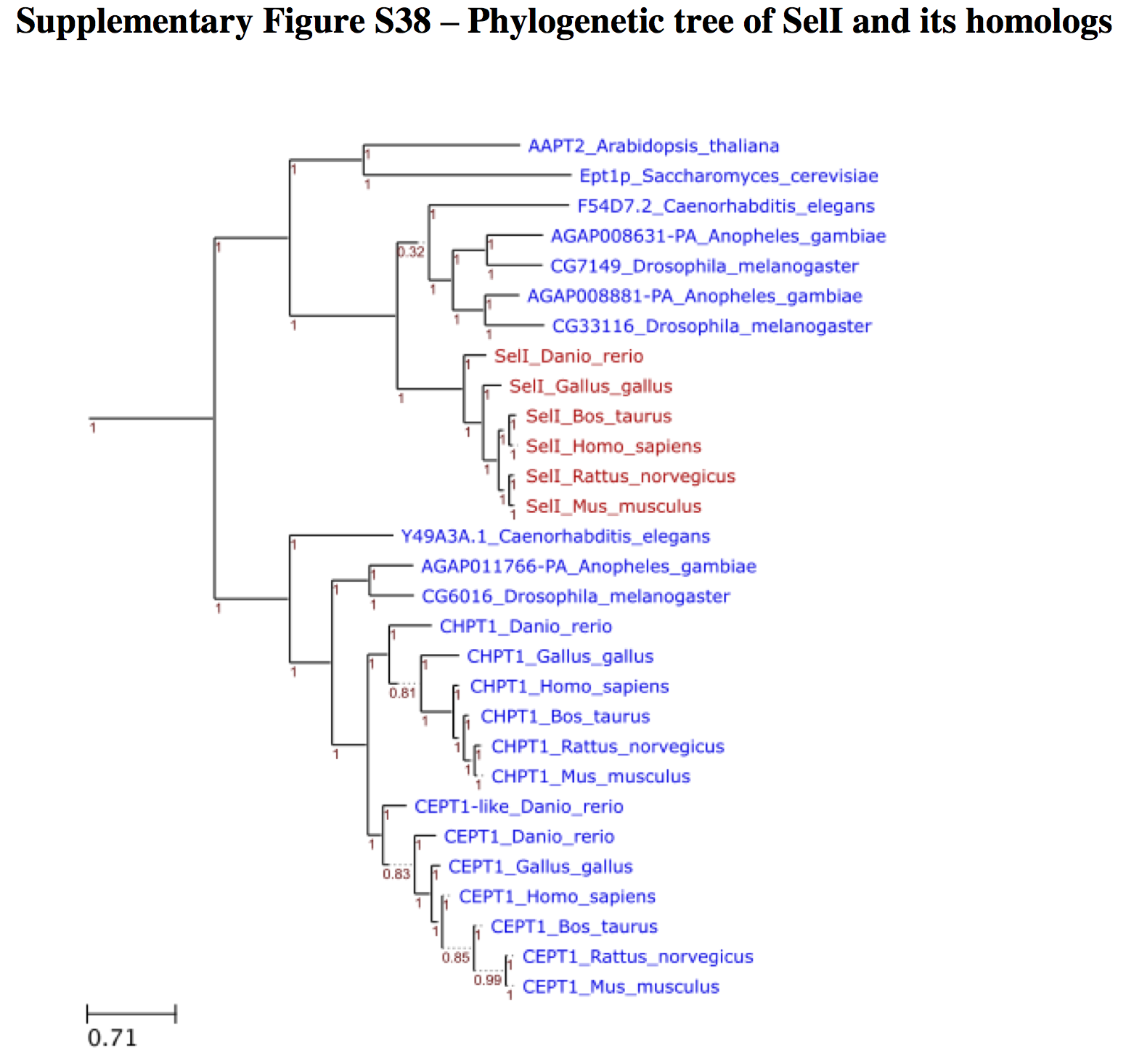

Supplement: Figure S38 — Phylogenetic tree of SelI and its sequence homologs. ML tree computed using WAG model and the alignment shown in supplementary figure S37. The bar at the bottom left shows the scale in substitutions per position, while the branch support for each node is shown in red. (TIFF) [file pone.0033066.s038.tif]

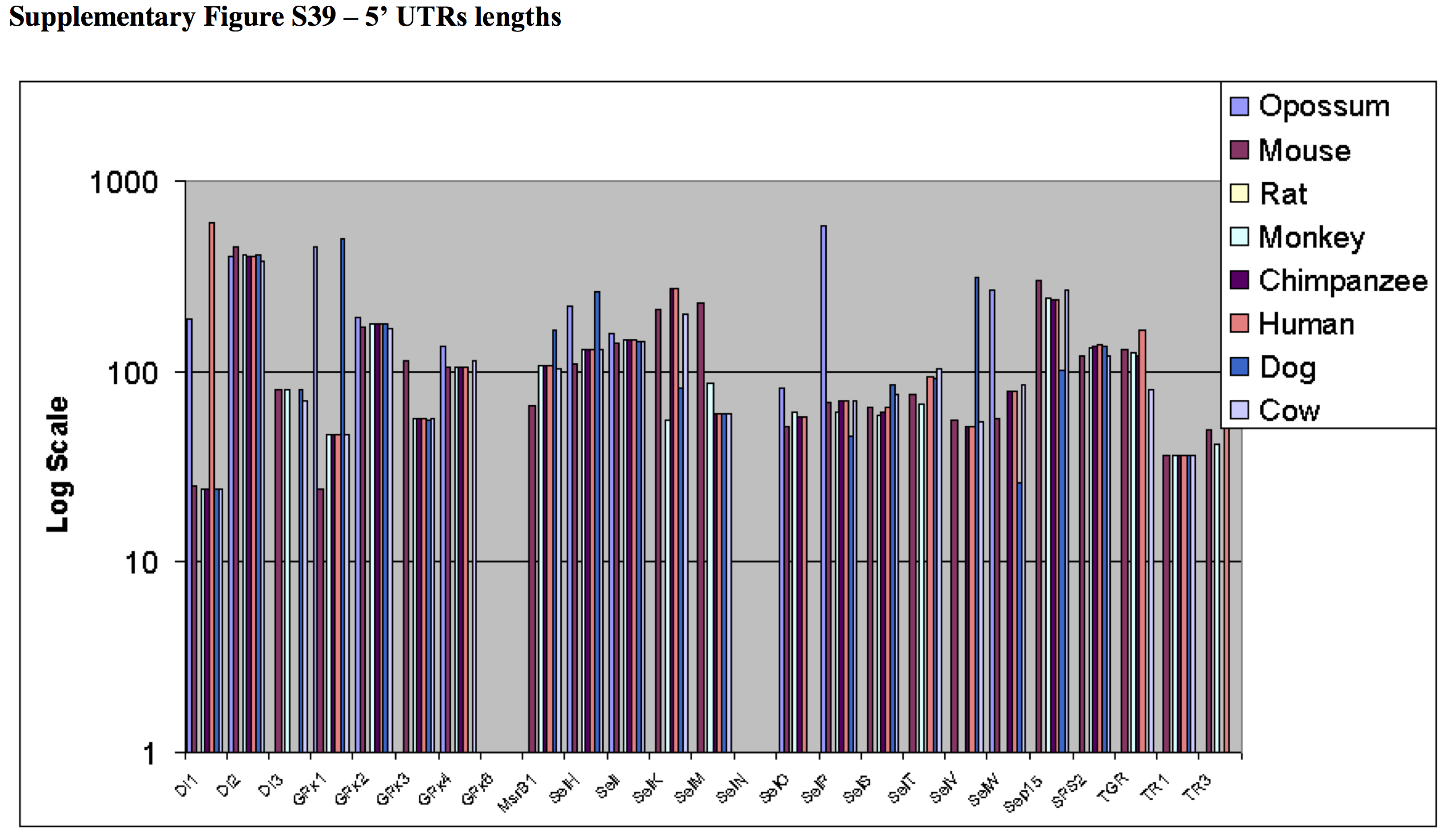

Supplement: Figure S39 — 5′-UTR lengths. 5′-UTR lengths are shown for various mammals. No length means there was insufficient EST data to define the 5′-UTR. (TIFF) [file pone.0033066.s039.tif]

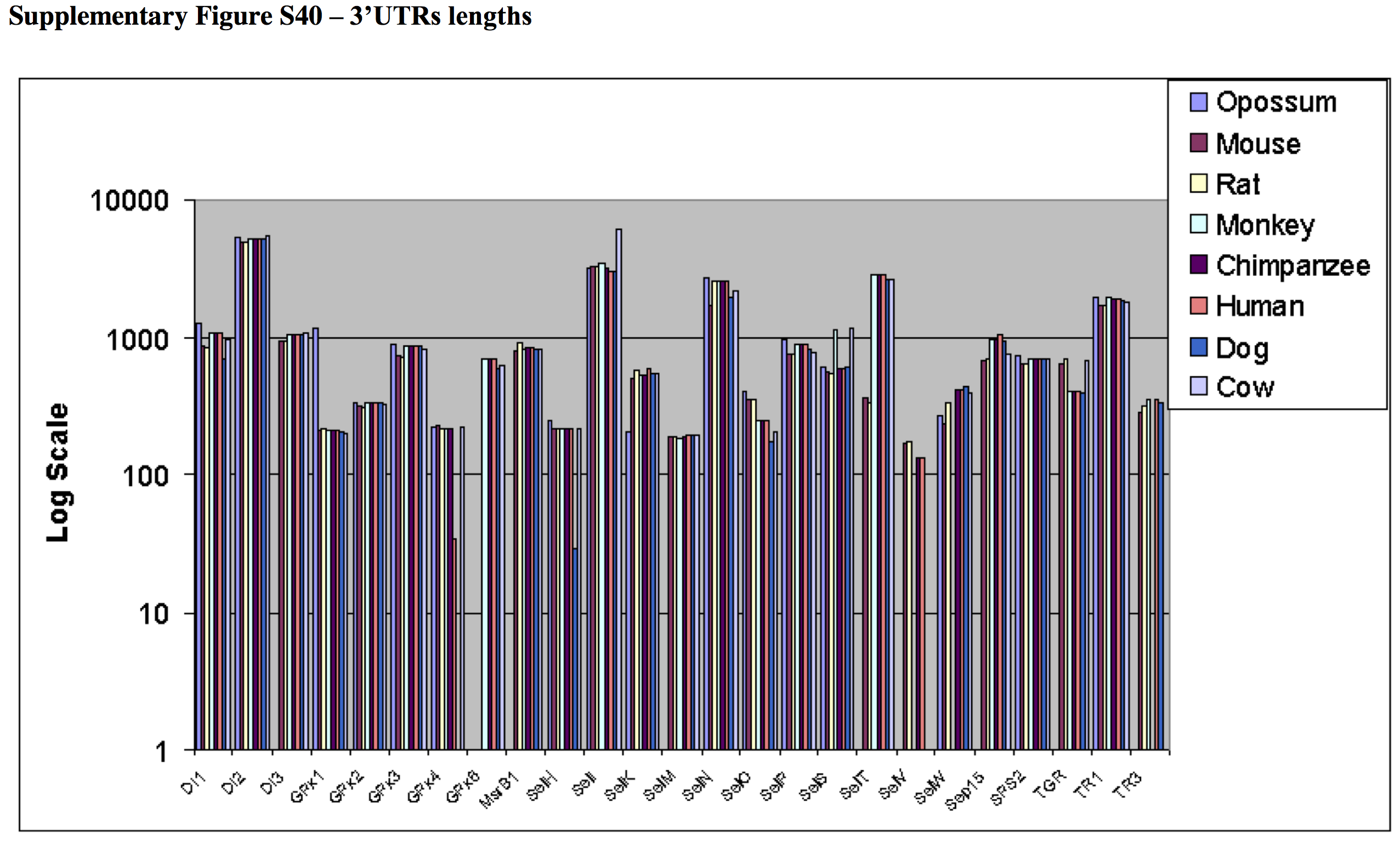

Supplement: Figure S40 — 3′-UTR lengths. 3′-UTR lengths are reported for various mammals. No length means there was insufficient EST data to define the 3′-UTR. (TIFF) [file pone.0033066.s040.tif]

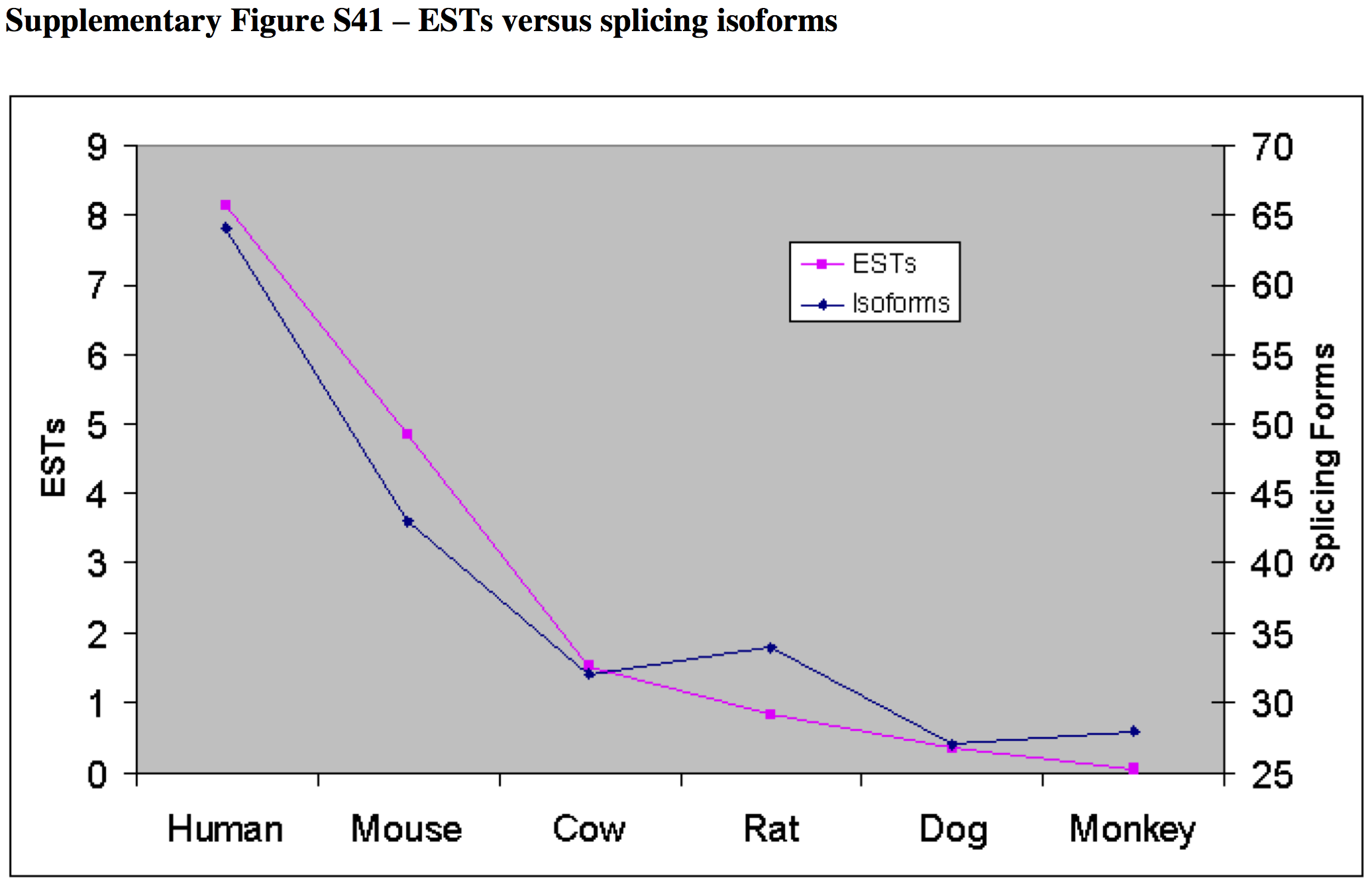

Supplement: Figure S41 — Number of ESTs versus number of splicing forms. Left axis corresponds to the number of available ESTs (millions) and the right axis to the number of identified splicing forms identified in the listed animals. (TIFF) [file pone.0033066.s041.tif]

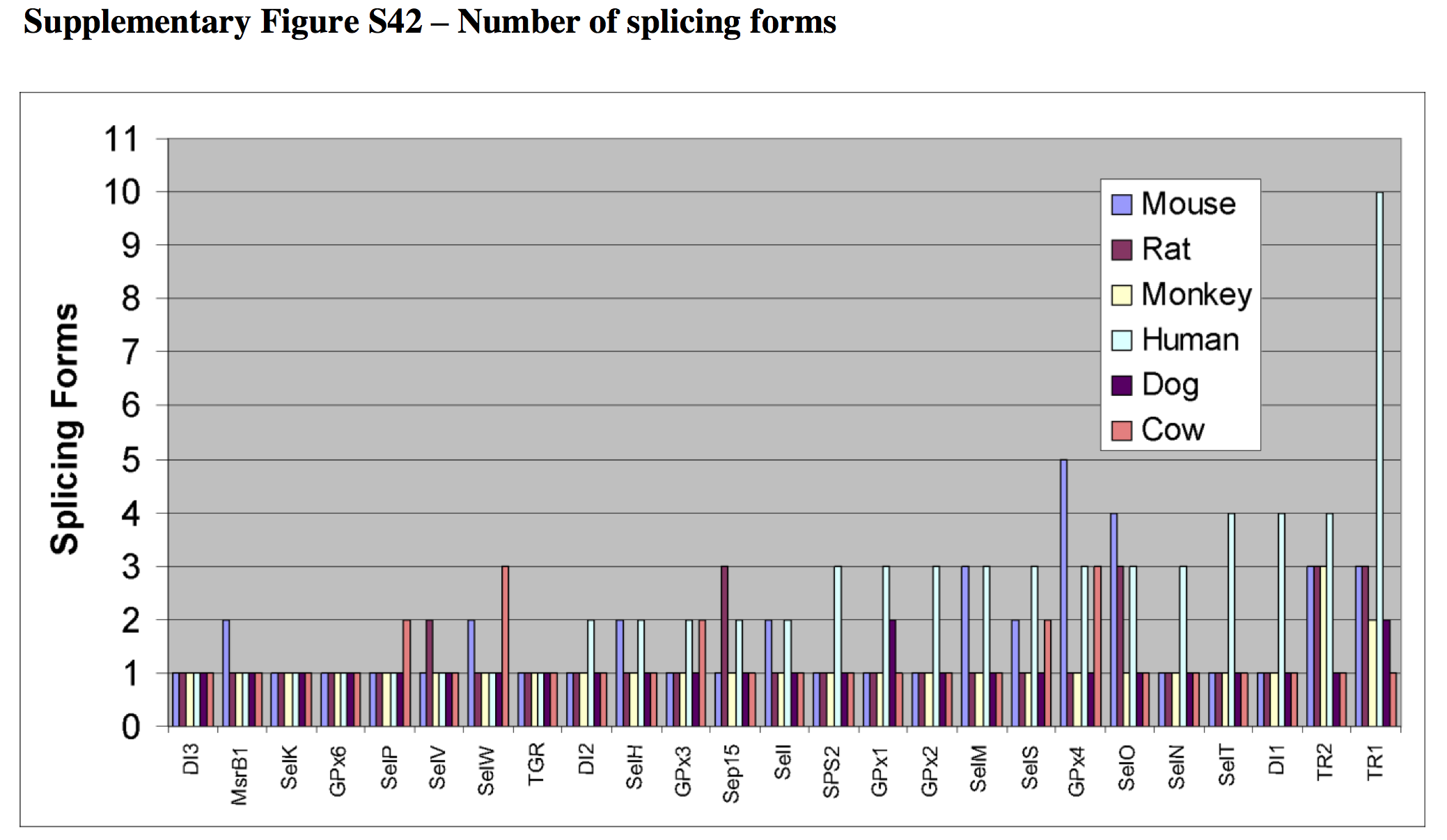

Supplement: Figure S42 — Splicing forms per selenoprotein. The numbers of splicing forms for each of 25 selenoproteins in mammals are shown. (TIFF) [file pone.0033066.s042.tif]
