# Supplementary material for: Composition and Evolution of the Vertebrate and Mammalian Selenoproteomes
Source: PLoS One. 2012 Mar 30;7(3):e33066. doi: 10.1371/journal.pone.0033066 (PMC3316567; doi:10.1371/journal.pone.0033066)
Supplement: Table S1 — Scientific names of species investigated in this study. (DOCX) [file pone.0033066.s043.docx]

**Supplementary Table S1. Scientific names of species investigated in this study.**

| **Common name** | **Scientific name** |
| --- | --- |
| Armadillo | Dasypus novemcinctus |
| Cat | Felis catus |
| Chicken | Gallus gallus |
| Chimpanzee | Pan troglodytes |
| Cow | Bos taurus |
| Dog | Canis lupus familiaris |
| Dolphin | Tursiops truncatus |
| Elephant | Loxodonta africana |
| Elephant shark | Callorhinchus milii |
| Finch | Taeniopygia guttata |
| Frog | Xenopus (Silurana) tropicalis |
| Fugu | Takifugu rubripes |
| Galago | Otolemur garnettii |
| Gorilla | Gorilla gorilla |
| Guinea pig | Cavia porcellus |
| Hedgehog | Erinaceus europaeus |
| Horse | Equus caballus |
| Human | Homo sapiens |
| Hyrax | Procavia capensis |
| Kangaroo rat | Dipodomys ordii |
| Lizard | Anolis carolinensis |
| Llama | Lama pacos |
| Macaque | Macaca mulatta |
| Macrobat | Pteropus vampyrus |
| Marmoset | Callithrix jacchus |
| Medaka | Oryzias latipes |
| Microbat | Myotis lucifugus |
| Mouse | Mus musculus |
| Mouse lemur | Microcebus murinus |
| Opossum | Monodelphis domestica |
| Orangutan | Pongo pygmaeus |
| Pig | Sus scrofa |
| Platypus | Ornithorhynchus anatinus |
| Pufferfish | Tetraodon nigroviridis |
| Rabbit | Oryctolagus cuniculus |
| Rat | Rattus norvegicus |
| Shrew | Sorex araneus |
| Sloth | Choloepus hoffmanni |
| Squirrel | Spermophilus tridecemlineatus |
| Stickleback | Gasterosteus aculeatus |
| Tarsier | Tarsius syrichta |
| Tree shrew | Tupaia belangeri |
| Wallaby | Macropus eugenii |
| Zebrafish | Danio rerio |
